# 1,4-Addition of TMSCCl_3_ to Nitroalkenes: Efficient Reaction Conditions and Mechanistic Understanding

**DOI:** 10.1002/chem.201402394

**Published:** 2014-05-21

**Authors:** Na Wu, Benoit Wahl, Simon Woodward, William Lewis

**Affiliations:** a School of Chemistry, University of Nottingham, University Park Nottingham NG7 2RD (UK)

**Keywords:** brønsted base catalysis, catalysis, Michael addition, reaction mechanisms, trichloromethylation

## Abstract

Improved synthetic conditions allow preparation of TMSCCl_3_ in good yield (70 %) and excellent purity. Compounds of the type NBu_4_X [X=Ph_3_SiF_2_ (TBAT), F (tetrabutylammonium fluoride, TBAF), OAc, Cl and Br] act as catalytic promoters for 1,4-additions to a range of cyclic and acyclic nitroalkenes, in THF at 0–25 °C, typically in moderate to excellent yields (37–95 %). TBAT is the most effective promoter and bromide the least effective. Multinuclear NMR studies (^1^H, ^19^F, ^13^C and ^29^Si) under anaerobic conditions indicate that addition of TMSCCl_3_ to TBAT (both 0.13 M) at −20 °C, in the absence of nitroalkene, leads immediately to mixtures of Me_3_SiF, Ph_3_SiF and NBu_4_CCl_3_. The latter is stable to at least 0 °C and does not add nitroalkene from −20 to 0 °C, even after extended periods. Nitroalkene, in the presence of TMSCCl_3_ (both 0.13 M at −20 °C), when treated with TBAT, leads to immediate formation of the 1,4-addition product, suggesting the reaction proceeds via a transient [Me_3_Si(alkene)CCl_3_] species, in which (alkene) indicates an Si⋅⋅⋅O coordinated nitroalkene. The anaerobic catalytic chain is propagated through the kinetic nitronate anion resulting from 1,4 CCl_3_^−^ addition to the nitroalkene. This is demonstrated by the fact that isolated NBu_4_[CH_2_−NO_2_] is an efficient promoter. Use of H_2_C−CH(CH_2_)_2_CH−CHNO_2_ in air affords radical-derived bicyclic products arising from aerobic oxidation.

## Introduction

**Scheme 1:**
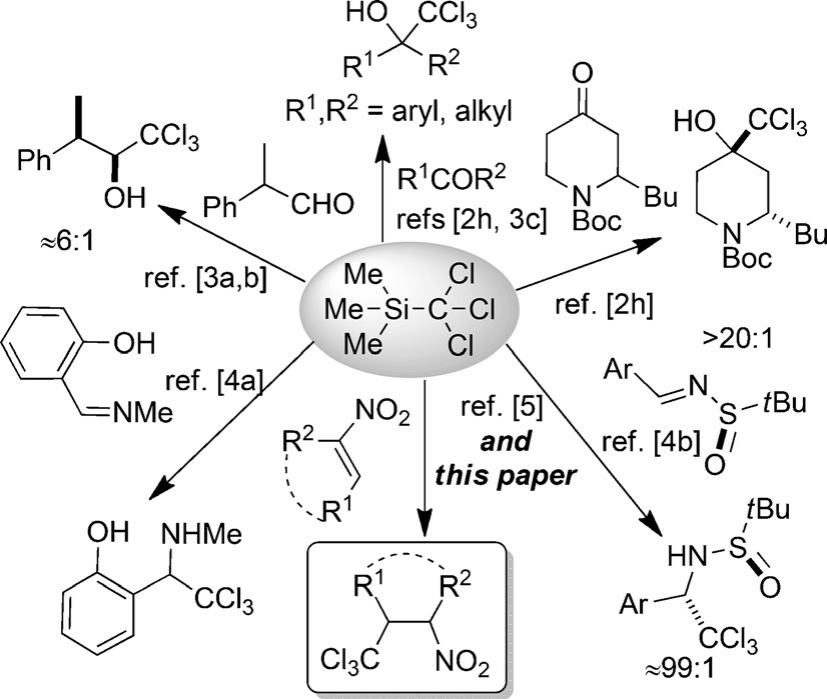
Known 1,2- versus 1,4-additions of TMSCCl_3_. Boc=*tert*-butoxycarbonyl.

Despite its potential for use in organic synthesis, applications of TMSCCl_3_ (TMS=SiMe_3_) have been far narrower in scope than those of closely related TMSCF_3_ (Ruppert–Prakash reagent).^1^ Two reasons can be identified as the origins of this situation. Firstly, all present literature preparations of TMSCCl_3_ provide either low-to-modest isolated yields,^2^ or rely on extreme low-temperature protocols (typically −110 °C),^2^ limiting easy access to this reagent. Secondly, most applications of TMSCCl_3_ require its “activation” by a silylphilic promoter, typically a fluoride ion. The intimate mechanism(s) by which this process proceeds are presently based on ad hoc suggestions rather than tangible data. In such environments it is possible to select reaction conditions that may not be mechanistically optimal. Although TMSCCl_3_ is known to participate in a small number of 1,2-additions to aldehydes,^3^ ketones^3^ and imine derivatives (Scheme 1),^4^ 1,4 addition modes are practically unknown and are limited to just six examples, with modest yields, in a single paper by Cunico and Zhang.^5^ We were interested to identify improved experimental conditions for such reactions and to understand the underlying mechanism of 1,4-addition of TMSCCl_3_. New access to β-CCl_3_-substituted nitroalkanes is of interest, and Sosnovskikh et al., and others,^6^ have developed a range of unusual and useful methods around this motif.

## Results and Discussion

**Scheme 2:**
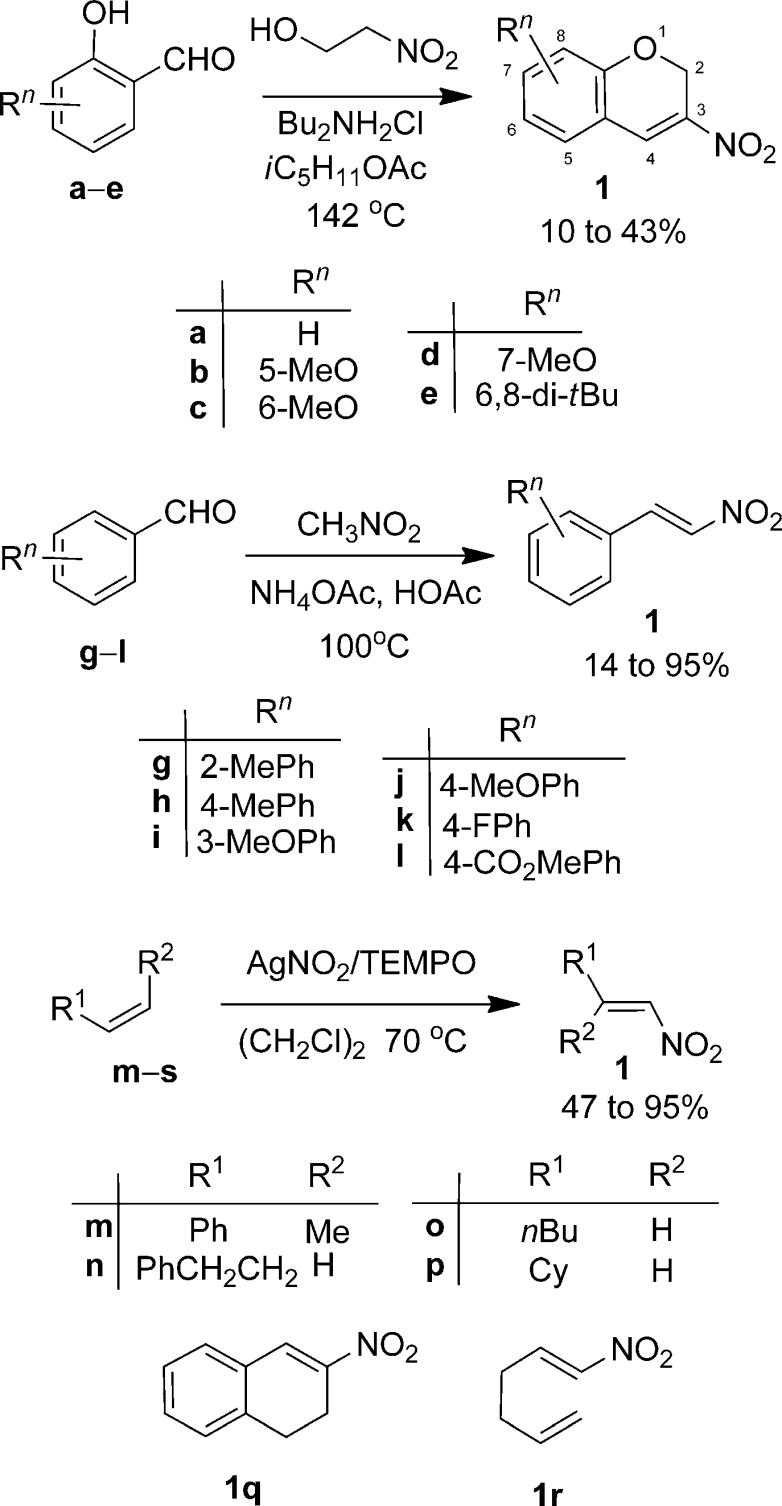
Preparation of nitroalkene starting materials. TEMPO=2,2,6,6- tetramethylpiperidine *N*-oxide; Cy=cyclohexyl.

For the conjugate-addition mechanistic investigations we required access to large amounts of highly pure TMSCCl_3_. Unfortunately, current literature preparations^2^ have significant limitations, either in yield, purity of reagent attained, reproducibility or the need for special conditions. These issues are due to the fragility of MCCl_3_ (typically M=Li or Na) intermediates. Facile decomposition of these intermediates leads to dichlorocarbene-derived byproducts. We reasoned that the use of low-cost TMSCl (ca. € 0.1 per mL) as a co-solvent (5 equiv) would significantly enhance CCl_3_^−^ capture, improving the TMSCCl_3_ yield. Addition of LiHDMS (lithium hexamethyl disilazide) in hexane/THF (65:20) to a chloroform/TMSCl (12:64) mixture at −65 °C, followed by slow warming to ambient temperature proved optimal. After an appropriate workup, Kugelrohr sublimation routinely afforded pristine material in 64–70 % yield on a >10 g scale. The nitroolefins, **1**, for our study were prepared by using a one-pot procedure by Dauzonne and Royer,^7^ a condensation method by Andrew and Raphael^8^ or by using a very recent AgNO_2_ method by Maiti et al.^9^ (Scheme 2). The advantage of the former two methods, although the yields are often modest, is that they are technically simple and provide a direct route to the 3-nitro-2*H*-chromenes and styrenyl systems, respectively. The advantage of the latter procedure is its wide and general scope. Initial studies on substrate **1 a** (Table 1) confirmed the findings of Cunico and Zhang,^5^ but indicated that CsF is an unreliable promoter because of its low solubility in organic solvents. Soluble NBu_4_X [X=Cl, OAc, F and especially Ph_3_SiF_2_ (TBAT)] were found to be efficient promoters at 5 mol % in both polar (THF, entries 2–5) and non-polar (toluene, entries 6–10) solvents. The less-silylophilic promoter, NBu_4_Br, led to a very slow turnover. Due to the speed of the reactions in THF at 20 °C no rate estimates could be attained (entries 2–5). However, approximate initial rates (based on conversion in the first 20 sec) could be attained in toluene, confirming the high efficacy of TBAT; in the absence of any promoter no reaction occurred. The spectroscopic properties of **2 a** are in accord with 1,4-addition of the trichloromethyl group. In particular, a characteristic multiplet is seen at *δ*_H_=5.56 ppm in the ^1^H NMR spectrum, correlating to the ^13^C NMR CH signal α to the nitro group at *δ*_C_=80.7 ppm. The β-CH group is diagnostically shifted to lower frequency (*δ*_C_=56.3 ppm in **2 a**) compared with its C−CH precursor (*δ*_C_=139.2 ppm in **1 a**), whereas a low-intensity quaternary signal at *δ*_C_=100.8 ppm is assigned to CCl_3_ and the molecular ion of **2 a** shows the expected Cl_3_ isotope pattern.

**Table 1:** Promoter comparison for TMSCCl_3_ addition to 1 a.^[a]^

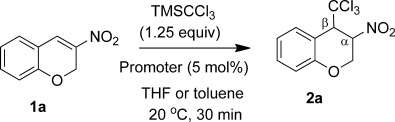				
Entry	Promoter	Solvent	2 a[%]	Rate (rel.) [s^−1^]
1	CsF	either^[b]^	<10^[c]^	n.d.
2	NBu_4_Cl	THF	>95	n.d.
3	NBu_4_OAc	THF	>95	n.d.
4	NBu_4_F	THF	>95	n.d.
5	NBu_4_Ph_3_SiF_2_	THF	>95	n.d.
6	NBu_4_Br	toluene	37	2.6×10^−5^ (0.03)
7	NBu_4_Cl	toluene	>95	7.6×10^−4^ (1.0)
8	NBu_4_OAc	toluene	>95	1.4×10^−3^ (1.8)
9	NBu_4_F	toluene	>95	2.0×10^−3^ (2.7)
10	NBu_4_Ph_3_SiF_2_	toluene	>95	2.5×10^−3^ (3.3)

[a] Carried out on 0.3 mmol **1 a** (0.06 M). Yield data obtained from GC analysis in the presence of an internal standard (1-methylnaphthalene, 25 μL, 0.18 mmol). [b] Use of either solvent resulted in inefficient catalysis; [c]>95 % of **2 a** could only be attained after 24 h in THF with excess CsF (3.75 equiv). n.d.=not determined.

**Scheme 3:**
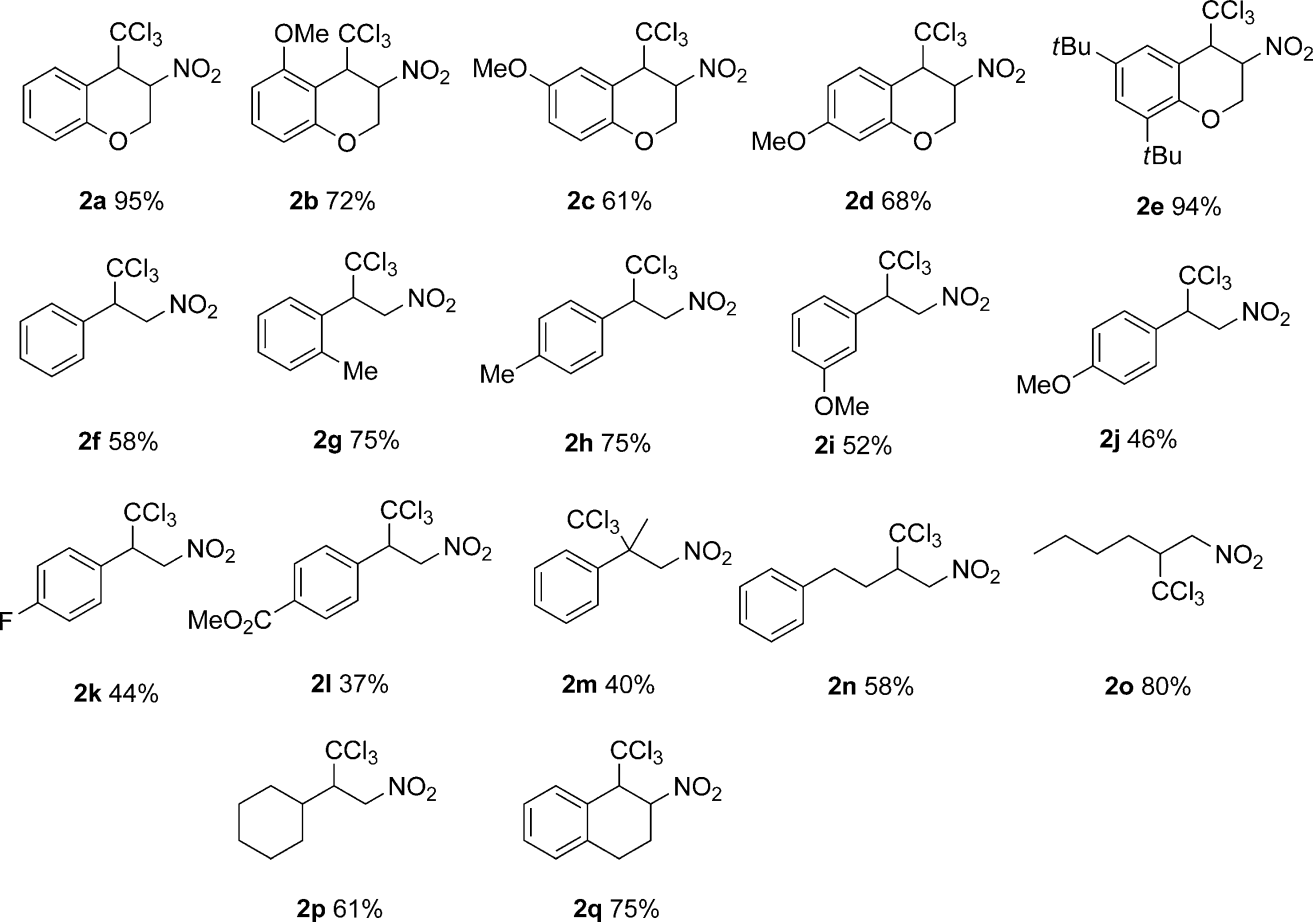
Isolated addition products from TBAT-catalysed (5 mol %) additions of TMSCCl_3_ to nitroalkenes 1.

The conditions of Table 1 (entry 5) could be applied to a range of nitroalkene substrates, leading to various 1,4-addition products in 37–95 % isolated yields (Scheme 3). For the acyclic systems reversal of the addition mode proved optimal.

**Figure 1:**
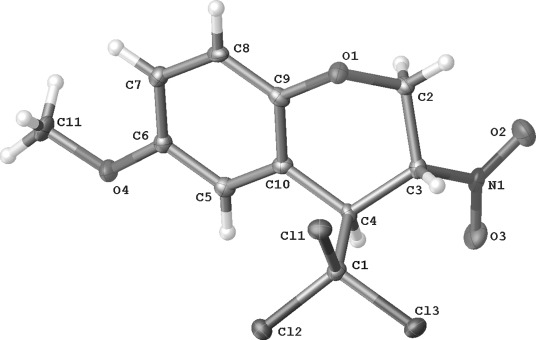
Molecular structure of 2 d. Selected interatomic distances and angles: C(1)−C(4) 1.556, C(3)−C(4) 1.544, N(1)−C(3) 1.519 Å; C(1)-C(4)-C(3) 109.2, N(1)-C(3)-C(4) 111.3 ^o^. Dihedral angle N(1)-C(3)-C(4)-C(1) 115.4 ^o^.

The connectivity of **2 d** could be confirmed by an X-ray crystallographic study (Figure 1). In comparison, the 27 structures in the Cambridge Crystallographic Database^6^ showing the same NO_2_C^α^HC^β^HCCl_3_ motif have: N−C^α^ 1.49–1.53, C^α^−C^β^ 1.52–1.55 and C^β^−CCl_3_ 1.51–1.57 Å; N- C^α^-C^β^ 105.8–117.1 and C^α^-C^β^-CCl_3_ 111.2–117.1 ^o^. Two closely related six-ring structures (CIBGIF and HACJAY) show *anti* arrangements, as in **2 d**, but a *syn* motif is also known (QEMZUE).^6^

**Scheme 4:**
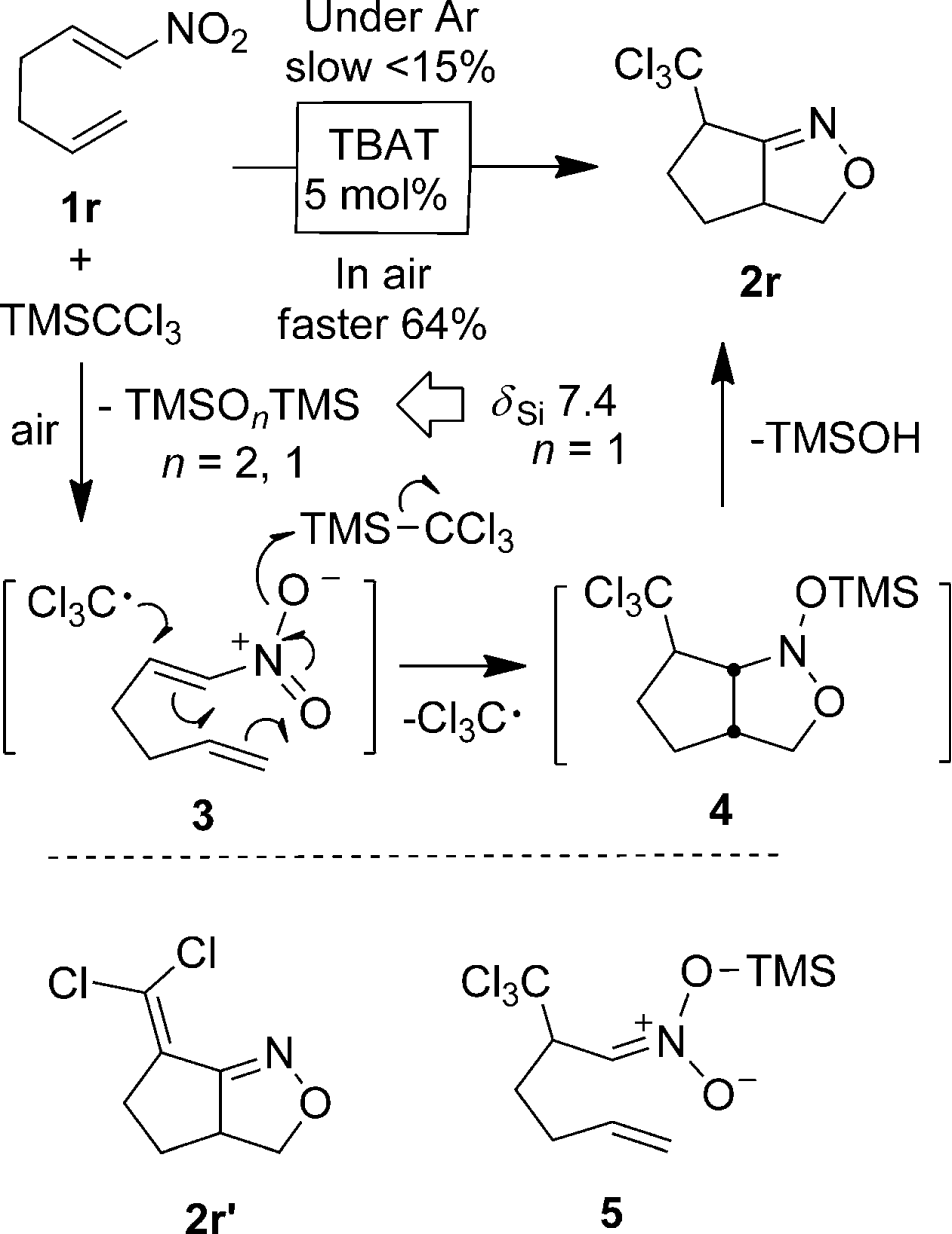
Aerobic cyclisation of substrate 1 r.

**Figure 2:**
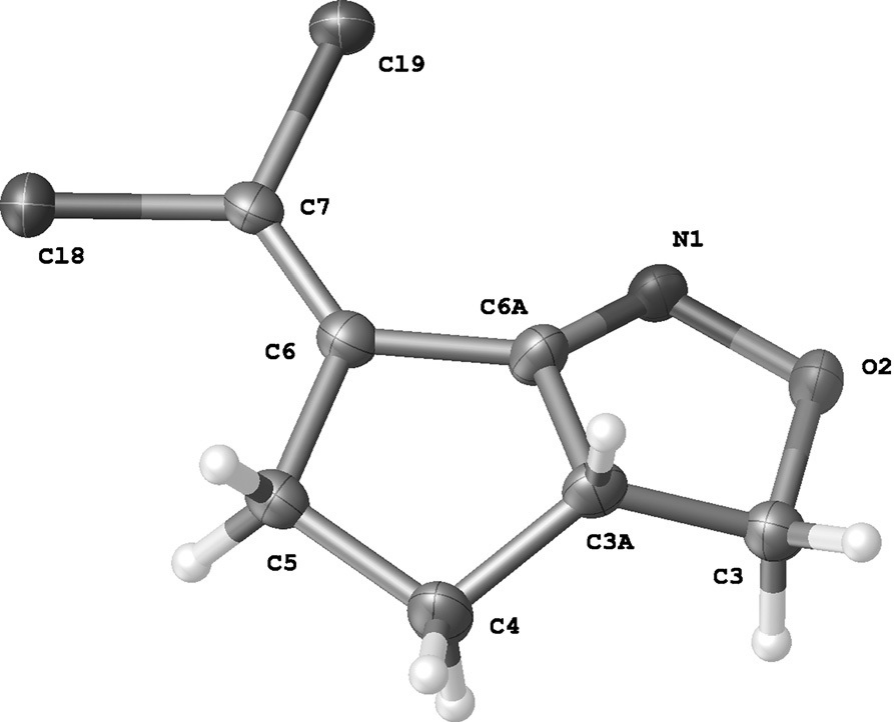
Molecular structure of 2 r′. Selected interatomic distances and angles: N(1)−C(6A) 1.258, N(1)−O(2) 1.431, C(6)−C(7) 1.324 Å; C(6A)-N(1)-O(2) 107.5, C(7)-C(6)-C(6A) 127.1 ^o^.

The following scope and limitation comments should be made: i) Addition of TMSCCl_3_ to the substrate and TBAT catalyst was appropriate for α-substituted substrates **1 a**–**e** and **1 q**. However, for terminal nitroalkenes (**1 f**–**p**) the alkene needed to be added slowly (over 1 h) to TMSCCl_3_/TBAT mixtures to avoid polymerisation, which led to unacceptable yields of **2**. ii) Acyclic α-substituted nitroalkenes led to very poor reactions and this substrate class was not further pursued; β-substitution provided clean products (e.g. **2 m**), but in reduced yield. iii) Although alkyl, ether, ester, alkene and C−F functional groups were tolerated in various degrees, some aryl bromide-containing substrates (e.g. 8-bromo-3-nitro-2*H*-chromene) and benzylic (*E*)-BnCH−CHNO_2_ were not tolerated and led only to decomposition. Given the known relative stability of the CCl_3_ radical, and its propensity to add to unsaturated systems (Kharasch et al.^10^), tests were carried out to assess the veracity of such reaction pathways. Firstly, it was observed that the presence of the known radical inhibitors hydroquinone or butylated hydroxytoluene (BHT) did not prevent TBAT-catalysed additions to **1 d** under strictly anaerobic conditions. Secondly, the substrate **1 r** was used to provide potential intramolecular radical trapping sites. The standard reaction conditions (slow addition of **1 r** to TMSCCl_3_/TBAT under argon) led to trace amounts (15 %) of bicyclic **2 r**, and the majority of the starting material remained unconverted after the typical reaction time of 1–16 h (Scheme 4). However, if the reaction was conducted under aerobic conditions **2 r** became the major product. TMSCCl_3_ solutions in THF, in the presence of TBAT and O_2_ (one molar equivalent of oxygen injected into a sealed reaction), were analysed by ^29^Si NMR spectroscopy and revealed a significant amount of a single silicon-containing species showing *δ*_Si_=7.4 ppm. Based on comparison with literature silicon NMR shift values^11^ we assigned this new species as TMS_2_O. One explanation for the formation of **2 r** is reaction of TMSCCl_3_ (in the presence of TBAT) with O_2_, leading to TMS_2_O_2_ and CCl_3_ radicals that cascade to **2 r**, via **3** and **4**. Although we could not detect any TMS_2_O_2_ peroxide (*δ*_Si_≈−27 ppm^12^) in air or in O_2_-exposed samples of TMSCCl_3_, in the presence or absence of TBAT, the latter was rapidly converted to TMS_2_O. It is likely that any peroxide would be both generated and decomposed as shown in Scheme 4. The siloxane can also be generated from TMSOH generated by elimination from **4**. Literature bicycles related to **2 r** have been generated, either by oxidation of nitronate anions^13^ or through nitrile oxide formation and subsequent [2+3] cycloaddition chemistry,^13^ from the expected 1,4-addition product **5**. Although we cannot completely exclude such possibilities, such approaches normally require stronger oxidants than molecular oxygen or prolonged heating at 60 °C.^13^ The connectivity of **2 r** could be confirmed by an X-ray crystallographic study on its dehydrochlorination product **2 r′**, obtained through simple MgSO_4_ drying/recrystallisation of **2 r** (Figure 2). Formation of the C−CCl_2_ bond is also evident in the ^13^C NMR spectrum, in which the diagnostic CCl_3_ signal at *δ*_C_=99.4 ppm in **2 r** is replaced by two quaternary alkene signals (*δ*_C_=121.4 and 126.7 ppm). Both **2 r** and **2 r′** have a C−N resonance (*δ*_C_=167.6 and 166.5 ppm, respectively). The structure of **2 r′** is reminiscent of other tetrahydro-3*H*-cyclopenta[c]isoxazoles in the Cambridge Crystallographic Database.^14^

**Figure 3:**
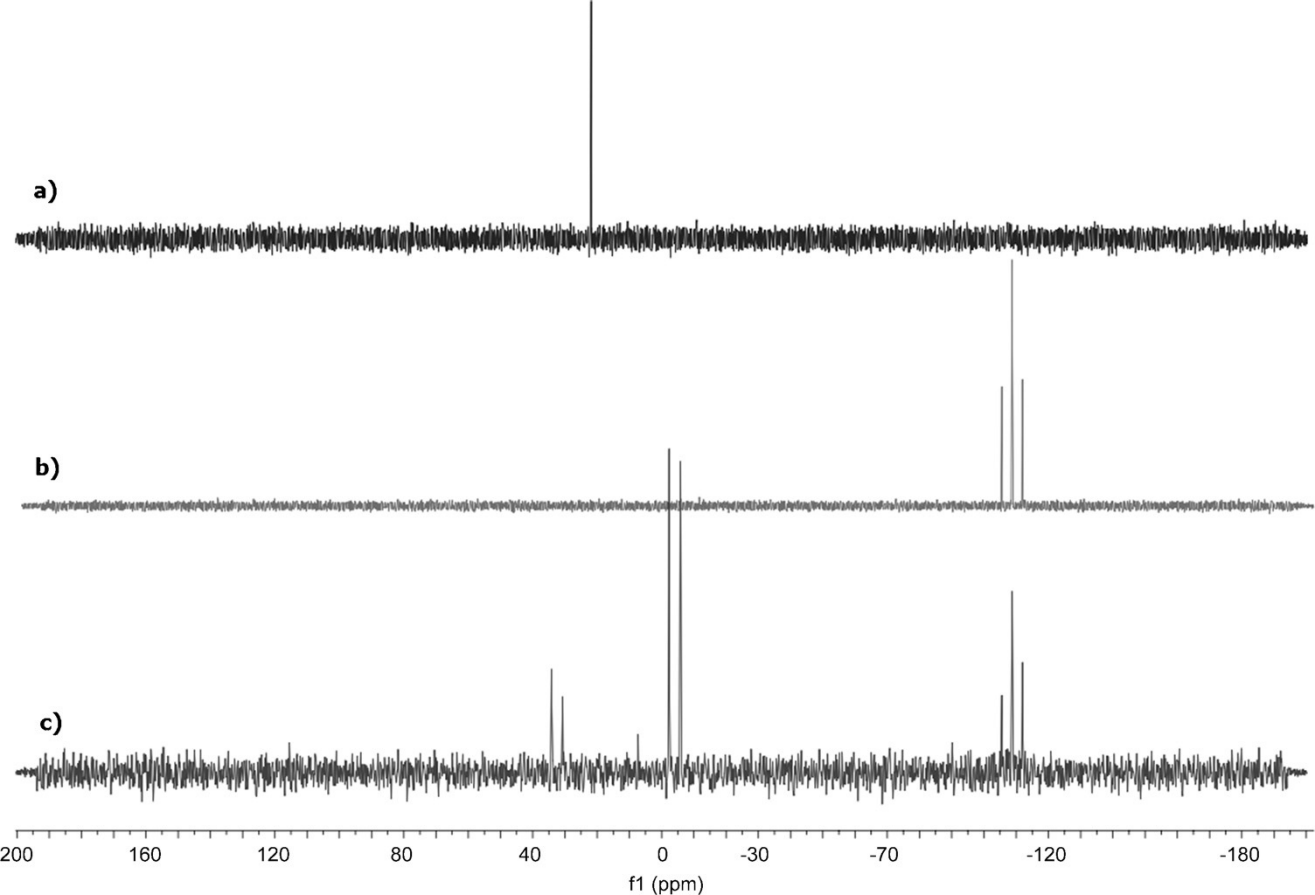
^29^Si NMR spectra (79.5 MHz, 5:1 THF/[D_6_]benzene, −20 °C): a) TMSCCl_3_ (*δ*=21.9 ppm); b) TBAT (*δ*=−108.8 ppm, *J*(Si−F)=254 Hz); c) a nominal 1:1 mixture of TMSCCl_3_/TBAT (a slight excess of TBAT was used to provide a spectral internal standard), TMSF (*δ*=32.4 ppm, *J*(Si−F)=275 Hz), TMS_2_O (*δ*=7.3 ppm), Ph_3_SiF (*δ*=−4.1 ppm, *J*(Si−F)=282 Hz).

Despite the aerobic cyclisation shown in Scheme 4, we could not attain any evidence for radical involvement under strict anaerobic conditions. In particular, the observation of low, but reproducible, *ee* values in the formation of **2 q** by using chiral promoters at low substrate conversion suggested that a different reaction mechanism operates under O_2_-free conditions. To the best of our knowledge, no study of the explicit reaction mode of TMSCCl_3_ with NBu_4_SiPh_3_F_2_ (TBAT) has been carried out, therefore, we sought to define the non-radical process by multi-nuclear NMR studies. Reagent concentrations of 0.125 M in THF/[D_6_]benzene (5:1) at −20 °C offered the best compromise with respect to solubility, ^29^Si sensitivity and attainment of O_2_-free conditions. Temperatures of −20 °C are also the lowest at which viable catalytic reactions are possible, indicating that the reactions should be slowed to only primary events at this temperature. Representative ^29^Si NMR spectra are given in Figure 3. In an initial set of conditions at −20 °C, TMSCCl_3_ (*δ*_Si_=21.9 ppm) was immediately converted to TMSF (*δ*_Si_=32.4 ppm)^15^ on addition of TBAT (*δ*_Si_=−108.8 ppm),^16^ which itself was transformed to Ph_3_SiF (*δ*_Si_=−4.1 ppm)^17^ (Figure 3). No other silicon-containing species were present, except for traces of TMS_2_O (*δ*_Si_=7.3 ppm)^11^ (which could be minimised/eliminated by good experimental technique to eliminate the last traces of O_2_). The *J*_SiF_ coupling pattern is indicative of the number of attached fluorine atoms in these species. The residual TBAT species, TMSF and Ph_3_SiF could be correlated to signals at *δ*_F_=−98.9,^17^ −158.0^15^ and −170.5 ppm^16^ in the ^19^F NMR spectrum of the reaction mixture at −20 °C (see the Supporting Information). This accounted for >98 % of all the fluorine-containing species. The ^29^Si and ^19^F NMR spectra of the same reaction mixture at +20 °C show only very slight broadening, indicating that any exchange between the species detected is, at best, very slow under the reaction conditions. At −20 °C the ^13^C NMR spectrum of the TMSCCl_3_/TBAT mixture, in the methyl region, confirmed the presence of Me_3_SiF (*δ*_C_=−0.4 ppm, *J*_CF_=15.5 Hz) and a singlet peak (*δ*_C_=1.6 ppm) ascribed to the expected exchange product, NBu_4_CCl_3_. This latter compound is stable at −20 °C indefinitely, no evidence of formation of tetrachlorethene (*δ*_C_=120.7 ppm), or any other CCl_3_^−^ or dichlorocarbene-derived byproducts was seen in the spectra. No reaction was observed when nitroalkene **1 a** was added last to the above mixture, which was then warmed from −20 to 0 °C (conditions under which the catalytic reaction is spontaneous).

**Figure 4:**
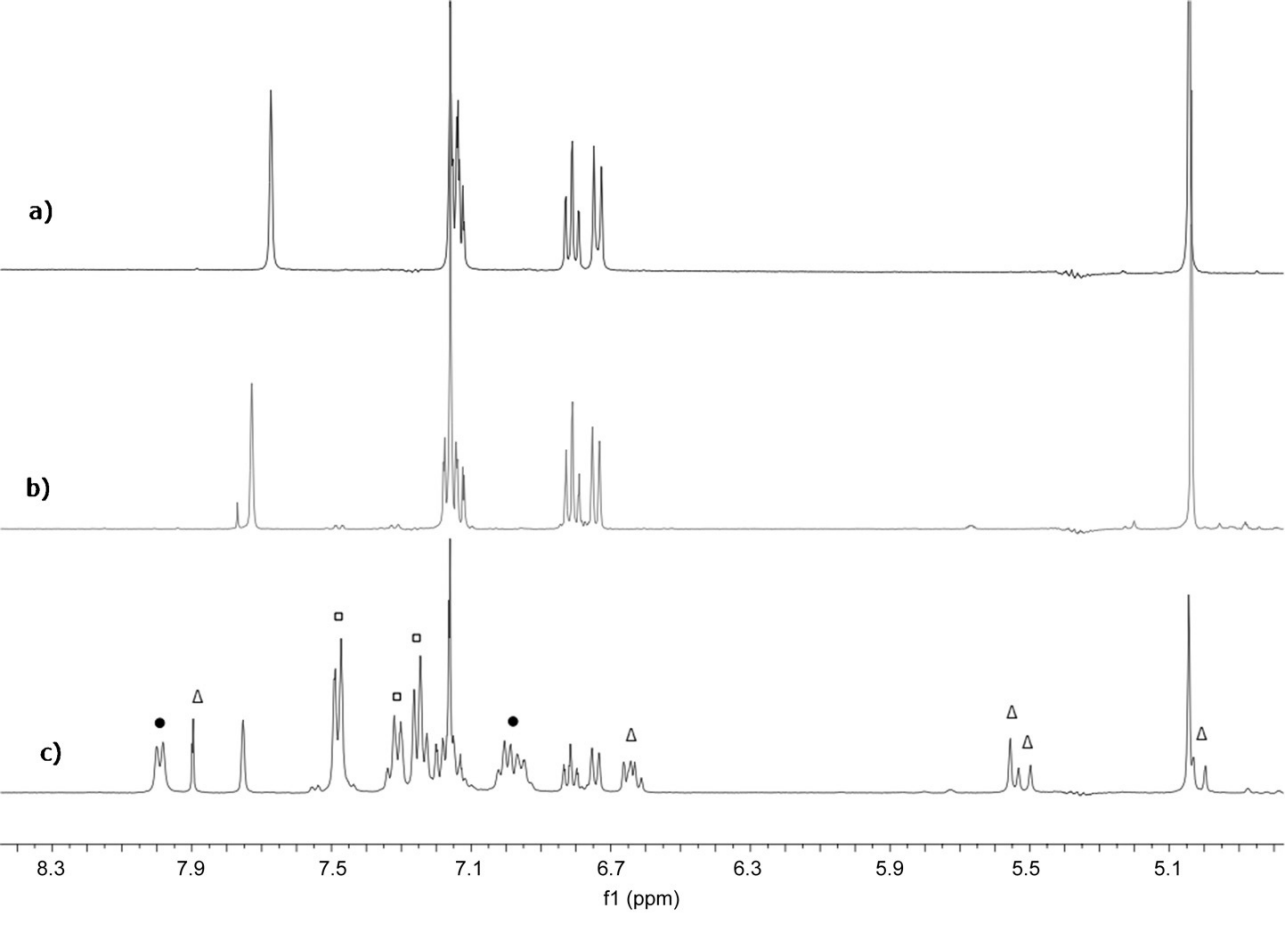
Partial ^1^H NMR spectra (5:1 THF/[D_6_]benzene, −20 °C): a) alkene 1 a; b) a nominal 1:1 mixture of alkene 1 a and TMSCCl_3_; c) After addition of 1 equiv of TBAT to mixture (b). Signals due to TBAT are marked (•), those due to Ph_3_SiF (□) and those due to the proposed nitronate product 8 (Δ).

**Scheme 5:**
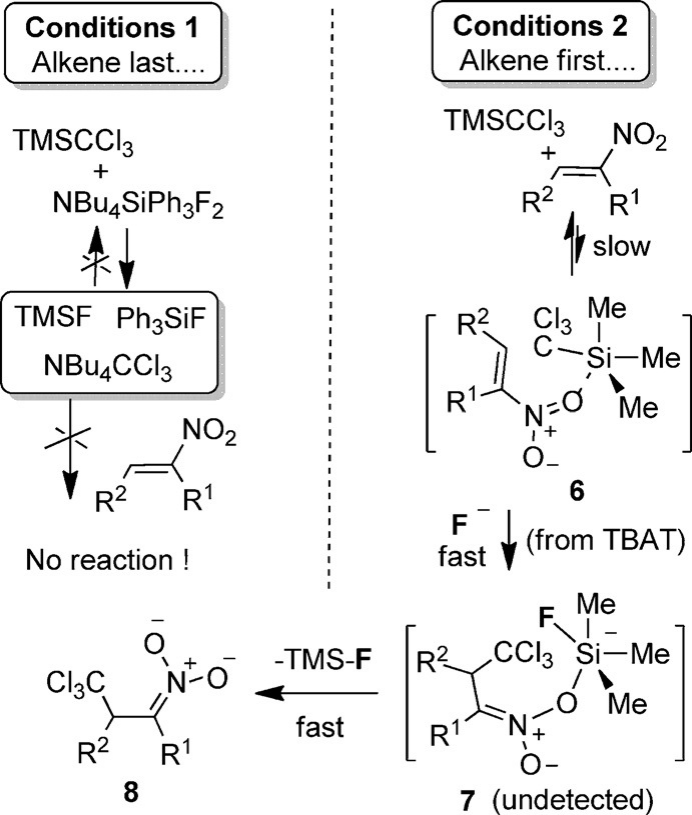
Mechanistic proposal from stoichiometric NMR studies.

In a separate set of conditions, nitroalkene **1 a** was first added to TMSCCl_3_ at −20 °C. The ^1^H NMR spectrum in the region *δ*_H_=6.8–8.0 ppm contains the aryl and alkene signals of **1 a** (see Figure 4 a,b). The signal of TMSCCl_3_ is at *δ*_H_=0.20 ppm (not shown in Figure 4). The equivalent ^13^C NMR spectra confirm <5 % reaction of alkene and TMSCCl_3_ because only the characteristic peaks of **1 a** and TMSCCl_3_, at *δ*_C_=−4.5 ppm are present. The ^29^Si NMR spectrum of the reaction mixture shows only the presence of TMSCCl_3_. Subsequent addition of TBAT at −20 °C to this mixture leads to partial (38 %), but immediate, conversion of **1 a** into a new compound with ^1^H (Figure 4 c) and ^13^C NMR spectra (see the Supporting Information) that are closely related to those of the addition product **2 a**. This new species is assigned as nitronate **8** (Scheme 5). In some experiments traces of a new species could be detected before the addition of TBAT (See Figure 4 b). This species could only be identified as a product of nitroalkene decomposition, or species **6** (see below).

**Scheme 6:**
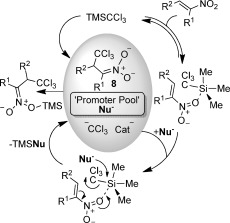
Proposed catalytic cycle.

Overall, the data from the stoichiometric NMR experiments are in accord with Scheme 5. Once formed, NBu_4_CCl_3_ is insufficiently nucleophilic to directly attack the nitroalkene and equilibration back to TMSCCl_3_ is not possible. Rather, the nitroalkene binds TMSCCl_3_ by means of an electron-rich N−O contact, providing **6**. We could detect no exchange broadening of the minor signals observed in Figure 4 b from ambient temperature down to −20 °C. Further cooling of the reaction was not possible because of the formation of non-homogeneous samples, and heating the sample was not possible. However, the data are generally in accord with attack of an external nucleophile, in this case fluoride ions (either directly or indirectly from TBAT^18^), on **6**, triggering CCl_3_ transfer, presumably through a chair-like transition state, leading to **7**. Finally, due to the strength of the Si−F bond in TMSF (and the clear lack of exchange with this species in the NMR studies) it is nitronate **8** that is expelled and detected spectroscopically. If the conclusions of the stoichiometric reactions shown in Scheme 5 can be translated to the catalytic reactions, three clear predictions can be made: i) Nitronate anions themselves should be excellent promoters of the reaction. ii) If the concentration of nitronate (or indeed any other anionic promoter nucleophile) builds up over time, a wide range of nucleophilic promoters (Nu^−^) will be available to replace fluoride (F^−^) in the key conversion of **6** into **7** (Scheme 5). iii) Under such conditions, the *ee* value of the 1,4-addition product, produced by an asymmetric source of Nu^−^ should decrease over time (due to competition with an increasingly populated pool of promoter anions). To check these suppositions we prepared NBu_4_[CH_2_−NO_2_] from nitromethane and found that it does indeed promote rapid quantitative conversion of **1 a** into **2 a**. The chiral complex [Ni(Duphos)_2_](acac)_2_ (Duphos=(*R*,*R*)-methylDuphos, CAS [147253-67-6]; acac=acetylacetonate) was found to be an, albeit poor, promoter of asymmetric trichlomethylation of **1 q** (see the Supporting Information). Nevertheless, the *ee* value of **2 q** formed by using this catalyst (10 mol %) does decrease reproducibly from 15 to <1 % over 20 h, in line with predictions. The bifunctional chiral catalyst by Takemoto et al.^19^ (10 mol %), associated with TBAF as a co-promoter (10 mol %), also led to a decrease in the *ee* value of **2 q** over time (from 26 % to <1 %). Based on all of the data it seems likely that Scheme 6 is the most rational description of the catalytic cycle. The nitronate product, **8**, can either act as a promoter itself or leave the promoter pool by means of a reaction with either TMSX (X=CCl_3_ or Nu, if Nu is a suitable leaving group).

**Scheme 7:**
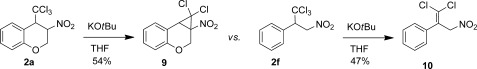
Representative reactions of the 1,4-addition products.

Finally, the use of the 1,4-addition products, **2**, for the formation of other products was briefly investigated. Treatment of **2 a** with KO*t*Bu in THF led to the formation of dichlorocyclopropane **9** in moderate yield (Scheme 7), through α-CH deprotonation. However, the equivalent acyclic systems showed a distinct preference for β-CH deprotonation. For example, **2 f** led to the formation of dichloroalkene **10** when treated under the same conditions.

## Conclusion

Catalytic 1,4-additions of TMSCCl_3_ to electron-deficient Michael acceptors have considerable potential for use in organic chemistry. The mechanistic studies presented here are consistent with pre-coordination of the nitroalkene to the silicon reagent before its promotion by a silylphilic nucleophile. Attempts to develop asymmetric versions of this reaction will prove to be challenging for mechanistic reasons. The kinetically derived nitronate product of the reaction is itself a highly effective chain carrier, and attaining a competitive chiral catalyst or alternative conditions will be critical for success. Investigations into such approaches and the use of other acceptors are underway.

## Experimental Section

Full details of all transformations and associated spectroscopic data are given in the Supporting Information. Nitroolefins **1 a**–**e** were prepared by Dauzonne and Royer’s one-pot procedure^7^. Alkene **1 a** showed identical spectroscopic properties to previously reported samples.^20^ Compounds **1 b**–**e**, previously unreported, were fully characterised (see the Supporting Information). Nitroolefins **1 g**–**l** were prepared by Andrew and Raphael’s condensation method^8^ and had identical spectroscopic properties to previously reported samples.^9^, ^21^, ^22^ Nitroolefins **1 m**–**r** were prepared by the AgNO_2_ method by Maiti et al.,^9^ and had identical spectroscopic properties to previously reported samples.^9^, ^23^

### General procedure for trichloromethylation of cyclic substrates

Trimethyl(trichloromethyl)silane (TMSCCl_3_; 0.21 g, 1.1 mmol) in THF (2 mL) was added dropwise to a solution of the cyclic nitroalkene (1 mmol) and tetrabutylammonium triphenyldifluorosilicate (TBAT; 0.027 g, 5 mol %) in THF (2 mL) under argon at room temperature, and the reaction mixture was stirred overnight. The mixture was concentrated in vacuo and then purified by flash chromatography on silica gel to give the corresponding Michael addition products. Alternatively, the reactions were quenched with saturated NH_4_Cl (aq), extracted with ethyl acetate, dried over anhydrous MgSO_4_ and concentrated before purification by chromatography.

### General procedure for trichloromethylation of acyclic substrates

The acyclic alkene (1 mmol) in THF (2 mL) was added dropwise, over a period of one hour, to a solution of trimethyl(trichloromethyl)silane (TMSCCl_3_; 0.21 g, 1.1 mmol) and tetrabutylammonium triphenyldifluorosilicate (TBAT; 0.027 g, 5 mol %) in THF (2 mL) under argon at room temperature, and the mixture was stirred overnight. The mixture was concentrated in vacuo and then purified by flash chromatography on silica gel to give the corresponding Michael addition products.

## Supporting Information

As a service to our authors and readers, this journal provides supporting information supplied by the authors. Such materials are peer reviewed and may be re-organized for online delivery, but are not copy-edited or typeset. Technical support issues arising from supporting information (other than missing files) should be addressed to the authors.

miscellaneous_information
