# Supplementary material for: 1,4-Addition of TMSCCl3 to Nitroalkenes: Efficient Reaction Conditions and Mechanistic Understanding
Source: Chemistry. 2014 May 21;20(25):7718–24. doi: 10.1002/chem.201402394 (PMC4586478; doi:10.1002/chem.201402394)
Supplement: Supplementary file 1 — miscellaneous_information [file chem0020-7718-sd1.pdf]

CHEMISTRY
A European Journal

Supporting Information

© Copyright Wiley-VCH Verlag GmbH & Co. KGaA, 69451 Weinheim, 2014

1,4-Addition of $TMSCCl_3$ to Nitroalkenes: Efficient Reaction Conditions and Mechanistic Understanding

Na Wu, Benoit Wahl, Simon Woodward,* and William Lewis[a]

chem_201402394_sm_miscellaneous_information.pdf

1. **General information**
2. **Experimental procedures and characterisation data**
3. **Additional data for NMR and mechanistic experiments**
4. **^{1}H and ^{13}C-NMR spectra for new compounds**
5. **X-ray crystal structures**

1. General information

Tetrahydrofuran (THF) was dried and distilled from sodium/benzophenone. Hexamethyldisilazane (HMDS) and TMSCl were distilled under argon prior to use. Chloroform was dried over CaH_2 and distilled under argon prior to use. Stocks of *n*-BuLi in hexane were assayed by double Gilman titration methods. Toluene was dried over sodium. All other solvents and reagents were used as received from commercial suppliers. Column chromatography was performed using Davisil silica gel 60, and TLC analysis was performed with Merck silica gel 60 F_{254nm}. NMR spectra (^1H, ^{13}C) were recorded with Bruker AV400, DPX400 or AV(III)400 spectrometers. Chemical shift values are reported in ppm, and solvent resonances were used as internal standards ($CHCl_3$: δ = 7.26 ppm for ^1H, δ = 77.16 ppm for ^{13}C). Coupling constants (J) are quoted in Hertz. Carbon NMR multiplicities and connectivities were assigned by using DEPT, HMQC and HMBC experiments. The same instruments were used for ^{19}F and ^{29}Si NMR studies. IR spectra were recorded with a Perkin–Elmer 1600 FTIR spectrometer. HRMS were recorded with a Bruker Apex IV FT-ICRMS instrument (EI). Elemental analyses were performed by using an Exeter Analytical CE-440 instrument. Melting points were determined with a Stuart Scientific SMP3 melting point apparatus. Reduced pressures were controlled by the ‘select pressure mode’ on a vacubrand PC3001 pump.

2. Experimental procedures and characterisation data

Trimethyl(trichloromethyl)silane

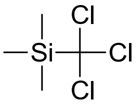

In a flame-dried round bottom flask under argon, HMDS (0.1 mol, 21 mL) was dissolved in 20 mL of dried THF and the solution cooled down to 0 ºC. *n*-BuLi (1.5 M in hexane, 0.1 mol, 65 mL) was then added and the resulting solution was stirred at 0 ºC for 1 hour. In a separate flame-dried Schlenk flask under argon, chloroform (0.15 mol, 12 mL) was added to TMSCl (0.5 mol, 64 mL) at -60 ºC. The solution of LiHMDS was then added to the $CHCl_3$/TMSCl mixture *via* a canula, stirred between -60 ºC and -65 ºC for 30 minutes and allowed to warm up slowly to room temperature overnight. The reaction mixture was quenched by adding water slowly at 0 ºC (**CARE!** a vigorous reaction producing a large amount of HCl ensues). The aqueous layer was extracted with pentane (2 times), the combined organic extracts were dried with anhydrous $MgSO_4$, filtered and concentrated *in vacuo* (**CARE!** the rotavapor water bath should be 40 ºC and the pressure no lower than 50 mbar [38 mm Hg] to prevent product loss by sublimation into the rotavapor; $TMSCCl_3$ should be considered toxic). The crude $TMSCCl_3$, typically slightly suspended within residual THF solvent, was purified by sublimation using a Kugelrohr apparatus connected to a water aspiration pump. The remaining solvent was removed in a first fraction of distillation at about 17 mbar [13 mm Hg]/50 ºC and the $TMSCCl_3$ was then sublimed at about 17 mbar/90 ºC

to yield a white crystalline compound (13.34 g, 70%). M.p. 129-131 ºC; ^{1}H NMR (400 MHz, $CDCl_3$) δ = 0.37 (s, 9 H), ^{13}C NMR (100 MHz, $CDCl_3$) δ = -3.7, 95.6. ^{29}Si NMR (79.5 MHz, $CDCl_3$) δ = 21.2.

General procedure A for the preparation of cyclic nitroolefins 1a-1e using Dauzonne's one-pot procedure:

To a solution of the 2-hydroxybenzaldehyde (2.0 equiv.) and 2-nitroethanol (3.2 equiv.) in *iso*-pentyl acetate (molar concentration of the aldehyde = 0.2 M) under argon, was added di-*n*-butylammonium chloride (1.0 equiv.). The reaction mixture was stirred and refluxed at 142 ºC in a Dean-Stark apparatus overnight. The mixture was then cooled down, concentrated *in vacuo* and purified by flash chromatography on silica gel.

5-Methoxy-3-nitro-2*H*-chromene (1b)

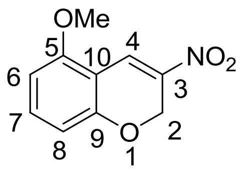

Prepared according to Procedure A from 2-hydroxy-6-methoxybenzaldehyde (1 g, 6.57 mmol); flash chromatography on silica gel (ethyl acetate/*n*-hexane = 1:10, R_f = 0.55 (EtOAc/hexane 1:5)). The product was isolated and further enriched by recrystallisation from *n*-hexane/ethyl acetate to yield orange crystals (0.50 g, 37%): m.p. 92-93 ºC; ^{1}H NMR (400 MHz, $CDCl_3$) δ = 3.89 (s, 3 H, OMe), 5.20 (d, J = 1.0 Hz, 2 H, CH_2), 6.51-6.56 (m, 2 H, Ar), 7.29 (t, J = 8.3 Hz, 1H, C^7H), 8.18 (m, 1H, C^4H); ^{13}C NMR (100 MHz, $CDCl_3$) δ = 56.1, 62.7, 104.3, 108.6, 109.1, 125.4, 134.7, 137.2, 155.9, 158.3; FT-IR (neat) v 3020, 2400, 1645, 1605, 1509, 1481, 1328, 1271, 1216, 1134, 759, 699 cm^{-1}; HRMS (EI^{+}) m/z calcd. for $C_{10}H_9NO_4$ [M^{+}] 207.0532 found 207.0531; Anal. Calc. for $C_{10}H_9NO_4$: C, 57.97; H, 4.38; N, 6.76. Found: C, 57.92; H, 4.38; N, 6.66.

6-Methoxy-3-nitro-2*H*-chromene (1c)

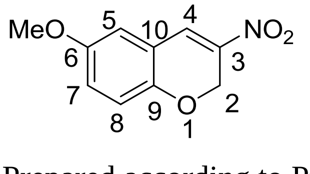

Prepared according to Procedure A from 2-hydroxy-5-methoxybenzaldehyde (1.3 mL, 13.08 mmol); flash chromatography on silica gel (ethyl acetate/*n*-hexane = 1:10, R_f = 0.45 (EtOAc/hexane 1:5)). The product was isolated and further enriched by recrystallisation from *n*-hexane/ethyl acetate to yield dark red crystals (0.71 g, 26%): m.p. 93-94 ºC; ^{1}H NMR (400 MHz, $CDCl_3$) δ = 3.77 (s, 3 H, OMe), 5.16 (s, 2 H, CH_2), 6.74-6.75 (m, 1 H, Ar), 6.82-6.91 (m, 2 H, Ar), 7.71 (s, 1 H, C^4H); ^{13}C NMR (100 MHz, $CDCl_3$) δ = 55.9, 63.0, 114.1, 117.4, 118.9, 120.1, 129.3, 139.9, 149.0,

155.0; FT-IR (neat) *v* 1645, 1574, 1492, 1458, 1316, 1254, 1078, 1033, 952, 937, 839, 711 cm^{-1}; HRMS (EI^{+}) m/z calcd. for $C_{10}H_9NO_4$ [M^{+}] 207.0532 found 207.0538; Anal. Calc. for $C_{10}H_9NO_4$: C, 57.97; H, 4.38; N, 6.76. Found: C, 57.87; H, 4.36; N, 6.64.

7-Methoxy-3-nitro-2*H*-chromene (1d)

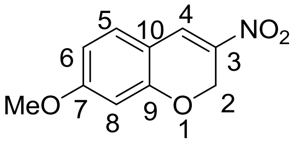

Prepared according to Procedure A from 2-hydroxy-4-methoxybenzaldehyde (1.52 g, 10 mmol); flash chromatography on silica gel (ethyl acetate/*n*-hexane = 1:10, R_f = 0.57 (EtOAc/hexane 1:5)). The product was isolated and further enriched by recrystallisation from *n*-hexane/ethyl acetate to yield bright yellow needles (0.90 g, 43%): m.p. 125-126 ºC; ^{1}H NMR (400 MHz, $CDCl_3$) δ = 3.84 (s, 3 H, OMe), 5.25 (d, *J* = 1.0 Hz, 2 H, CH_2), 6.46 (dd, J_1 = 0.6 Hz, J_2 = 2.4 Hz, 1 H, Ar), 6.58 (dd, J_1 = 2.4 Hz, J_2 = 8.5 Hz, 1 H, Ar), 7.18 (d, *J* = 8.5 Hz, 1 H, Ar), 7.79 (d, *J* = 0.7 Hz, 1 H, C^4H); ^{13}C NMR (100 MHz, $CDCl_3$) δ = 55.8, 63.3, 101.9, 109.8, 111.5, 129.9, 131.9, 136.3, 157.1, 164.9; FT-IR (neat) *v* 1617, 1559, 1495, 1461, 1438, 1332, 1309, 1277, 1254, 872, 811, 716 cm^{-1}; HRMS (EI^{+}) m/z calcd. for $C_{10}H_9NO_4$ [M^{+}] 207.0532 found 207.0525. Anal. Calc. for $C_{10}H_9NO_4$: C, 57.97; H, 4.38; N, 6.76. Found: C, 57.96; H, 4.38; N, 6.65.

6,8-Di-tert-butyl-3-nitro-2*H*-chromene (1e)

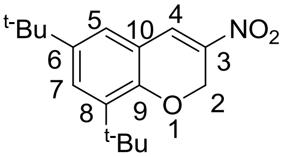

Prepared according to Procedure A from 3,5-di-*tert*-butyl-2-hydroxybenzaldehyde (2.34 g, 10 mmol); flash chromatography on silica gel (ethyl acetate/*n*-hexane = 1:10, R_f = 0.75 (EtOAc/hexane 1:5)). The product was isolated and further enriched by recrystallisation from *n*-hexane/ethyl acetate to yield an orange solid (0.30 g, 10%): m.p. 116-117 ºC; ^{1}H NMR (400 MHz, $CDCl_3$) δ = 1.32 (s, 9 H, $^{t-}$Bu), 1.38 (s, 9 H, $^{t-}$Bu), 5.21 (d, *J* = 0.8 Hz, 2 H, CH_2), 7.13 (d, *J* = 2.4 Hz, 1 H, Ar), 7.42 (d, *J* = 2.4 Hz, 1 H, Ar), 7.81 (s, 1 H, C^4H); ^{13}C NMR (100 MHz, $CDCl_3$) δ = 29.8, 31.5, 34.5, 35.0, 62.3, 119.1, 125.4, 129.1, 130.8, 138.1, 139.0, 145.2, 151.4; FT-IR (neat) *v* 2966, 1652, 1575, 1512, 1331, 1217, 758, 669 cm^{-1}; HRMS (EI^{+}) m/z calcd. for $C_{17}H_{23}NO_3$ [M^{+}] 289.1678 found 289.1671.

Procedure B for Michael additions to cyclic alkenes:

To a solution of the cyclic alkene (1 mmol) and tetrabutylammonium triphenyldifluorosilicate (TBAT) (0.027 g, 5 mol%) in THF (2 mL) under argon, was added dropwise trimethyl(trichloromethyl)silane ($TMSCCl_3$) (0.21 g, 1.1 mmol) in

THF (2 mL) at room temperature and it was stirred overnight. The mixture was concentrated *in vacuo* and then purified by flash chromatograph on silica gel to give the corresponding Michael addition products.

Procedure C for Michael additions to acyclic alkenes:

To a solution of trimethyl(trichloromethyl)silane (TMSCCl_3) (0.21 g, 1.1 mmol) and tetrabutylammonium triphenyldifluorosilicate (TBAT) (0.027 g, 5 mol%) in THF (2 mL) under argon, was added the acyclic alkene (1 mmol) in THF (2 mL) dropwise over a period of 1 hour, at room temperature and it was stirred overnight. The mixture was concentrated *in vacuo* and then purified by flash chromatograph on silica gel to give the corresponding Michael addition products.

3-Nitro-4-(trichloromethyl)chroman (2a)

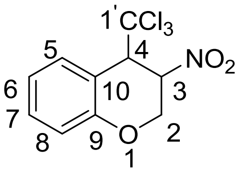

Prepared according to Procedure B from 3-nitro-2*H*-chromene (0.177 g, 1 mmol); silica gel purification (2.5 cm × 25 cm, ethyl acetate/*n*-hexane = 1:20, R_f = 0.59 (EtOAc/hexane 1:5)); yield: 95% (0.279 g, colourless oil); ^{1}H NMR (400 MHz, $CDCl_3$) δ = 4.77-4.82 (m, 1 H, C^2H), 4.90-4.94 (m, 2 H, $C^{2\ and\ 4}H$), 5.55-5.58 (m, 1 H, C^3H), 6.96 (dd, J_1 = 1.0 Hz, J_2 = 8.5 Hz, 1 H, Ar), 7.02 (dt, J_1 = 1.2 Hz, J_2 = 7.8 Hz, 1 H, Ar), 7.34 (dt, J_1 = 1.5 Hz, J_2 = 8.5 Hz, 1 H, Ar), 7.67 (dd, J_1 = 1.4 Hz, J_2 = 7.8 Hz, 1 H, Ar); ^{13}C NMR (100 MHz, $CDCl_3$) δ = 56.3, 63.9, 100.8, 114.7, 117.7, 121.6, 131.2, 132.5, 154.8; FT-IR (neat) *v* 2926, 2854, 1608, 1585, 1560, 1490, 1462, 1359, 1261, 1231, 1125, 795, 758, 609, 443 cm^{-1}; HRMS (EI$^+$) m/z calcd. for $C_{10}H_8Cl_3NO_3$ [M$^+$] 294.9570 found 294.9574.

3-Nitro-4-(trichloromethyl)-5-methoxy-chroman (2b)

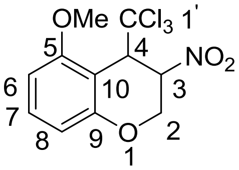

Prepared according to Procedure B from 5-methoxy-3-nitro-2*H*-chromene (0.207 g, 1 mmol); silica gel purification (2.5 cm × 25 cm, ethyl acetate/*n*-hexane = 1:(500, R_f = 0.58 (EtOAc/hexane 1:5)); yield: 72% (0.234 g, yellow oil); ^{1}H NMR (400 MHz, $CDCl_3$) δ = 3.84 (s, 3 H, OMe), 4.69-4.73 (m, 1 H, C^2H), 4.93 (dd, J_1 = 6.0 Hz, J_2 = 13.1 Hz, 1 H, C^2H), 5.38-5.39 (m, 1 H, C^4H), 5.58-5.62 (m, 1 H, C^3H), 6.55-6.61 (m, 2 H, Ar), 7.25-7.29 (m, 1 H, Ar); ^{13}C NMR (100 MHz, $CDCl_3$) δ = 52.0, 55.7, 64.4, 81.4, 100.6, 104.4, 105.2, 110.1, 131.3, 156.3, 158.8; FT-IR (neat) *v* 2942, 1644, 1605, 1559, 1509, 1477, 1359, 1327, 1272, 1251, 1131, 1093, 771, 603 cm^{-1}; HRMS (EI$^+$)

m/z calcd. for $C_{11}H_{10}Cl_3NO_4$ [M^+] 324.9675 found 324.9672.

3-Nitro-4-(trichloromethyl)-6-methoxy-chroman (2c)

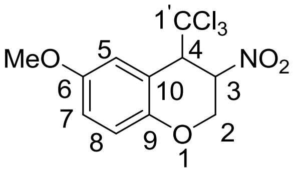

Prepared according to Procedure B from 6-methoxy-3-nitro-2*H*-chromene (0.207 g, 1 mmol); silica gel purification (2.5 cm × 25 cm, ethyl acetate/*n*-hexane = 1:120, R_f = 0.51 (EtOAc/hexane 1:5)); yield: 61% (0.198 g, white solid); m.p. 79-80 ºC; ^{1}H NMR (400 MHz, $CDCl_3$) δ = 3.79 (s, 3 H, OMe), 4.61-4.66 (m, 1 H, C^2H), 4.83-4.89 (m, 2 H, $C^{2\text{ and }4}$H), 5.54-5.57 (m, 1 H, C^3H), 6.88-6.93 (m, 2 H, Ar), 7.22 (d, *J* = 2.3 Hz, 1 H, Ar); ^{13}C NMR (100 MHz, $CDCl_3$) δ = 55.8, 56.4, 81.0, 100.8, 115.6, 116.8, 117.3, 118.3, 149.0, 153.9; FT-IR (neat) *v* 2925, 1542, 1497, 1258, 1205, 1043, 819, 753, 720, 479 cm^{-1}; HRMS (EI$^+$) m/z calcd. for $C_{11}H_{10}Cl_3NO_4$ [M^+] 324.9675 found 324.9671.

3-Nitro-4-(trichloromethyl)-7-methoxy-chroman (2d)

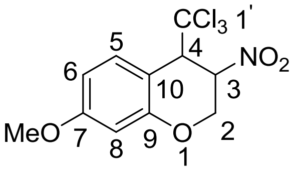

Prepared according to Procedure B from 7-methoxy-3-nitro-2*H*-chromene (0.207 g, 1 mmol); silica gel purification (2.5 cm × 25 cm, ethyl acetate/*n*-hexane = 1:500 then 1:300, R_f = 0.50 (EtOAc/hexane 1:5)); yield: 68% (0.221 g, yellow oil); ^{1}H NMR (400 MHz, $CDCl_3$) δ 3.79 (s, 3 H, OMe), 4.79-4.91 (m, 3 H, $C^{2\text{ and }4}$H), 5.51-5.53 (m, 1 H, C^3H), 6.48 (d, *J* = 2.6 Hz, 1 H, Ar), 6.60 (dd, J_1 = 2.6 Hz, J_2 = 8.7 Hz, 1 H, Ar), 7.55 (d, *J* = 8.7 Hz, 1 H, Ar); ^{13}C NMR (100 MHz, $CDCl_3$) δ 55.5, 55.9, 63.6, 80.5, 101.2, 101.9, 106.5, 108.9, 133.2, 155.8, 161.8; FT-IR (neat) *v* 2956, 1732, 1619, 1559, 1506, 1460, 1331, 1274, 1250, 1167, 1141, 1033, 792, 741, 716 cm^{-1}; HRMS (EI$^+$) m/z calcd. for $C_{11}H_{10}Cl_3NO_4$ [M^+] 324.9675 found 324.9679.

3-Nitro-4-(trichloromethyl)-6,8-di-tert-butyl-chroman (2e)

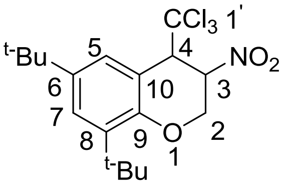

Prepared according to Procedure B from 6,8-di-*tert*-butyl-3-nitro-2*H*-chromene (0.289 g, 1 mmol); silica gel purification (2.5 cm × 25 cm, ethyl acetate/*n*-hexane = 1:500, R_f = 0.77 (EtOAc/hexane 1:5)); yield: 94% (0.383 g, white solid); m.p. 57-58 ºC; ^{1}H NMR (400 MHz, $CDCl_3$) δ = 1.34 (s, 9 H, $^{t-}$Bu), 1.38 (s, 9 H, $^{t-}$Bu), 4.17 (dd, J_1

= 8.3 Hz, J_2 = 11.2 Hz, 1 H, C^2H), 4.93-4.97 (m, 2 H, $C^{2\ and\ 4}H$), 5.65-5.69 (m, 1 H, C^3H), 7.37 (d, J = 2.3 Hz, 1 H, Ar), 7.51 (d, J = 2.3 Hz, 1 H, Ar); ^{13}C NMR (100 MHz, $CDCl_3$) δ = 29.9, 31.6, 34.6, 34.9, 58.2, 66.6, 82.5, 101.9, 117.1, 125.0, 127.9, 138.2, 143.9, 153.5; FT-IR (neat) ν 3020, 2965, 1560, 1480, 1363, 1216, 1052, 770 cm^{-1}; HRMS (EI^+) m/z calcd. for $C_{18}H_{24}Cl_3NO_3$ [M^+] 407.0822 found 407.0833; Anal. Calc. for $C_{18}H_{24}Cl_3NO_3$: C, 52.89; H, 5.92; N, 3.43. Found: C, 53.09; H, 5.92; N, 3.30.

(1’,1’,1’-Trichloro-3’-nitropropan-2’-yl)benzene (2f)

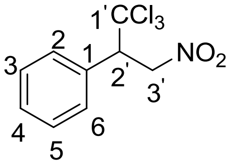

Prepared according to Procedure C from (*E*)-(2-nitrovinyl)benzene (0.149 g, 1 mmol); silica gel purification (2.5 cm × 25 cm, ethyl acetate/*n*-hexane = 1:60, R_f = 0.66 (EtOAc/hexane 1:5)); yield: 58% (0.155 g, yellow solid); m.p. 74-75 ºC; ^1H NMR (400 MHz, $CDCl_3$) δ = 4.67 (dd, J_1 = 4.0 Hz, J_2 = 9.8 Hz, 1 H, $C^{2'}H$), 5.12 (dd, J_1 = 9.8 Hz, J_2 = 13.7 Hz, 1 H, $C^{3'}H$), 5.34 (dd, J_1 = 4.0 Hz, J_2 = 13.7 Hz, 1 H, $C^{3'}H$), 7.40-7.44 (m, 2 H, Ar), 7.50-7.53 (m, 2 H, Ar); ^{13}C NMR (100 MHz, $CDCl_3$) δ = 62.7, 76.7, 99.5, 128.8, 129.8, 130.1, 132.9; FT-IR (neat) ν 1713, 1560, 1377, 1222, 1092, 1040, 921, 825, 745, 700 cm^{-1}; HRMS (EI^+) m/z calcd. for $C_9H_8Cl_3NO_2$ [M^+] 266.9621 found 266.9628.

(1’,1’,1’-Trichloro-3’-nitropropan-2’-yl)-2-methyl-benzene (2g)

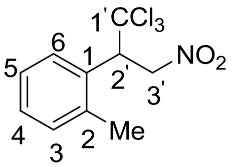

Prepared according to Procedure C from (*E*)-1-methyl-2-(2-nitrovinyl)benzene (0.163 g, 1 mmol); silica gel purification (2.5 cm × 25 cm, ethyl acetate/*n*-hexane = 1:250, R_f = 0.77 (EtOAc/hexane 1:5)); yield: 75% (0.211 g, white solid); m.p. 85-86 ºC; ^1H NMR (400 MHz, $CDCl_3$) δ = 2.58 (s, 3 H, Me), 5.09-5.16 (m, 2 H, $C^{2'\ and\ 3'}H$), 5.30-5.37 (m, 1 H, $C^{3'}H$), 7.27-7.35 (m, 3 H, Ar), 7.64-7.66 (m, 1 H, Ar); ^{13}C NMR (100 MHz, $CDCl_3$) δ = 20.4, 56.8, 77.21, 99.9, 126.5, 126.7, 129.5, 131.4, 131.9, 139.4; FT-IR (neat) ν 2920, 1561, 1552, 1373, 761, 719, 626, 603 cm^{-1}; HRMS (EI^+) m/z calcd. for $C_{10}H_{10}Cl_3NO_2$ [M^+] 280.9777 found 280.9774; Anal. Calc. for $C_{10}H_{10}Cl_3NO_2$: C, 42.51; H, 3.57; N, 4.96. Found: C, 42.60; H, 3.55; N, 4.84.

(1’,1’,1’-Trichloro-3’-nitropropan-2’-yl)-4-methyl-benzene (2h)

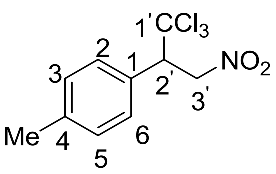

Prepared according to Procedure C from (*E*)-1-methyl-4-(2-nitrovinyl)benzene (0.163 g, 1 mmol); silica gel purification (2.5 cm × 25 cm, ethyl acetate/*n*-hexane = 1:500, R_f = 0.55 (EtOAc/hexane 1:5)); yield: 75% (0.211 g, colourless oil); ^{1}H NMR (400 MHz, $CDCl_3$) δ = 2.37 (s, 3 H, Me), 4.62 (dd, J_1 = 4.0 Hz, J_2 = 10.0 Hz, 1 H, $C^{2'}H$), 5.10 (dd, J_1 = 10.0 Hz, J_2 = 13.6 Hz, 1 H, $C^{3'}H$), 5.32 (dd, J_1 = 4.0 Hz, J_2 = 13.6 Hz, 1 H, $C^{3'}H$), 7.22 (d, J = 8.1 Hz, 2 H, Ar), 7.39 (d, J = 8.1 Hz, 2 H, Ar); ^{13}C NMR (100 MHz, $CDCl_3$) δ = 21.3, 62.5, 76.8, 99.7, 129.5, 129.8, 130.0, 139.9; FT-IR (neat) v 2925, 1560, 1514, 1438, 1374, 1026, 847, 805, 791, 722, 647, 629 cm^{-1}; HRMS (EI$^+$) m/z calcd. for $C_{10}H_{10}Cl_3NO_2$ [M$^+$] 280.9777 found 280.9774.

1-Methoxy-3-(1',1',1'-Trichloro-3'-nitropropan-2'-yl)-benzene (2i)

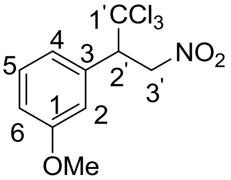

Prepared according to Procedure C from (*E*)-1-methoxy-3-(2-nitrovinyl)benzene (0.179 g, 1 mmol); silica gel purification (2.5 cm × 25 cm, ethyl acetate/*n*-hexane = 1:500 then 1:200, R_f = 0.38 (EtOAc/hexane 1:5)); yield: 52% (0.154 g, colourless oil); ^{1}H NMR (400 MHz, $CDCl_3$) δ = 3.82 (s, 3 H, OMe), 4.63 (dd, J_1 = 3.9 Hz, J_2 = 9.8 Hz, 1 H, $C^{2'}H$), 5.09 (dd, J_1 = 9.8 Hz, J_2 = 13.6 Hz, 1 H, $C^{3'}H$), 5.31 (dd, J_1 = 3.9 Hz, J_2 = 13.6 Hz, 1H, $C^{3'}H$), 6.94-6.97 (m, 1 H, Ar), 7.04 (m, 1 H, Ar), 7.07-7.09 (m, 1 H, Ar), 7.32 (t, J = 8 Hz, 1 H, Ar); ^{13}C NMR (100 MHz, $CDCl_3$) δ = 55.4, 62.7, 76.8, 99.4, 114.7, 116.5, 122.3, 129.8, 134.3, 159.7; FT-IR (neat) v 2926, 1734, 1602, 1587, 1561, 1492, 1456, 1437, 1377, 1294, 1264, 1159, 1051, 922, 878, 810, 779, 697 cm^{-1}; HRMS (EI$^+$) m/z calcd. for $C_{10}H_{10}Cl_3NO_3$ [M$^+$] 296.9726 found 296.9722.

1-Methoxy-4-(1',1',1'-trichloro-3'-nitropropan-2'-yl)benzene (2j)

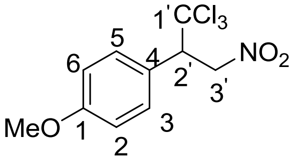

Prepared according to Procedure C from (*E*)-1-methoxy-4-(2-nitrovinyl)benzene (0.179 g, 1 mmol); silica gel purification (2.5 cm × 25 cm, ethyl acetate/*n*-hexane = 1:250 then 1:100 and then 1:50, R_f = 0.48 (EtOAc/hexane 1:5)); yield: 46% (0.136 g, white solid); m.p. 119-120 ºC; ^{1}H NMR (400 MHz, $CDCl_3$) δ 3.82 (s, 3 H, OMe), 4.59 (dd, J_1 = 4.0 Hz, J_2 = 10.1 Hz, 1 H, $C^{2'}H$), 5.07 (dd, J_1 = 10.1 Hz, J_2 = 13.5 Hz, 1 H, $C^{3'}H$), 5.31 (dd, J_1 = 4.0 Hz, J_2 = 13.5 Hz, 1 H, $C^{3'}H$), 6.90-6.94 (m, 2 H, Ar), 7.40-7.43 (m, 2 H, Ar); ^{13}C NMR (100 MHz, $CDCl_3$) δ 55.4, 62.2, 76.8, 100.0, 114.2, 124.6, 131.4, 160.6; FT-IR (neat) v 1610, 1558, 1516, 1377, 1297, 1255, 1182, 1123, 1032, 921, 848, 798, 628 cm^{-1}; HRMS (EI$^+$) m/z calcd. for $C_{10}H_{10}Cl_3NO_3$ [M$^+$] 296.9726 found 296.9715.

(1’,1’,1’-Trichloro-3’-nitropropan-2’-yl)-4-fluoro-benzene (2k)

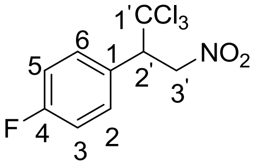

Prepared according to Procedure C from (*E*)-1-fluoro-4-(2-nitrovinyl)benzene (0.167 g, 1 mmol); silica gel purification (2.5 cm × 25 cm, ethyl acetate/*n*-hexane = 1:500, R_f = 0.35 (EtOAc/hexane 1:5)); yield: 44% (0.125 g, colourless oil); ^{1}H NMR (400 MHz, $CDCl_3$) δ = 4.64 (dd, J_1 = 3.9 Hz, J_2 = 10.2 Hz, 1H, $C^{2'}$H), 5.07 (dd, J_1 = 10.2 Hz, J_2 = 13.6 Hz, 1 H, $C^{3'}$H), 5.33 (dd, J_1 = 3.9 Hz, J_2 = 13.6 Hz, 1 H, $C^{3'}$H), 7.09-7.14 (m, 2 H, Ar), 7.47-7.51 (m, 2 H, Ar); ^{13}C NMR (100 MHz, $CDCl_3$) δ = 62.1, 76.7, 99.3, 116.0 (d, J_{CF} = 22 Hz, $C^{3 \text{ and } 5}$), 128.6 (d, J_{CF} = 3 Hz, C^1), 132.0 (d, J_{CF} = 8 Hz, $C^{2 \text{ and } 6}$), 163.5 (d, J_{CF} = 249 Hz, C^4), 162.3, 164.8; ^{19}F NMR (376 MHz, $CDCl_3$) δ -111.0 (m, 1 F); FT-IR (neat) *v* 1895, 1607, 1559, 1512, 1376, 1235, 1165, 843, 801, 718, 623, 522 cm^{-1}; HRMS (EI$^+$) m/z calcd. for $C_9H_7Cl_3FNO_2$ [M$^+$] 284.9526 found 284.9520.

(1’,1’,1’-Trichloro-3’-nitropropan-2’-yl)-4-methyl-benzoate (2l)

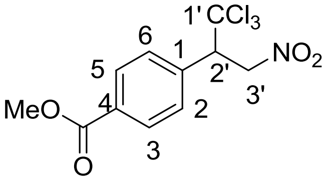

Prepared according to Procedure C from (*E*)-1-methyl-4-(2-nitrovinyl)benzoate (0.104 g, 0.5 mmol); silica gel purification (2.5 cm × 25 cm, ethyl acetate/*n*-hexane = 1:4, R_f = 0.34 (EtOAc/hexane 1:5)); yield: 37% (0.060 g, colourless oil); ^{1}H NMR (400 MHz, $CDCl_3$) δ = 3.93 (s, 3 H), 4.72 (dd, J_1 = 3.9 Hz, J_2 = 10.0 Hz, 1 H, $C^{2'}$H), 5.12 (dd, J_1 = 10.0 Hz, J_2 = 13.8 Hz, 1 H, $C^{3'}$H), 5.35 (dd, J_1 = 3.9 Hz, J_2 = 13.8 Hz, 1 H, $C^{3'}$H), 7.58-7.61 (m, 2 H), 8.07-8.09 (m, 2 H); ^{13}C NMR (100 MHz, $CDCl_3$) δ = 52.5, 62.5, 76.5, 98.7, 130.0, 130.3, 131.6, 137.6, 166.4; FT-IR (neat) *v* 2924, 1724, 1561, 1436, 1376, 1284, 1191, 1114, 807, 754, 703, 622; HRMS (EI$^+$) m/z calcd. for $C_{11}H_{10}Cl_3NO_4$ [M$^+$] 324.9675 found 324.9677.

(1’,1’,1’-Trichloro-2’-methyl-3’-nitropropan-2-yl)benzene (2m)

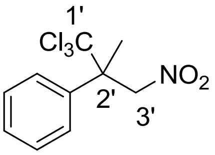

Prepared according to Procedure C from (*E*)-(1-nitroprop-1-en-2-yl)benzene (0.163 g, 1 mmol); silica gel purification (2.5 cm × 25 cm, ethyl acetate/*n*-hexane = 1:300 then 1:75, R_f = 0.65 (EtOAc/hexane 1:5)); yield: 40% (0.112 g, colourless oil); ^{1}H NMR (400 MHz, $CDCl_3$) δ 2.19 (s, 3 H, Me), 5.09 (d, *J* = 12.8 Hz, 1 H, $C^{3'}$H), 5.70 (d, *J* = 12.8 Hz, 1 H, $C^{3'}$H), 7.38-7.41 (m, 3 H, Ar), 7.63-7.68 (m, 2 H, Ar); ^{13}C NMR (100 MHz, $CDCl_3$) δ 22.5, 58.4, 80.5, 106.0, 127.9, 129.0, 129.7, 134.5; FT-IR (neat) *v*

2923, 1558, 1513, 1446, 1386, 1340, 795, 698 cm^{-1}; HRMS (EI$^+$) m/z calcd. for $C_{10}H_{10}Cl_3NO_2$ [M$^+$] 280.9777 found 280.9766.

(4,4,4-Trichloro-3-(nitromethyl)butyl)benzene (2n)

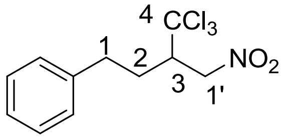

Prepared according to Procedure C from (*E*)-(4-nitrobut-3-enyl)benzene (0.177 g, 1 mmol); silica gel purification (2.5 cm × 25 cm, ethyl acetate/*n*-hexane = 1:500, R_f = 0.72 (EtOAc/hexane 1:5)); yield: 58% (0.171 g, colourless oil); ^{1}H NMR (400 MHz, $CDCl_3$) δ = 1.90-1.99 (m, 1 H, C^{2}H), 2.47-2.55 (m, 1 H, C^{2}H), 2.76-2.91 (m, 2 H, C^{1}H), 3.52-3.58 (m, 1 H, C^{3}H), 4.51 (dd, J_1 = 5.2 Hz, J_2 = 14.5 Hz, 1 H, C$^{1'}$H), 4.99 (dd, J_1 = 5.2 Hz, J_2 = 14.5 Hz, 1 H, C$^{1'}$H), 7.23-7.28 (m, 3 H, Ar), 7.33-7.37 (m, 2 H, Ar); ^{13}C NMR (100 MHz, $CDCl_3$) δ = 33.2, 33.6, 56.9, 76.7, 101.6, 126.8, 128.5, 128.9, 139.8; FT-IR (neat) *v* 3029, 2928, 1559, 1497, 1454, 1427, 1378, 803, 776, 747, 699, 417 cm^{-1}; HRMS (EI$^+$) m/z calcd. for $C_{11}H_{12}Cl_3NO_2$ [M$^+$] 294.9934 found 294.9933.

1,1,1-Trichloro-2-(nitromethyl)hexane (2o)

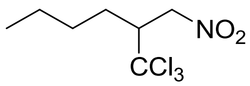

Prepared according to Procedure C from (*E*)-1-nitrohex-1-ene (0.129 g, 1 mmol); silica gel purification (2.5 cm × 25 cm, ethyl acetate/*n*-hexane = 1:250, R_f = 0.76 (EtOAc/hexane 1:5)); yield: 80% (0.199 g, yellow oil); ^{1}H NMR (400 MHz, $CDCl_3$) δ = 0.93 (t, *J* = 7.2 Hz, 3 H), 1.34-1.50 (m, 4 H), 1.52-1.63 (m, 1 H), 2.10-2.18 (m, 1 H), 3.43-3.49 (m, 1 H), 4.42 (dd, J_1 = 5.0 Hz, J_2 = 14.6 Hz, 1 H), 4.91 (dd, J_1 = 5.0 Hz, J_2 = 14.6 Hz, 1 H); ^{13}C NMR (100 MHz, $CDCl_3$) δ = 13.8, 22.5, 29.3, 31.0, 57.4, 76.6, 101.9; FT-IR (neat) *v* 2961, 2932, 2874, 1562, 1378, 920, 804, 767, 619 cm^{-1}; HRMS (EI$^+$) m/z calcd. for $C_7H_{12}Cl_3NO_2$ [M-H_3O^+] 227.9755 found 227.9753.

(1,1,1-Trichloro-3-nitropropan-2-yl)cyclohexane (2p)

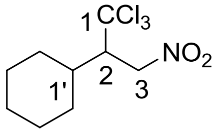

Prepared according to Procedure C from (*E*)-(2-nitrovinyl)cyclohexane (0.155 g, 1 mmol), silica gel purification (2.5 cm × 25 cm, ethyl acetate/*n*-hexane = 1:500, R_f = 0.67 (EtOAc/hexane 1:5)); yield: 61% (0.167 g, colourless oil); ^{1}H NMR (400 MHz, $CDCl_3$) δ = 1.01-1.44 (m, 5 H, CH_2 of Cy), 1.69-1.73 (m, 1 H, CH_2 of Cy), 1.78-1.83 (m, 3 H, CH_2 of Cy), 2.05-2.08 (m, 1 H, CH_2 of Cy), 2.31 (dq, J_1 = 2.5 Hz, J_2 = 12.2 Hz, 1 H, C$^{1'}$H), 3.46-3.49 (m, 1 H, C^{2}H), 4.65 (dd, J_1 = 5.8 Hz, J_2 = 15.0 Hz, 1 H, C^{3}H), 4.87 (dd, J_1 = 4.6 Hz, J_2 = 15.0 Hz, 1 H, C^{3}H); ^{13}C NMR (100 MHz, $CDCl_3$) δ

= 26.0, 26.4, 26.9, 28.3, 33.5, 40.1, 61.7, 73.7, 101.8; FT-IR (neat) *v* 2931, 2856, 1561, 1450, 1429, 1374, 811, 760, 601 cm^{-1}; HRMS (EI^+) m/z calcd. for $C_9H_{14}Cl_3NO_2$ [$M-OH^-$] 256.0057 found 256.0064.

2-nitro-1-(trichloromethyl)-1,2,3,4-tetrahydronaphthalene (2q)

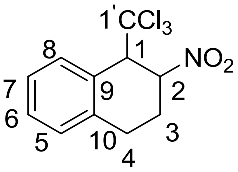

Prepared according to Procedure B from 3-nitro-1,2-dihydronaphthalene (62 mg, 0.354 mmol); silica gel purification (2.5 cm × 25 cm, from pure *n*-pentane to ethyl acetate/*n*-pentane = 1:9, R_f = 0.65 (EtOAc/hexane 1:19)); yield: 75% (0.078 g, slightly yellow solid); ^1H NMR (400 MHz, $CDCl_3$) δ = 1.93-2.14 (m, 1H, C^4H), 2.75-2.95 (m, 2H, $C^{3\text{ and }4}H$), 2.97-3.17 (m, 1H, C^3H), 5.00 (d, 1H, J = 4.2 Hz, C^1H), 5.46 (ddd, J_1 = 4.2 Hz, J_2 = 5.6 Hz, J_3 = 10.1 Hz, 1H, C^2H), 7.22 (d, J = 7.4 Hz, 1H, Ar), 7.25-7.38 (m, 2H, Ar), 7.73 (d, J =7.4 Hz, 1 H, Ar); ^{13}C NMR (100 MHz, $CDCl_3$) δ = 27.3, 30.3, 61.0, 85.7, 102.9, 126.3, 128.7, 129.1, 129.2, 133.4, 139.5; FT-IR ($CHCl_3$) *v* 3009, 2962, 1558, 1523, 1454, 1425, 1372, 1341, 1288, 1239, 1118, 1000, 929, 858, 634 cm^{-1}; HRMS (EI^+) m/z calcd. for $C_{11}H_{10}Cl_3NNaO_2$ $[M+Na]^+$ 315.9669 found 315.9660.

6-(Trichloromethyl)-3a,4,5,6-tetrahydro-3*H*-cyclopenta[c]isoxazole (2r)

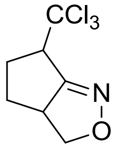

Prepared according to Procedure C (but carried out in air) from 1-nitrohexa-1,5-diene (0.127 g, 1 mmol); silica gel purification (2.5 cm × 25 cm, ethyl acetate/*n*-hexane = 1:20 then 1:5, R_f = 0.25 (EtOAc/hexane 1:5)); yield: 64% (0.135 g, colourless low melting solid); ^1H NMR (400 MHz, $CDCl_3$) δ 1.44-1.54 ($CH_{2\alpha}$CHC=), 2.16-2.23 ($CH_{2\beta}$CHC=), 2.40-2.51 (m, 1 H, $CH_{2\alpha}CHCCl_3$), 2.65-2.73 (m, 1 H, $CH_{2\beta}CHCCl_3$), 3.87-3.96 (2 x CH and $OCH_{2\alpha}$), 4.54-4.63 (m, 1 H, $OCH_{2\beta}$); ^{13}C NMR (100 MHz, $CDCl_3$) δ 27.9, 34.7, 54.6, 56.3, 75.7, 99.4, 167.6; FT-IR (neat) *v* 2930, 1721, 1620, 1459, 1346, 1282, 903, 868, 821, 766.

6-(Dichloromethylene)-3a,4,5,6-tetrahydro-3*H*-cyclopenta[c]isoxazole (2r')

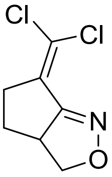

Attempted purification of **2r** by drying ($MgSO_4$) and recrystallization from ethyl

acetate and *n*-hexane overnight at room temperature to provide needle shaped beige crystals, contaminated with traces of **2r** starting material: m.p. (**2r'**) 75-76 ºC ; ^{1}H NMR (400 MHz, $CDCl_3$) δ = 1.62-1.72 (m, 1 H, $CH_{2\alpha}$CH), 2.14-2.21 (m, 1 H, $CH_{2\beta}$CH), 2.99 (ddd, J_1 = 16.1 Hz, J_2 = 12.1 Hz, J_3 = 8.1 Hz, 1 H, $CH_{2\alpha}$C=), 3.06 (dd, J_1 = 16.1 Hz, J_2 = 8.1 Hz, 1 H, $CH_{2\beta}$C=), 3.83-3.97 (m, 2 H, CH and $OCH_{2\alpha}$), 4.57-4.66 (m, 1 H, $OCH_{2\beta}$); ^{13}C NMR (100 MHz, $CDCl_3$) δ = 27.1, 39.0, 56.0, 75.6, 121.4, 126.7, 166.5; FT-IR (neat) *v* 2939, 2875, 1621, 960, 906, 891, 866; HRMS (EI^+) m/z calcd. for $C_7H_7Cl_2NO$ [M^+] 190.9905 found 190.9908.

1,1-Dichloro-1α-nitro-tetrahydrocyclopropa[c]chromene (9)

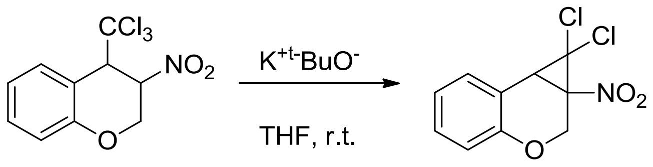

To a solution of 3-nitro-4-(trichloromethyl)chroman (0.142 g, 0.48 mmol) in THF (5 mL) was added potassium *tert*-butoxide (0.081 g, 0.72 mmol) at room temperature and the reaction mixture was stirred for 30 min (until TLC showed complete consumption of the starting material). The mixture was concentrated *in vacuo* and purified by flash chromatograph on silica gel (2.5 cm × 25 cm, ethyl acetate/*n*-hexane = 1:100, R_f (EtOAc/hexane 1:5)) = 0.44)) to yield a white solid (0.0656 g, 53%): m.p. 100-101 ºC; ^{1}H NMR (400 MHz, $CDCl_3$) δ = 4.29 (dd, J_1 = 2.7 Hz, J_2 = 13.0 Hz, 1 H), 5.05 (dd, J_1 = 2.2 Hz, J_2 = 13.0 Hz, 1 H), 5.76 (t, J = 2.4 Hz, 1 H), 6.84 (dd, J_1 = 1.1 Hz, J_2 = 8.3 Hz, 1 H), 6.95 (dt, J_1 = 1.2 Hz, J_2 = 7.7 Hz, 1 H), 7.18-7.23 (m, 1 H), 8.14 (dd, J_1 = 1.4 Hz, J_2 = 8.3 Hz, 1 H); ^{13}C NMR (100 MHz, $CDCl_3$) δ = 66.6, 81.3, 116.4, 117.7, 121.8, 122.7, 124.4, 128.6, 131.4, 153.2; FT-IR (neat) *v* 2924, 2854, 1732, 1565, 1460, 1359, 1249, 1107, 960, 855, 818, 753 cm^{-1}; HRMS (EI^+) m/z calcd. for $C_{10}H_7Cl_2NO_3^+$ [M^+] 258.9803 found 258.9811.

(1,1-Dichloro-3-nitroprop-1-en-2-yl)benzene (10)

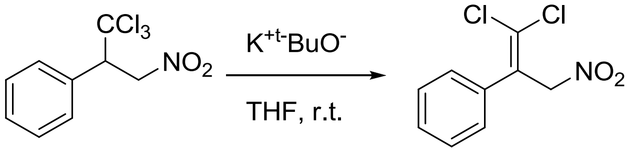

To a solution of (1',1',1'-Trichloro-3'-nitropropan-2'-yl)benzene (0.050 g, 0.187 mmol) in THF (2 mL) was added potassium *tert*-butoxide (0.063 g, 0.561 mmol) at room temperature and the reaction mixture was stirred for 30 min (until TLC showed complete conversion of the starting material). The mixture was concentrated *in vacuo* and purified by flash chromatograph on silica gel (2.5 cm × 25 cm, ethyl acetate/*n*-hexane = 1:100, R_f (EtOAc/hexane 1:5)) = 0.47)) to yield a colourless oil (0.020 g, 47%): ^{1}H NMR (400 MHz, $CDCl_3$) δ = 5.42 (s, 2 H), 7.30-7.32 (m, 2 H), 7.39-7.45 (m, 3 H); ^{13}C NMR (100 MHz, $CDCl_3$) δ = 78.3, 128.5, 129.1, 129.2, 129.5, 129.6, 135.9; FT-IR (neat) *v* 3011, 2927, 2856, 1560, 1522, 1241, 929, 660; HRMS

(EI$^+$) m/z calcd. for $C_9H_7Cl_2NO_2^+$ [M^+] 230.9854 found 230.9857.

3. Additional data for NMR and mechanistic experiments

Reference spectra

^{13}C NMR tetrachloroethylene

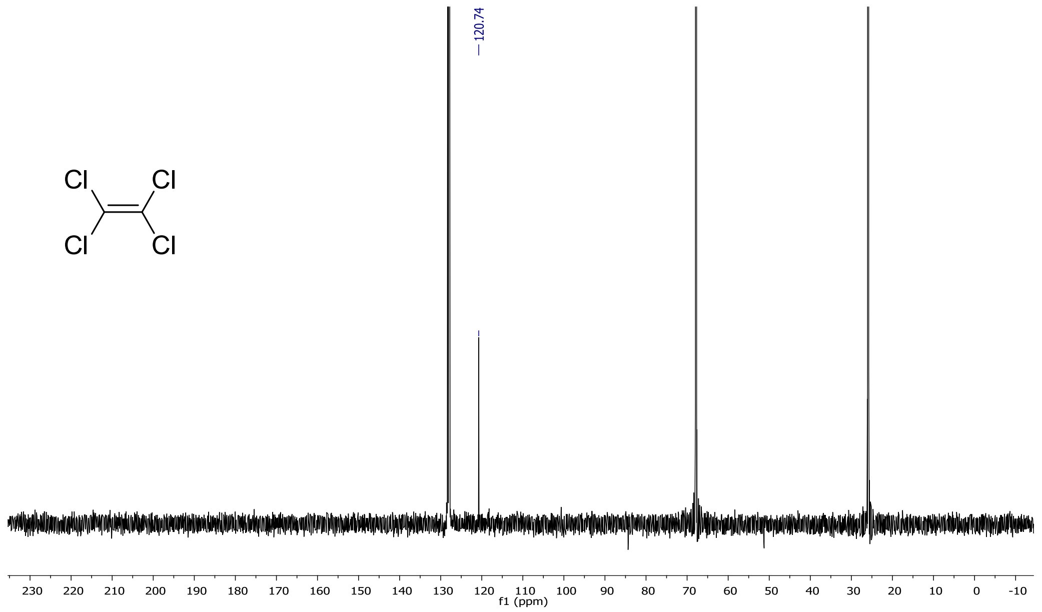

^{13}C NMR of alkene **1a**

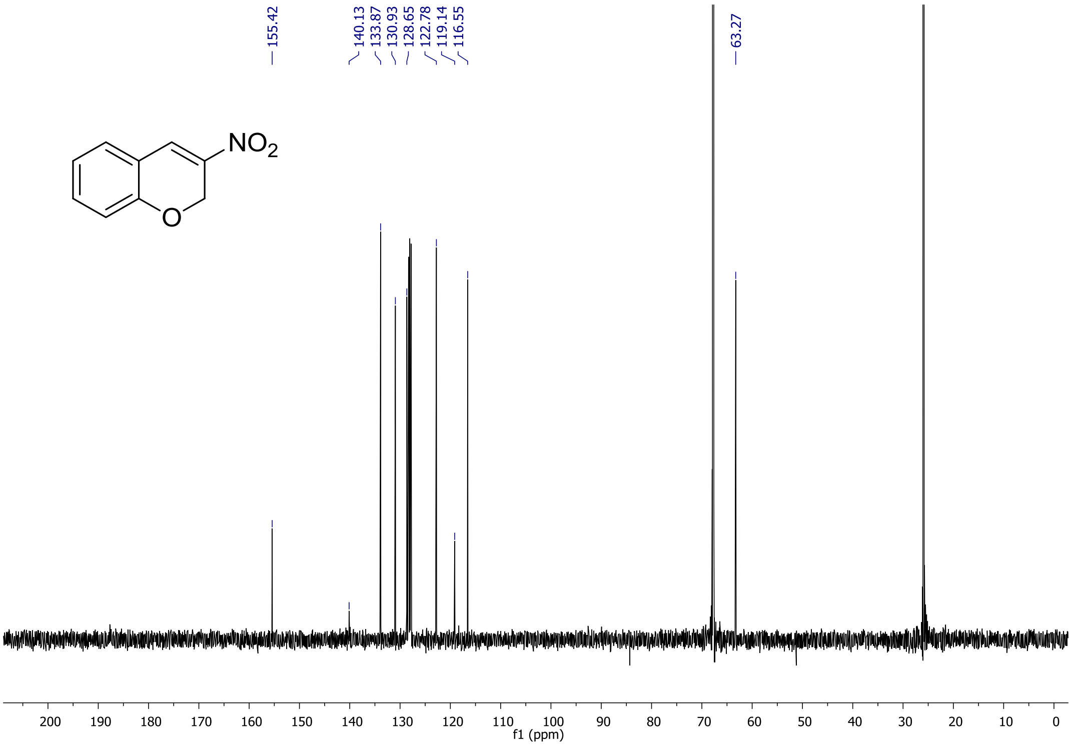

^{13}C NMR of the expected product of addition **2a**

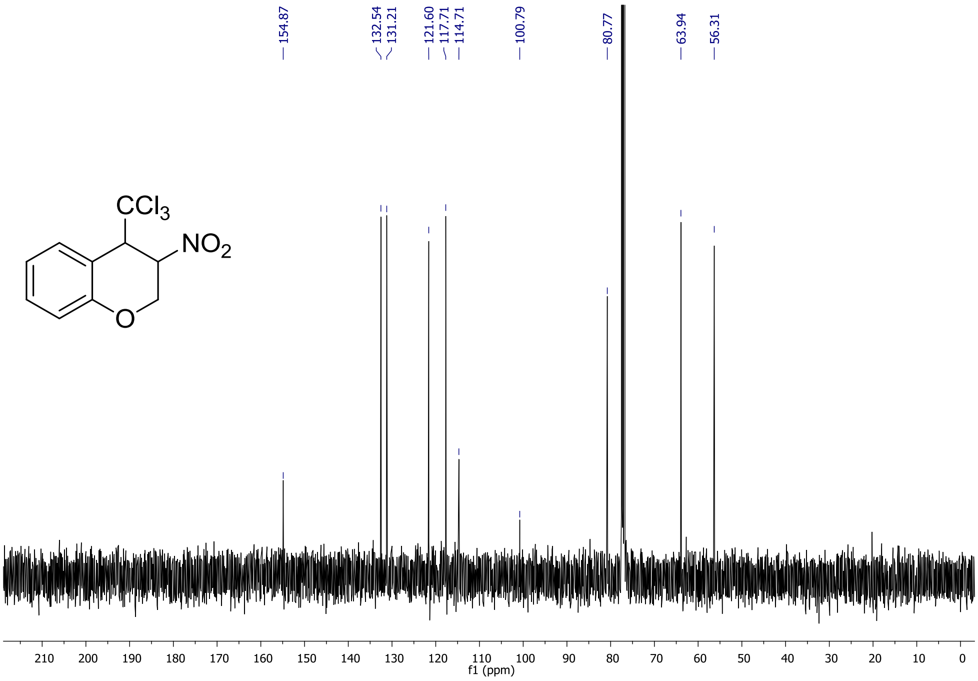

Graphical summary of the two procedures carried out (Alkene last vs. Alkene first) are given below.

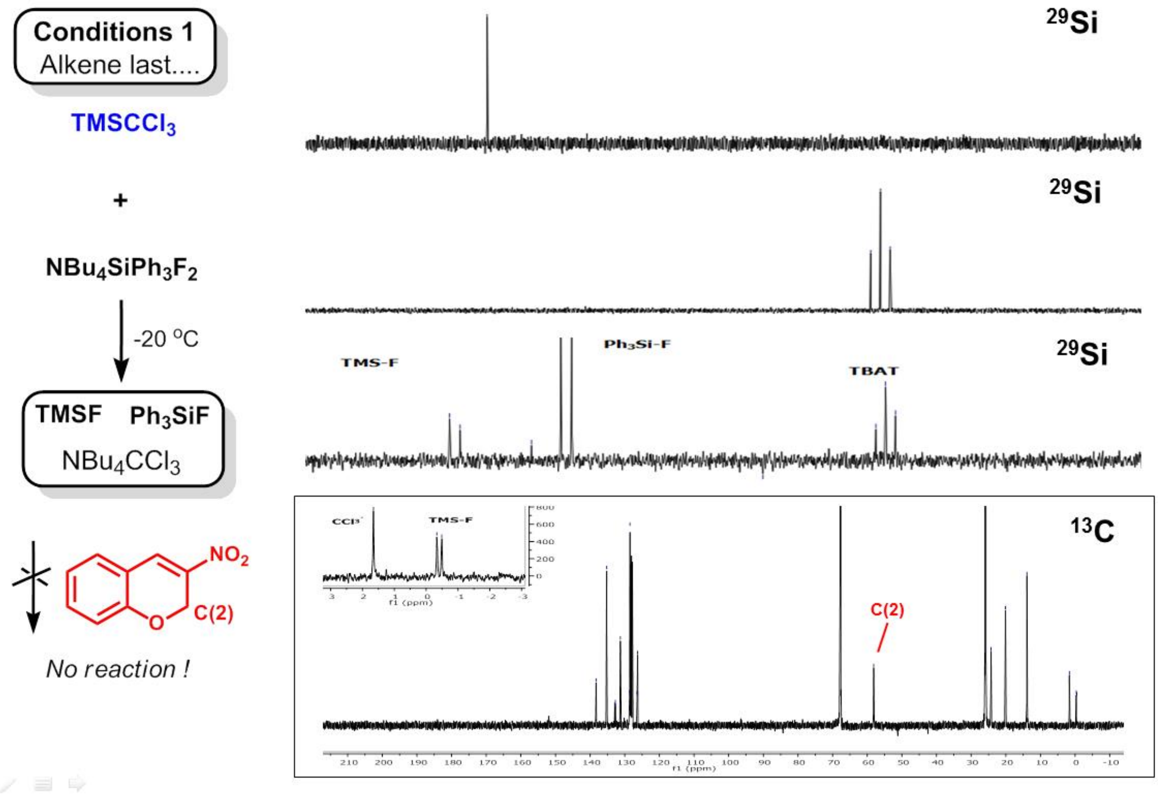

^{19}F NMR after addition of TBAT to $TMSCCl_3$ at -20 ºC (Conditions 1: alkene last)

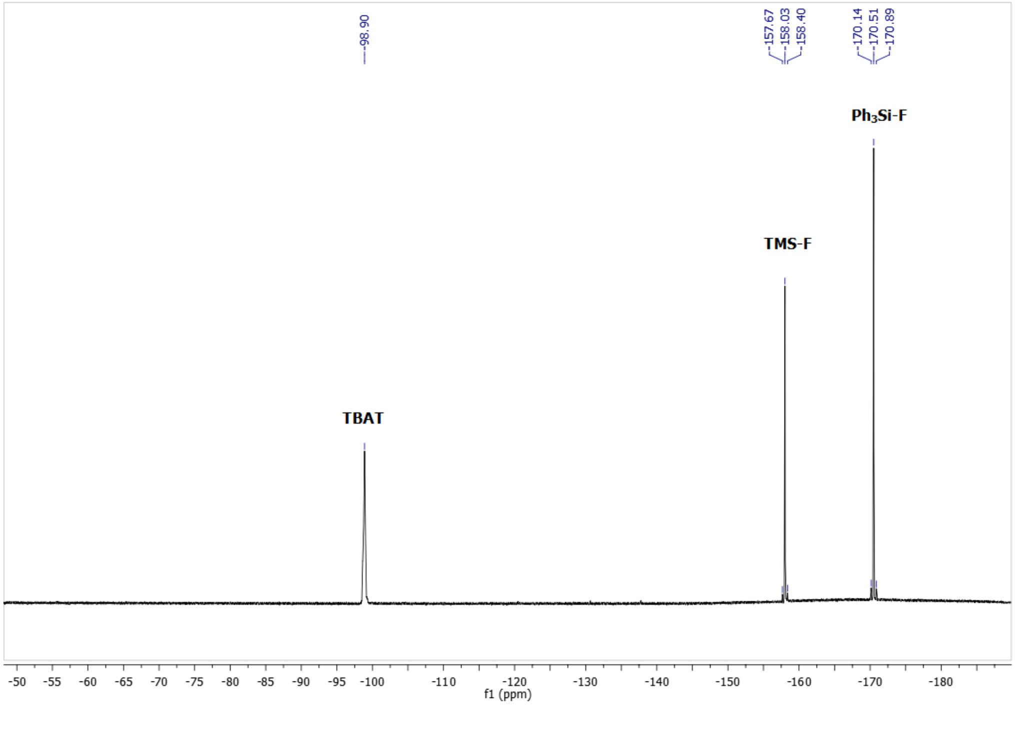

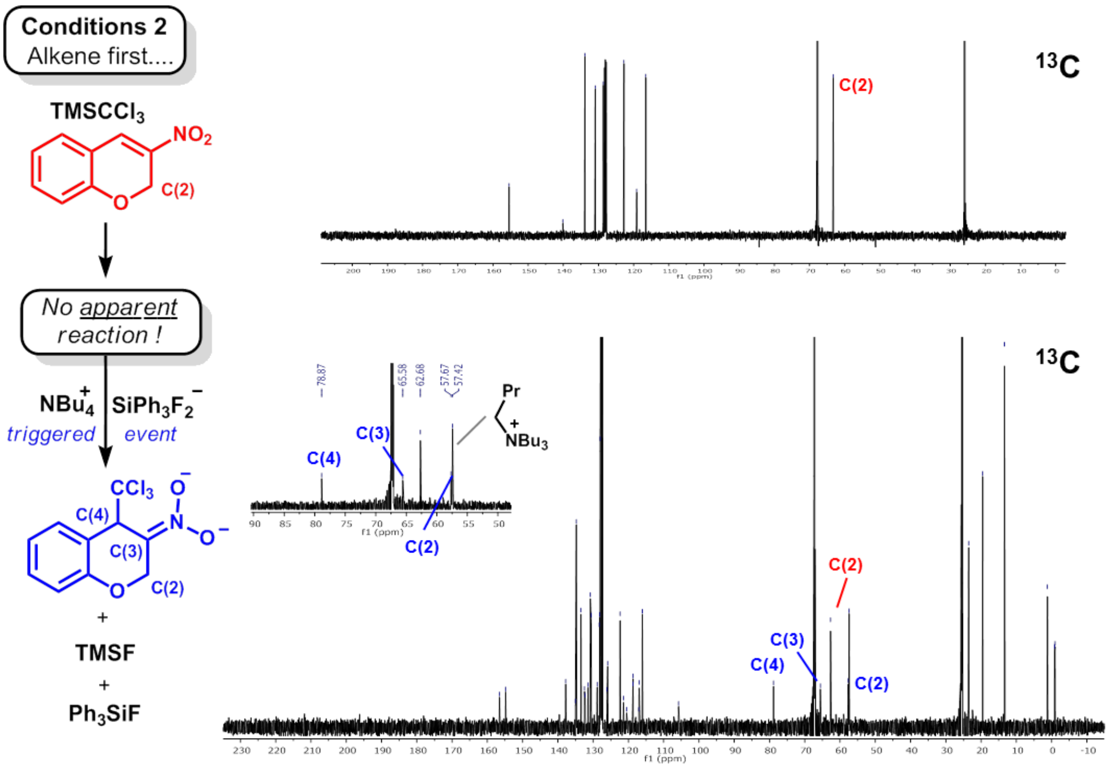

Conditions 2
Alkene first....
TMSCCl3
NO2
C(2)
No apparent reaction !
NBu4
SiPh3F2
triggered
event
CCl3
C(4)
C(3)
C(2)
+
TMSF
+
Ph3SiF
13C
C(2)
13C
78.87
65.58
62.68
57.67
57.42
Pr
NBu3
C(4)
C(3)
C(2)
C(3)
C(4)
C(2)
C(2)
f1 (ppm)

Runs with Chiral Catalysts

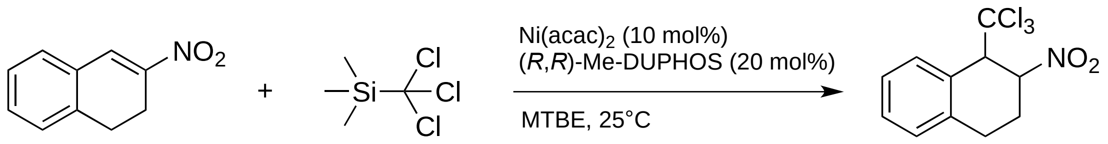

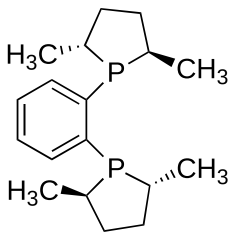

(*R*,*R*)-Me-DUPHOS

Reaction time	enantiomeric excess
15 min	15%
70 min	9%
200 min	4%
270 min	3%
20h	< 1%

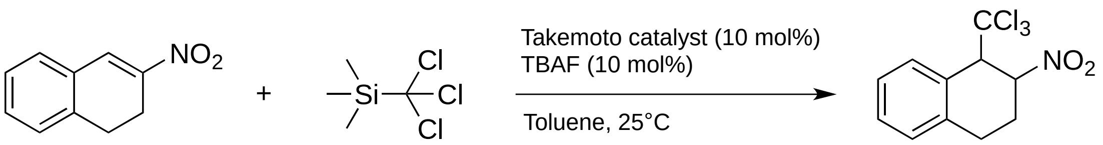

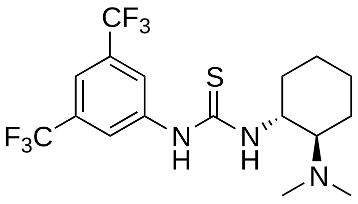

Takemoto catalyst

Reaction time	enantiomeric excess
60 min	26%
120 min	< 1%

4. ^{1}H and ^{13}C-NMR spectra for new compounds

Trimethyl(trichloromethyl)silane

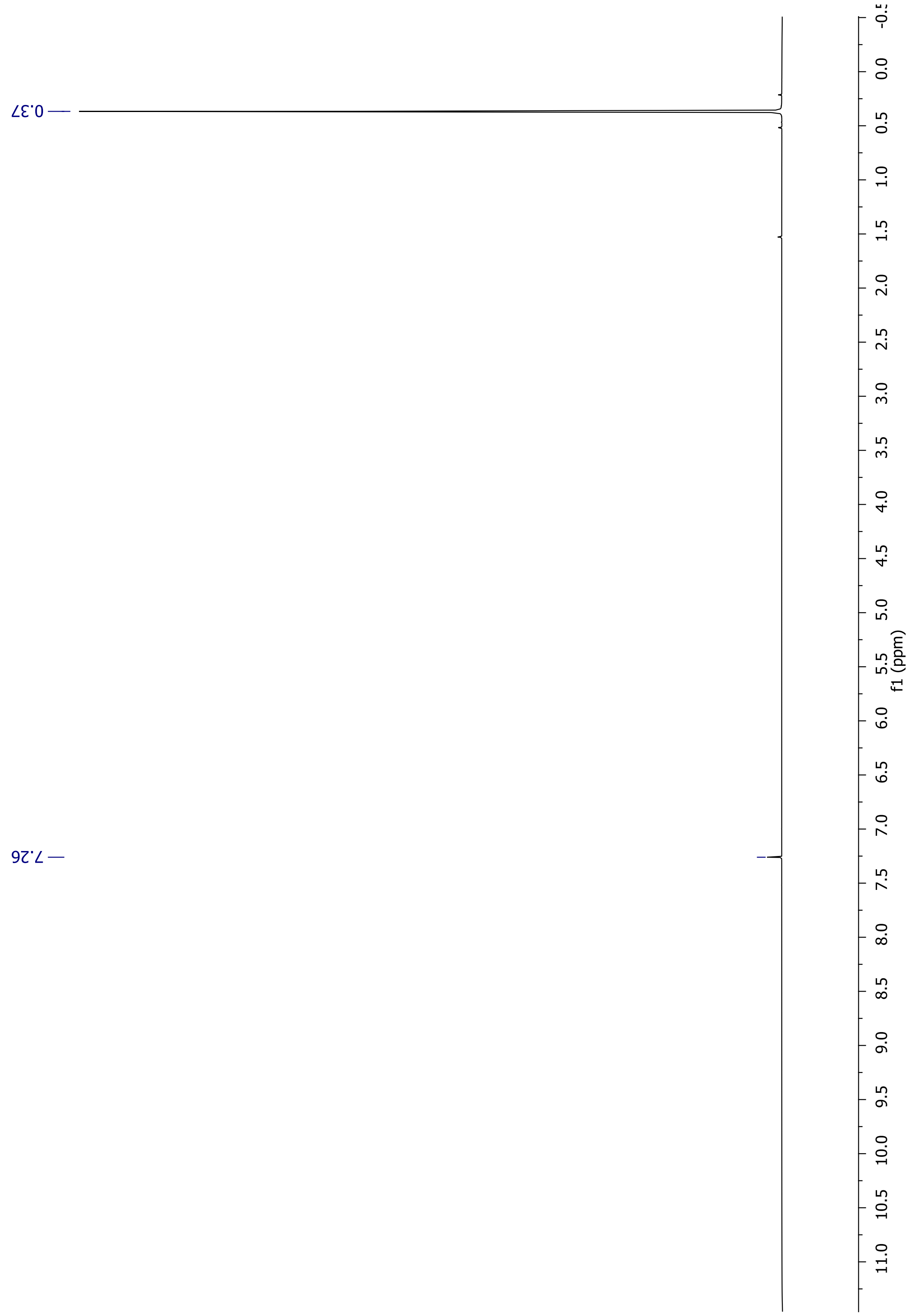

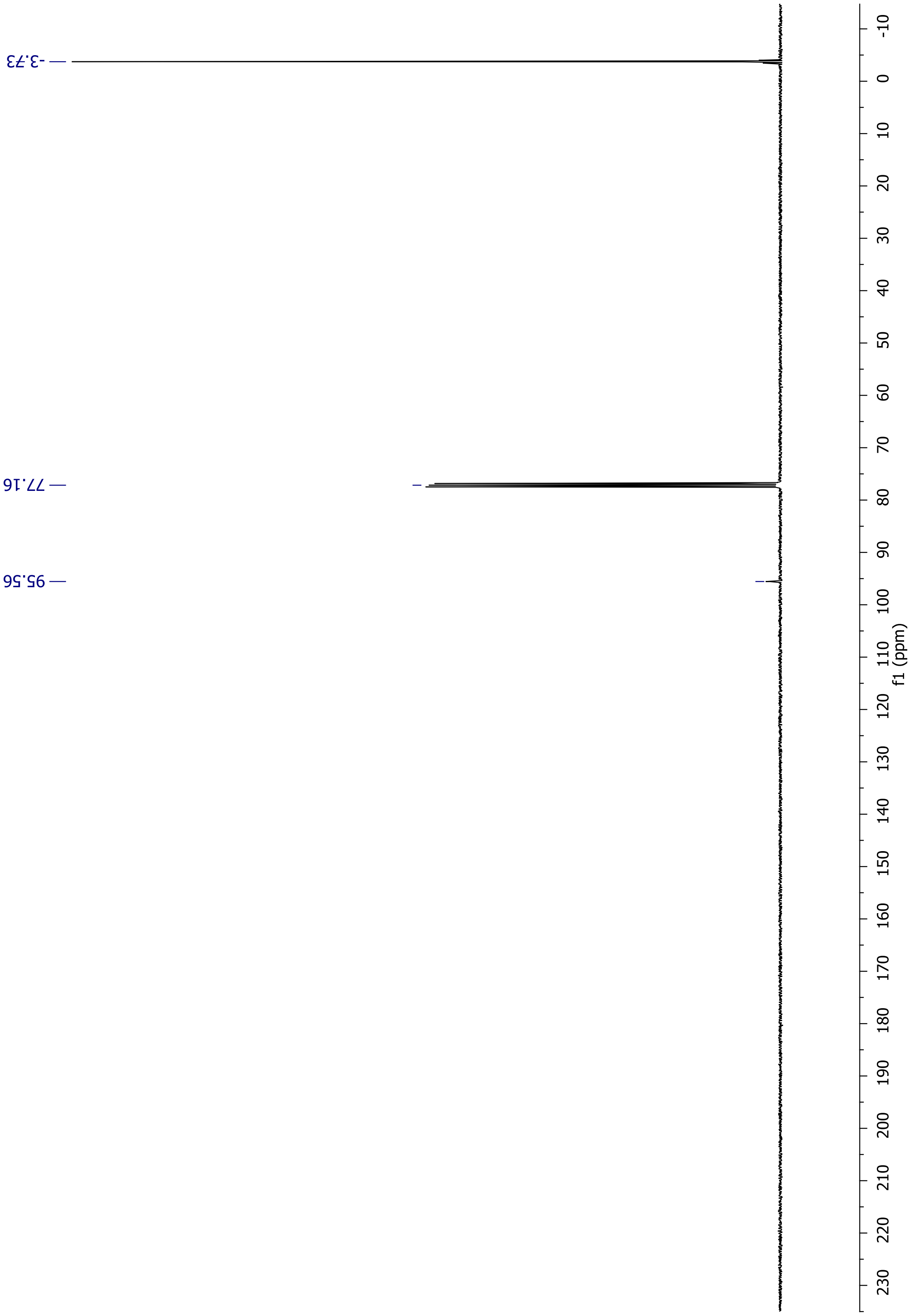
-3.73
77.16
95.56
f1 (ppm)
-10
0
10
20
30
40
50
60
70
80
90
100
110
120
130
140
150
160
170
180
190
200
210
220
230

^{29}Si

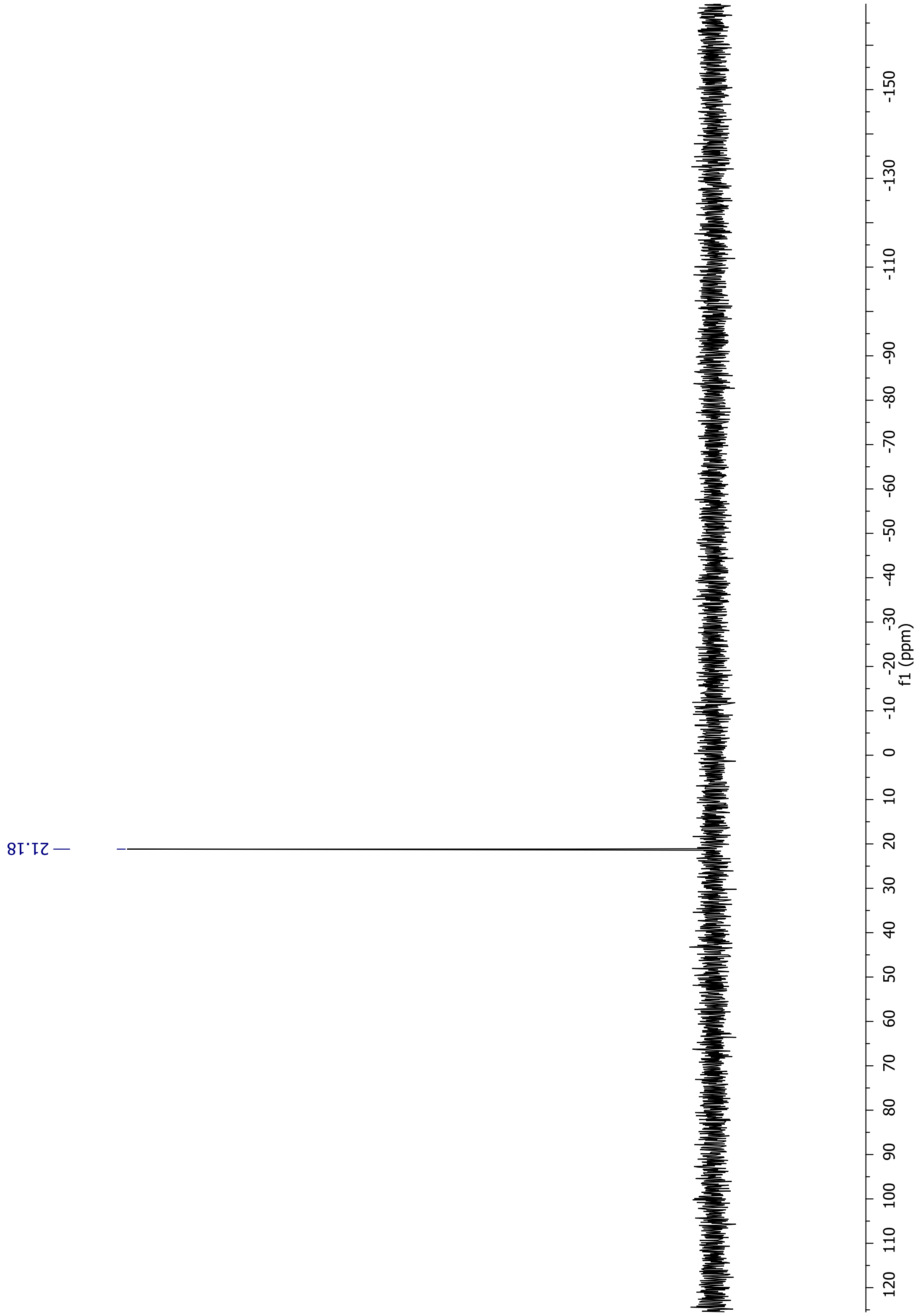

1b

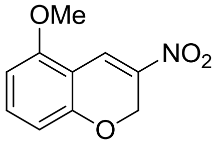
OMe
NO2
O

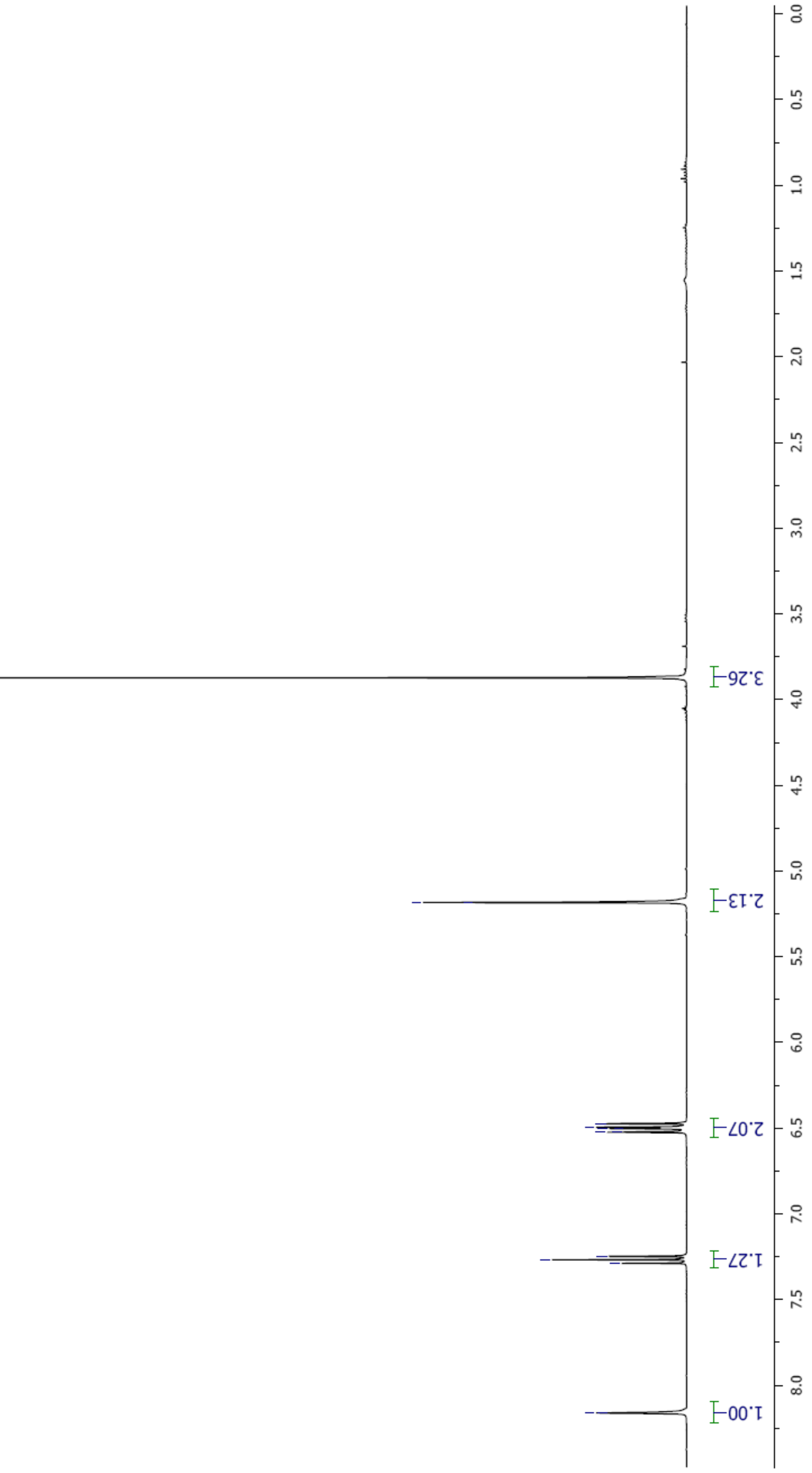
3.8730
5.1830
5.1854
6.4731
6.4743
6.4953
6.5014
6.5032
6.5221
6.5239
7.2470
7.2494
7.2678
7.2886
8.1613
8.1632
3.26
2.13
2.07
1.27
1.00
0.0
0.5
1.0
1.5
2.0
2.5
3.0
3.5
4.0
4.5
5.0
5.5
6.0
6.5
7.0
7.5
8.0

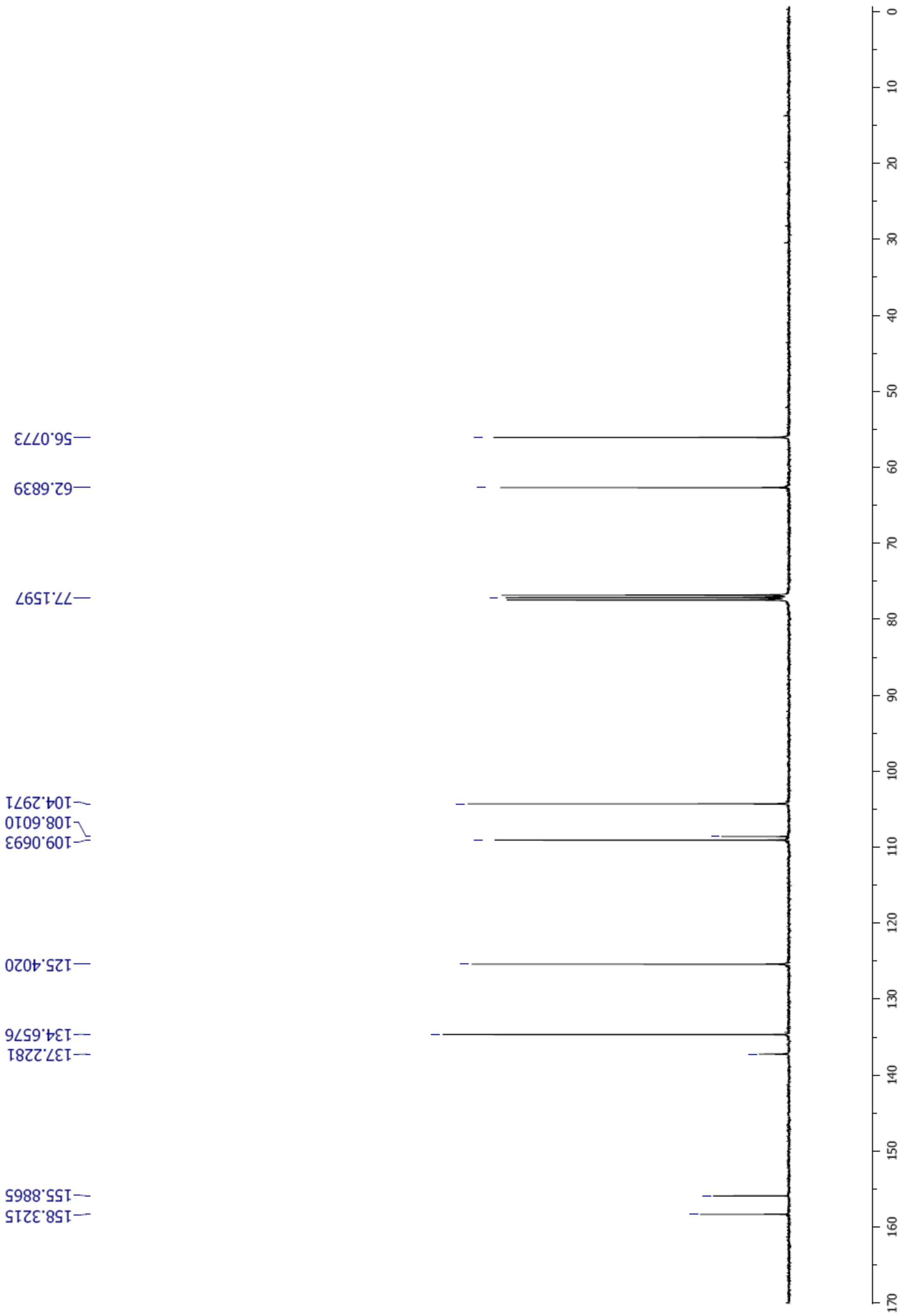
56.0773
62.6839
77.1597
104.2971
108.6010
109.0693
125.4020
134.6576
137.2281
155.8865
158.3215

1c

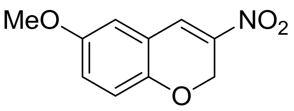
MeO
NO2
O

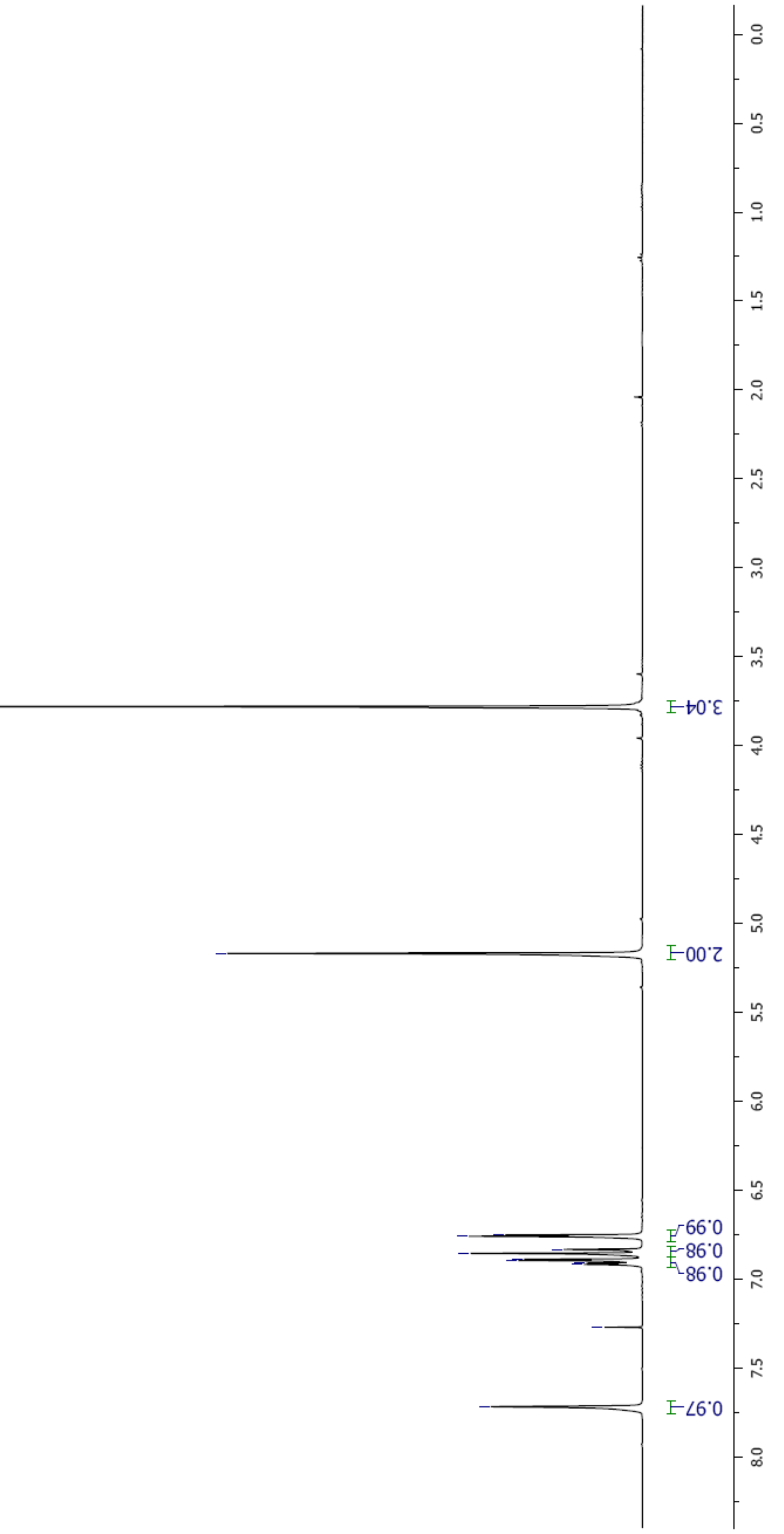
3.7785
5.1668
6.7495
6.7567
6.8306
6.8528
6.8854
6.8926
6.9076
6.9149
7.2681
7.7143
3.04
2.00
0.99
0.98
0.98
0.97
0.0
0.5
1.0
1.5
2.0
2.5
3.0
3.5
4.0
4.5
5.0
5.5
6.0
6.5
7.0
7.5
8.0

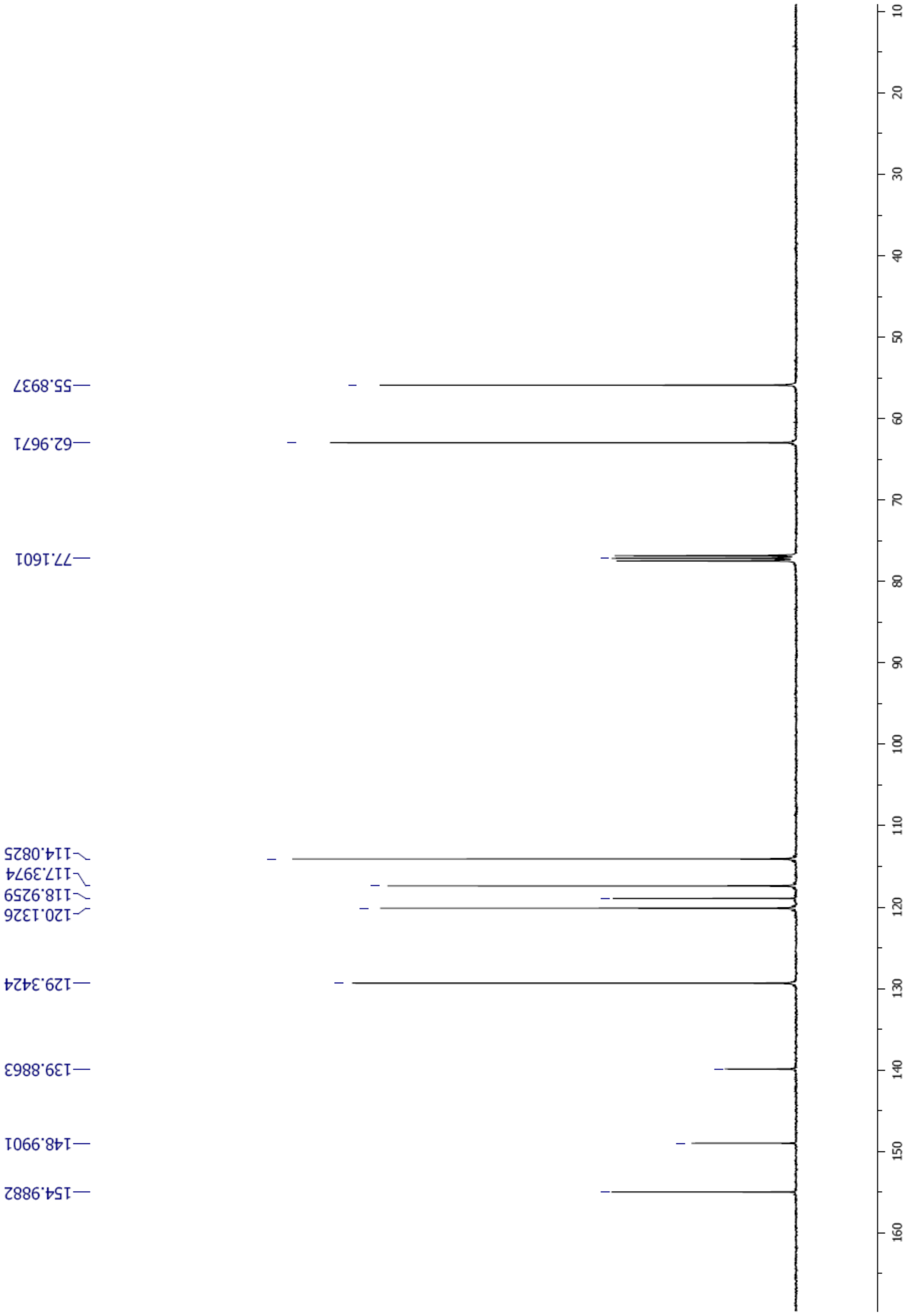

55.8937
62.9671
77.1601
114.0825
117.3974
118.9259
120.1326
129.3424
139.8863
148.9901
154.9882

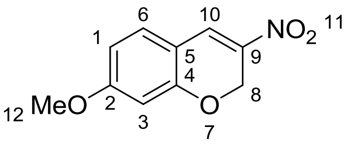
6
10
1
NO2
11
5
4
9
12 MeO
2
8
3
O
7

1d

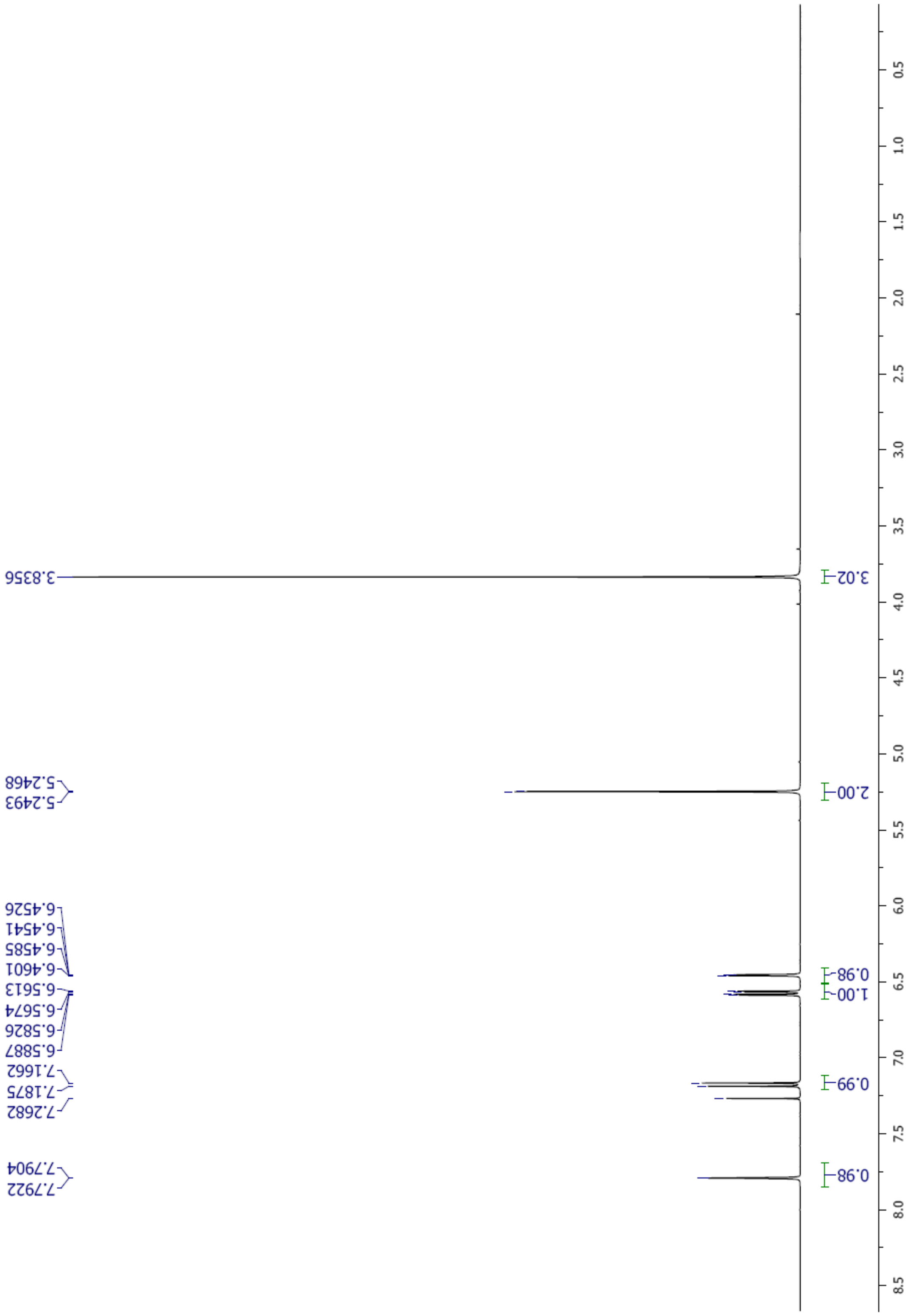
3.8356
5.2468
5.2493
6.4526
6.4541
6.4585
6.4601
6.5613
6.5674
6.5826
6.5887
7.1662
7.1875
7.2682
7.7904
7.7922
3.02
2.00
0.98
1.00
0.99
0.98
0.5
1.0
1.5
2.0
2.5
3.0
3.5
4.0
4.5
5.0
5.5
6.0
6.5
7.0
7.5
8.0
8.5

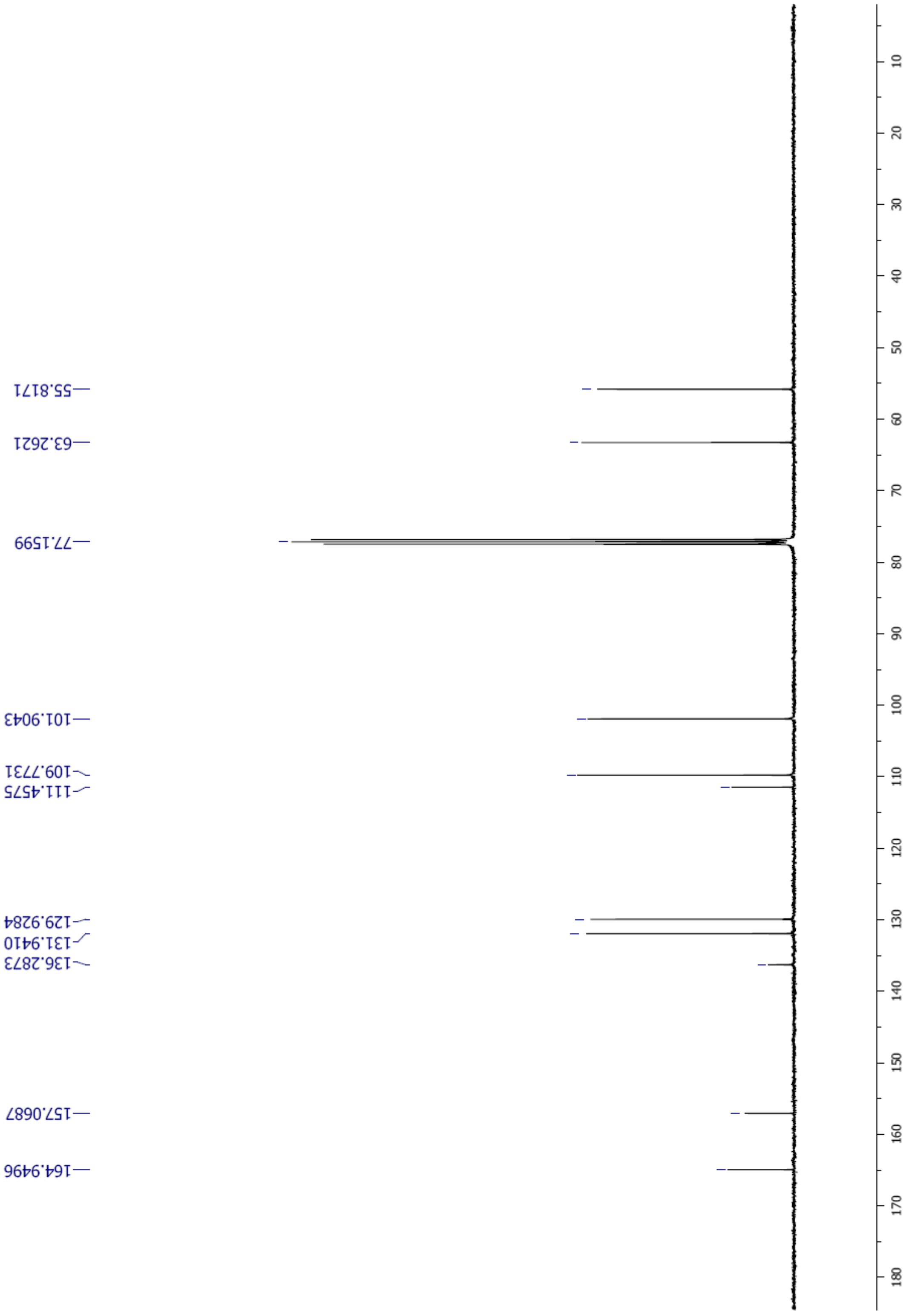
55.8171
63.2621
77.1599
101.9043
109.7731
111.4575
129.9284
131.9410
136.2873
157.0687
164.9496
10
20
30
40
50
60
70
80
90
100
110
120
130
140
150
160
170
180

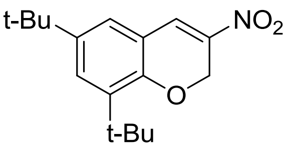
t-Bu
NO2
O
t-Bu

1e

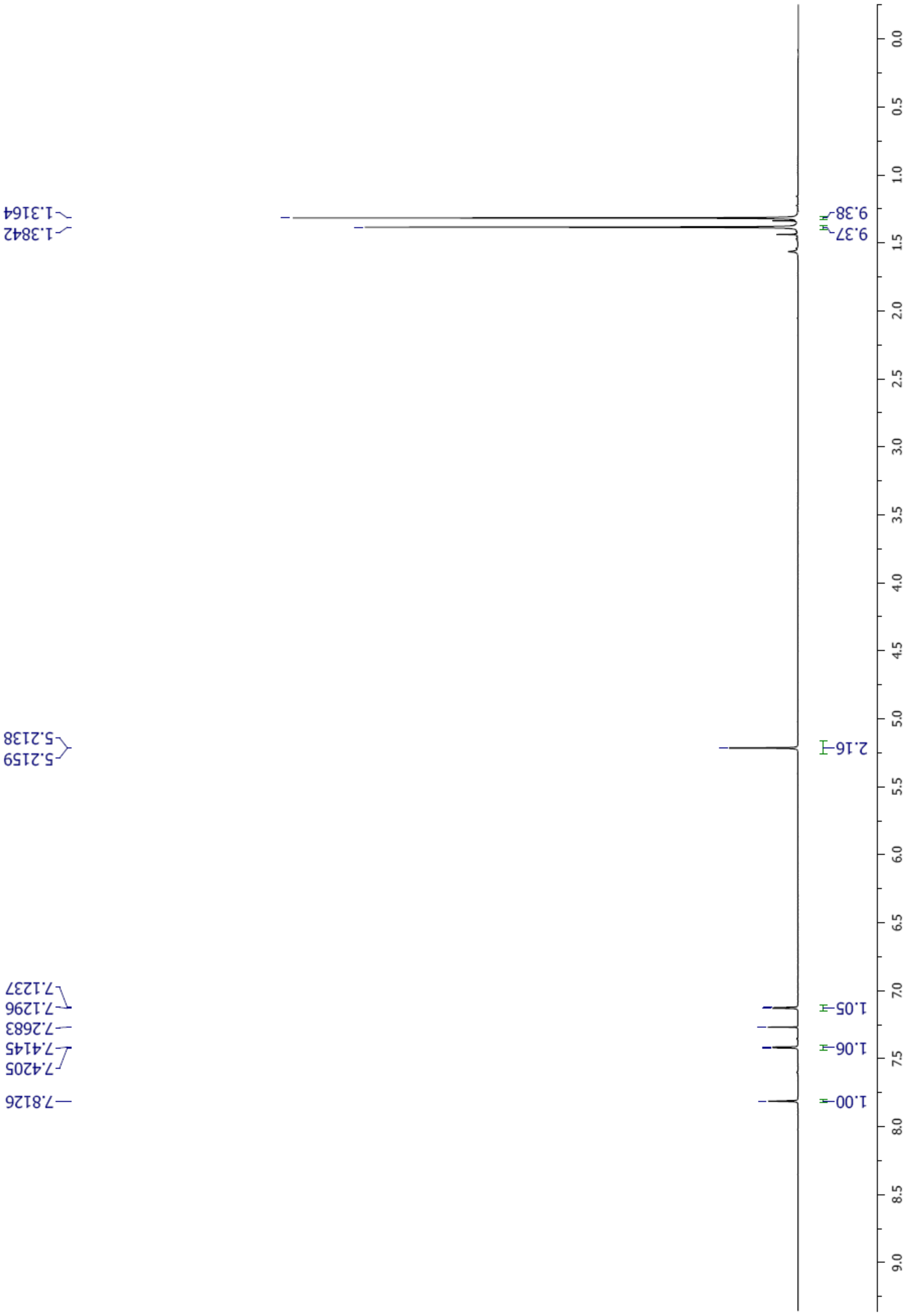
1.3164
1.3842
5.2138
5.2159
7.1237
7.1296
7.2683
7.4145
7.4205
7.8126
9.38
9.37
2.16
1.05
1.06
1.00
0.0
0.5
1.0
1.5
2.0
2.5
3.0
3.5
4.0
4.5
5.0
5.5
6.0
6.5
7.0
7.5
8.0
8.5
9.0

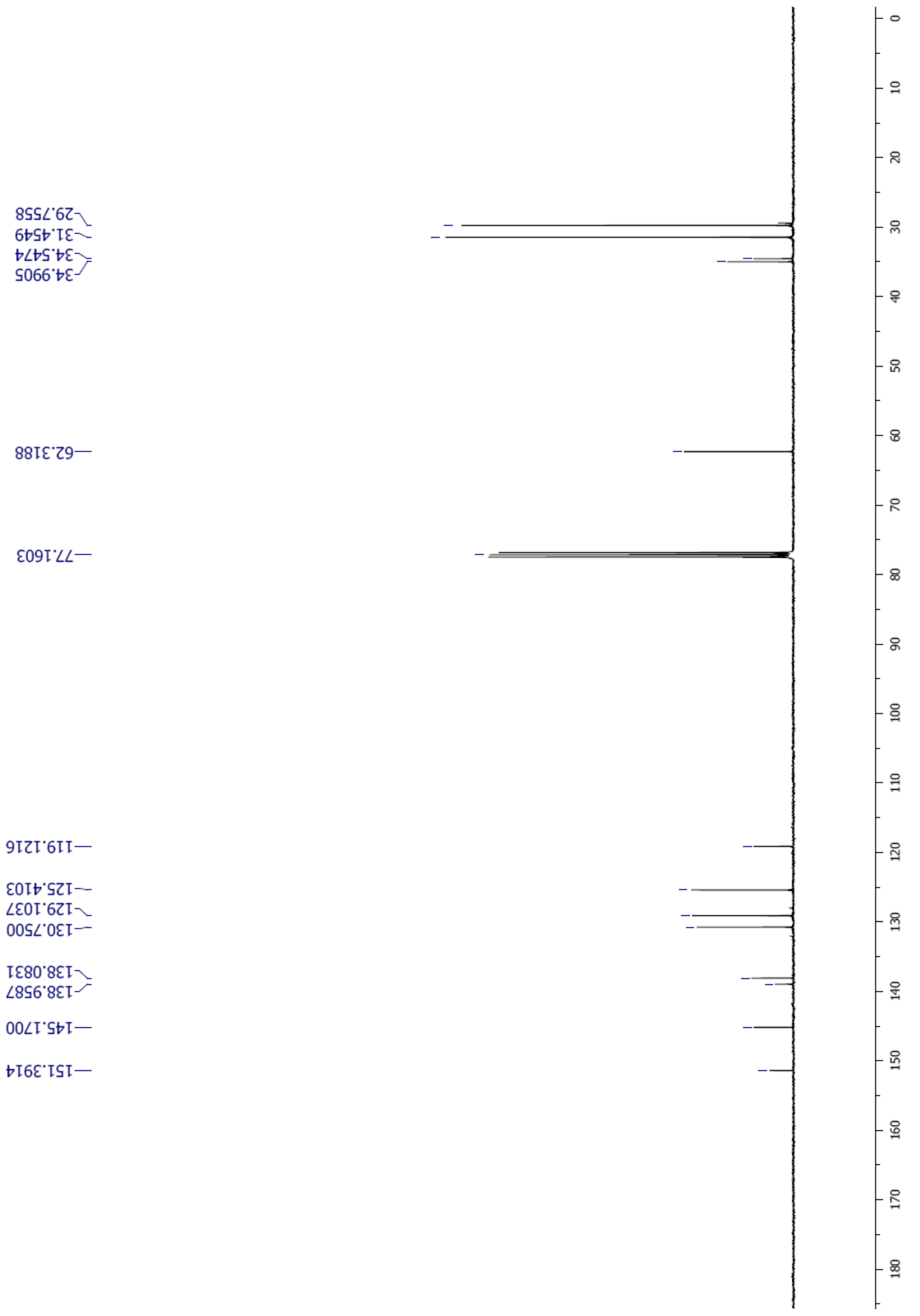
29.7558
31.4549
34.5474
34.9905
62.3188
77.1603
119.1216
125.4103
129.1037
130.7500
138.0831
138.9587
145.1700
151.3914

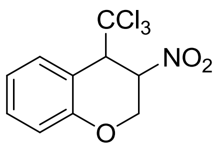
CCl3
NO2
O

2a

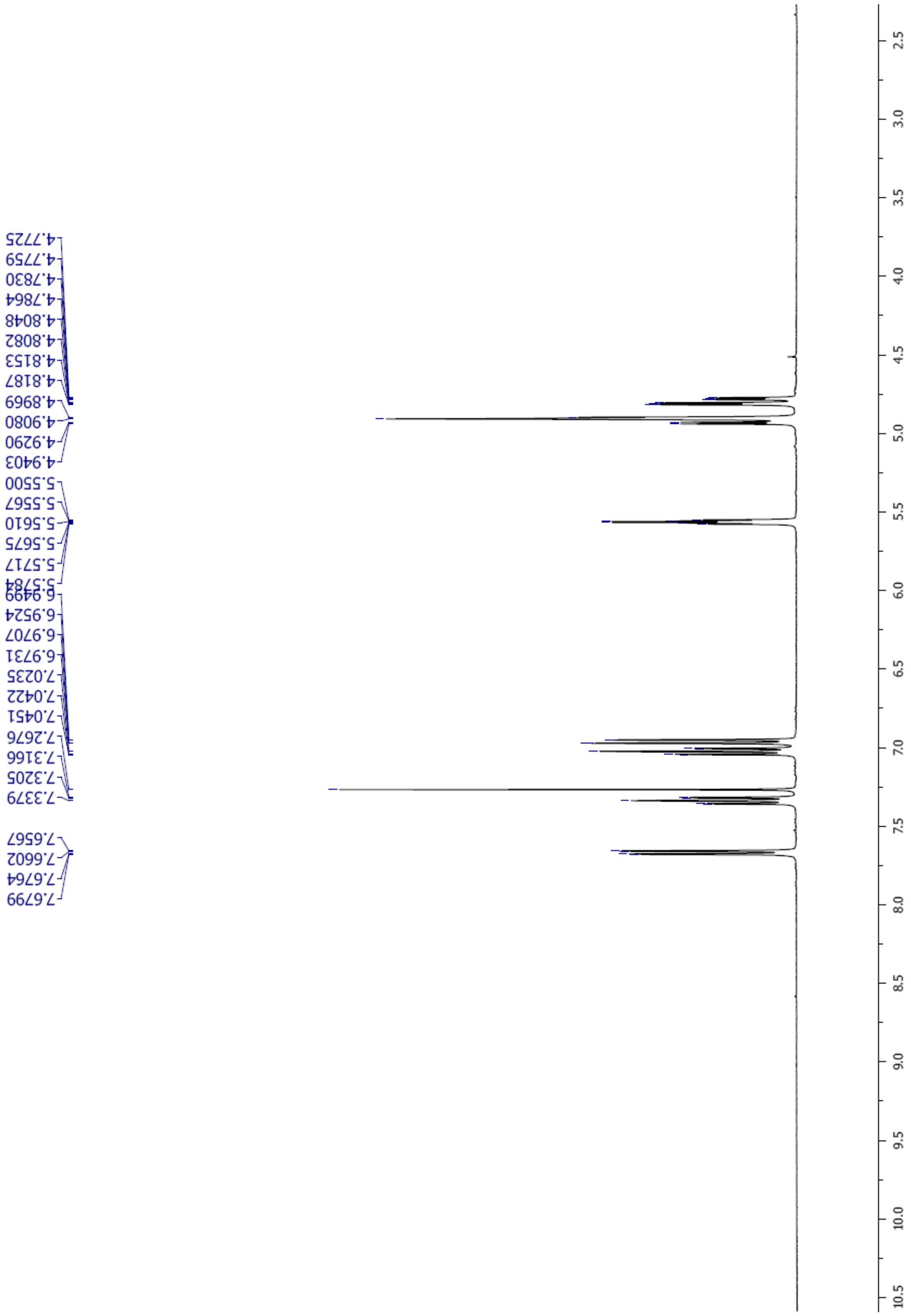
4.7725
4.7759
4.7830
4.7864
4.8048
4.8082
4.8153
4.8187
4.8969
4.9080
4.9290
4.9403
5.5500
5.5567
5.5610
5.5675
5.5717
5.5784
6.9499
6.9524
6.9707
6.9731
7.0235
7.0422
7.0451
7.2676
7.3166
7.3205
7.3379
7.6567
7.6602
7.6764
7.6799
2.5
3.0
3.5
4.0
4.5
5.0
5.5
6.0
6.5
7.0
7.5
8.0
8.5
9.0
9.5
10.0
10.5

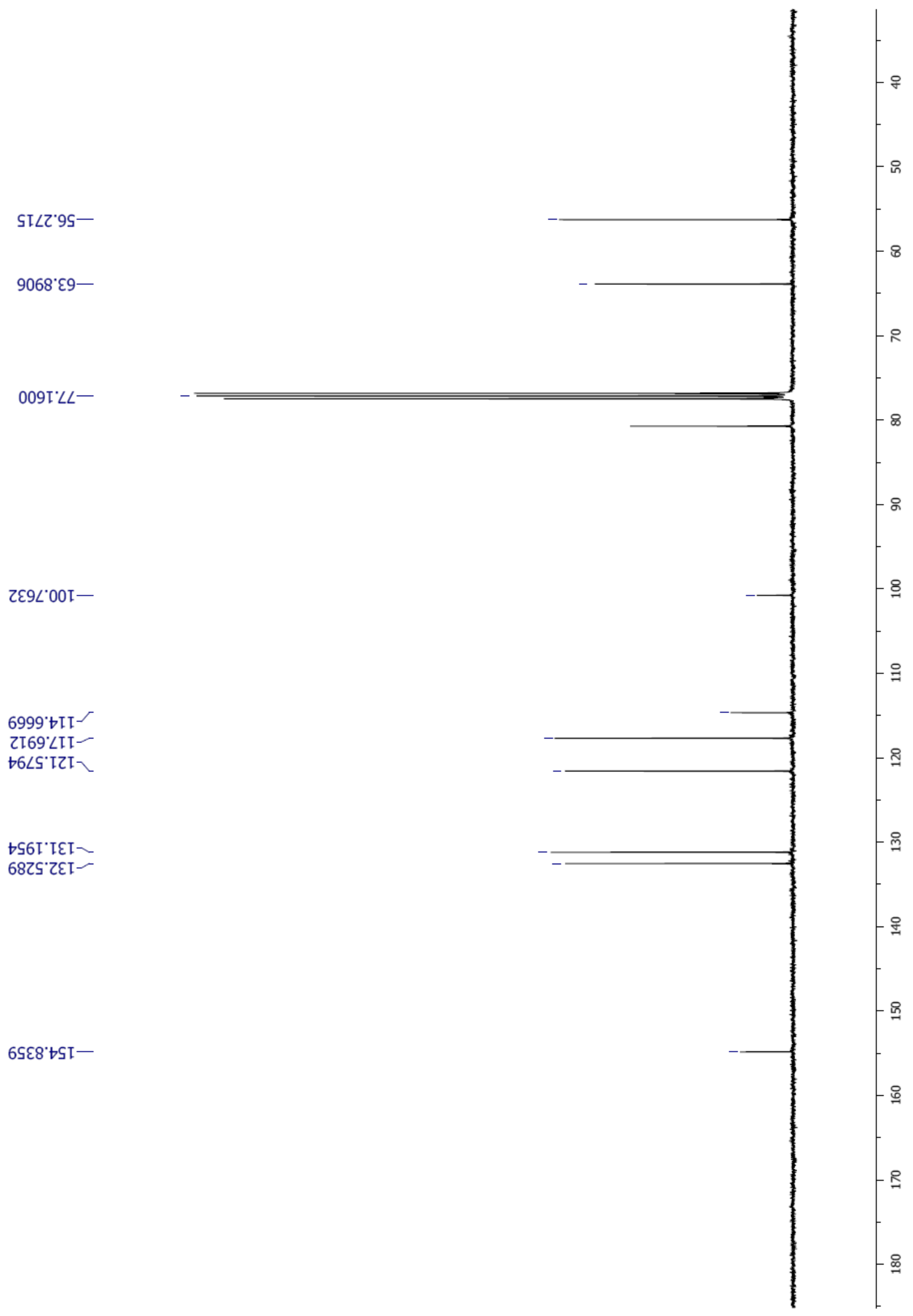
56.2715
63.8906
77.1600
100.7632
114.6669
117.6912
121.5794
131.1954
132.5289
154.8359
40
50
60
70
80
90
100
110
120
130
140
150
160
170
180

2a ^{1}H-^{13}C HSQC (400 MHz)

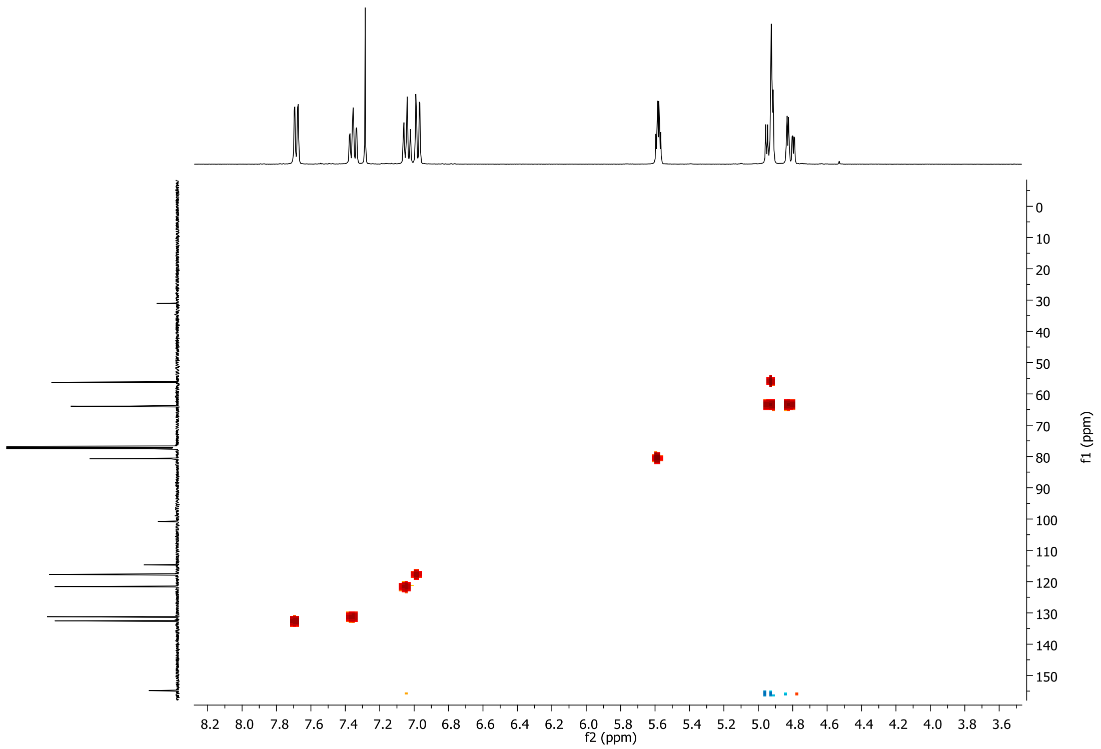

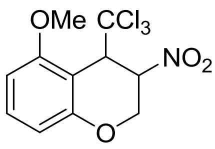

OMe
CCl3
NO2
O

2b

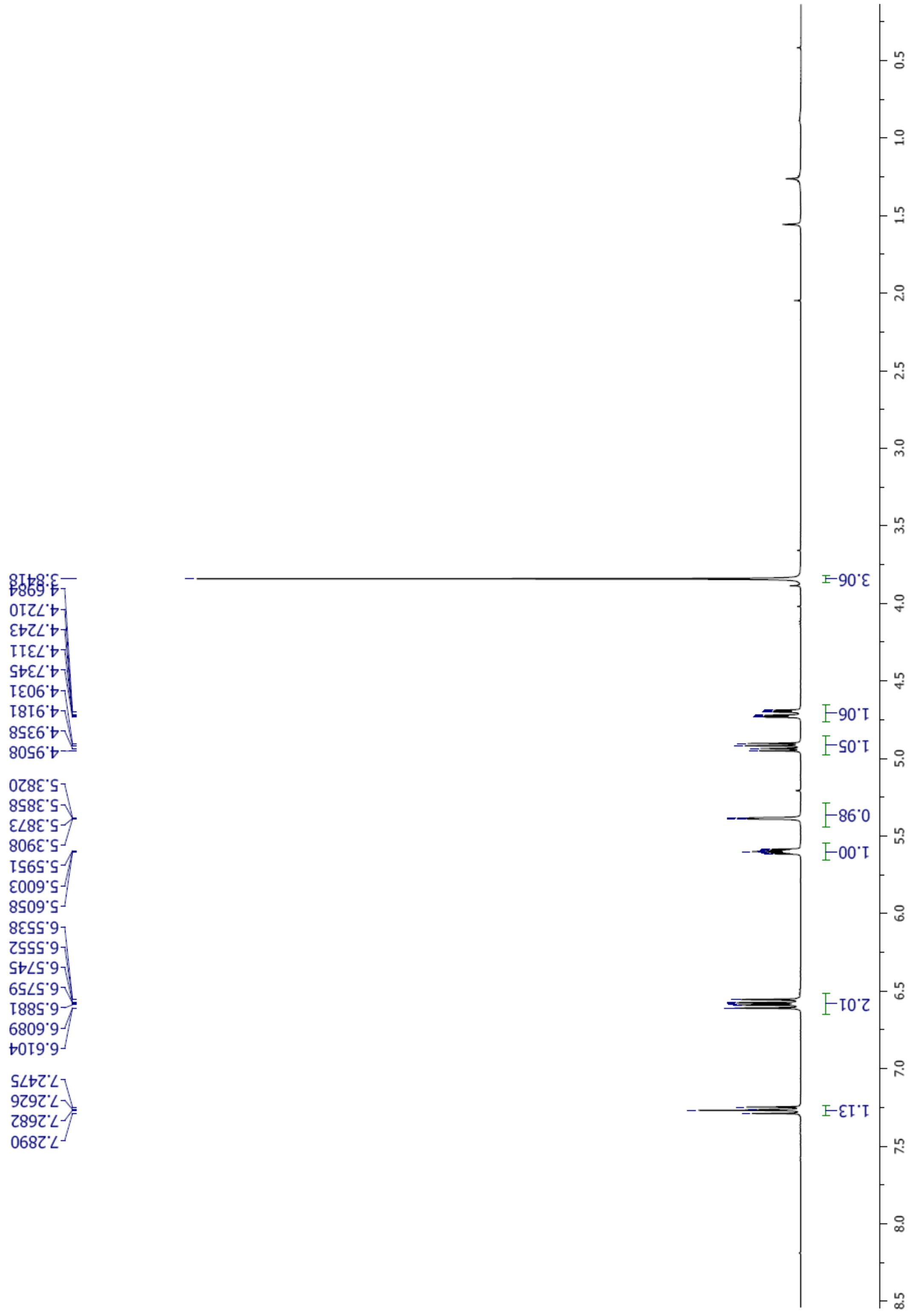

3.8418
4.6984
4.7210
4.7243
4.7311
4.7345
4.9031
4.9181
4.9358
4.9508
5.3820
5.3858
5.3873
5.3908
5.5951
5.6003
5.6058
6.5538
6.5552
6.5745
6.5759
6.5881
6.6089
6.6104
7.2475
7.2626
7.2682
7.2890
3.06
1.06
1.05
0.98
1.00
2.01
1.13
0.5
1.0
1.5
2.0
2.5
3.0
3.5
4.0
4.5
5.0
5.5
6.0
6.5
7.0
7.5
8.0
8.5

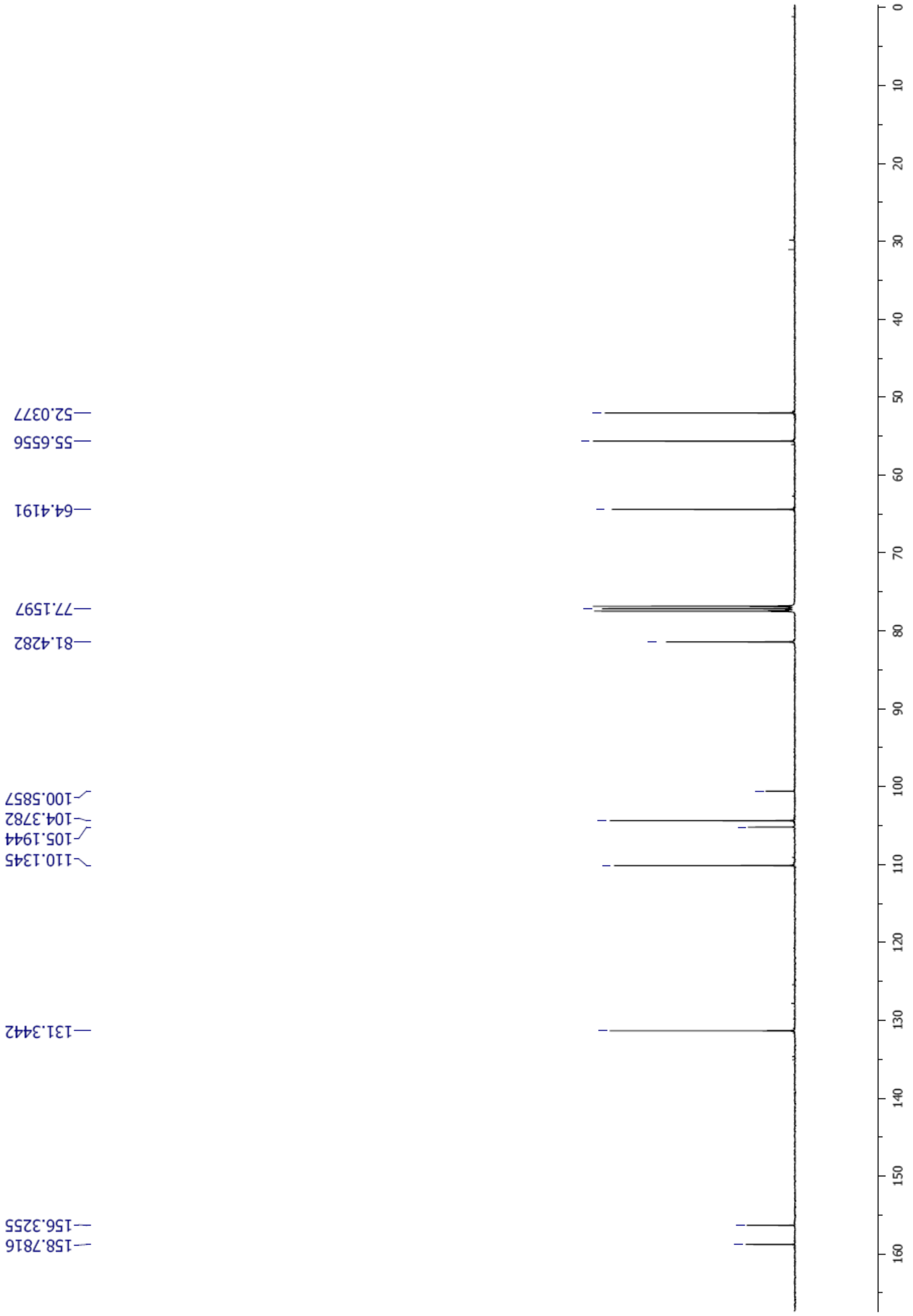

52.0377
55.6556
64.4191
77.1597
81.4282
100.5857
104.3782
105.1944
110.1345
131.3442
156.3255
158.7816
0
10
20
30
40
50
60
70
80
90
100
110
120
130
140
150
160

2b ^{1}H-^{1}H COSY (400 MHz)

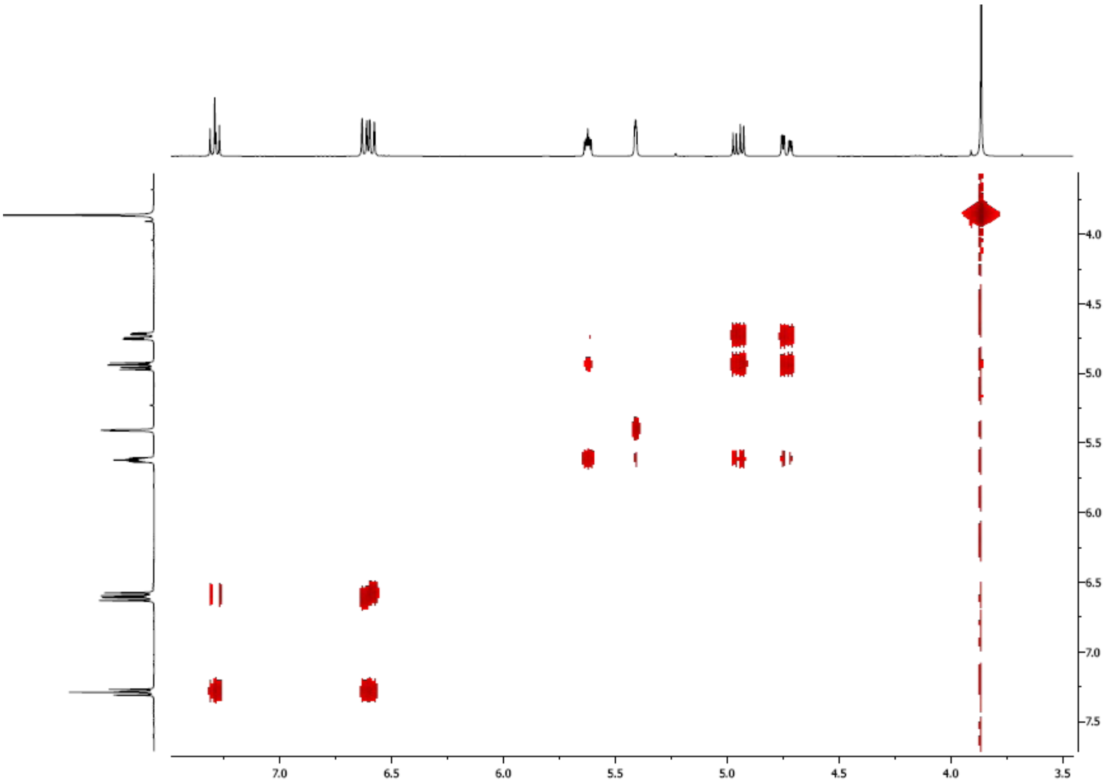

2b DEPT 135C (100 MHz)

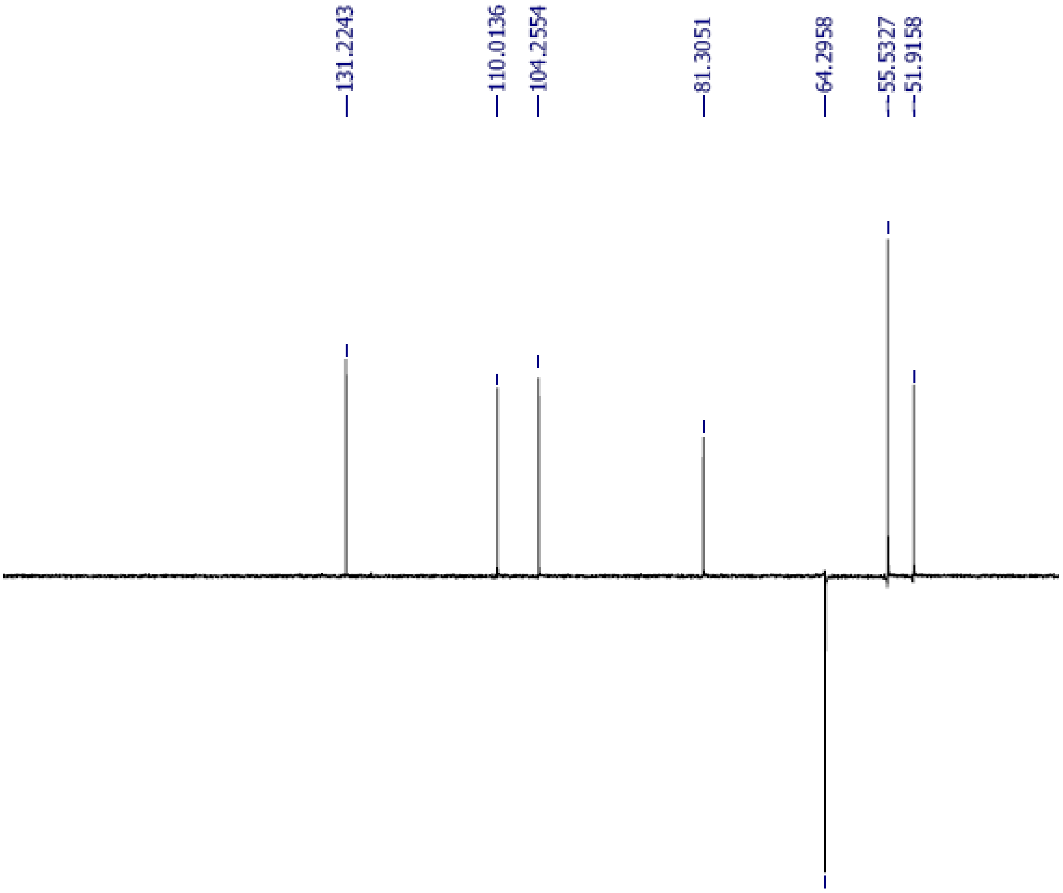

2b ^{1}H-^{13}C HSQC (400 MHz)

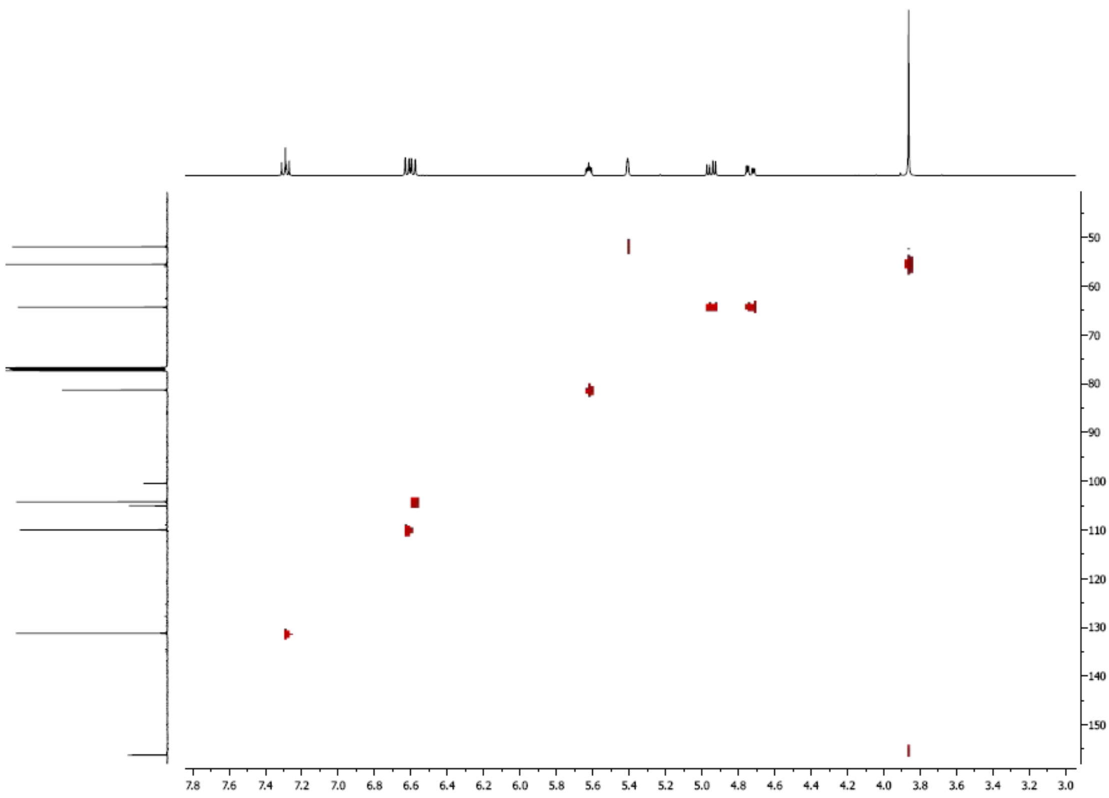

2b ^{1}H-^{13}C HMBC (400 MHz)

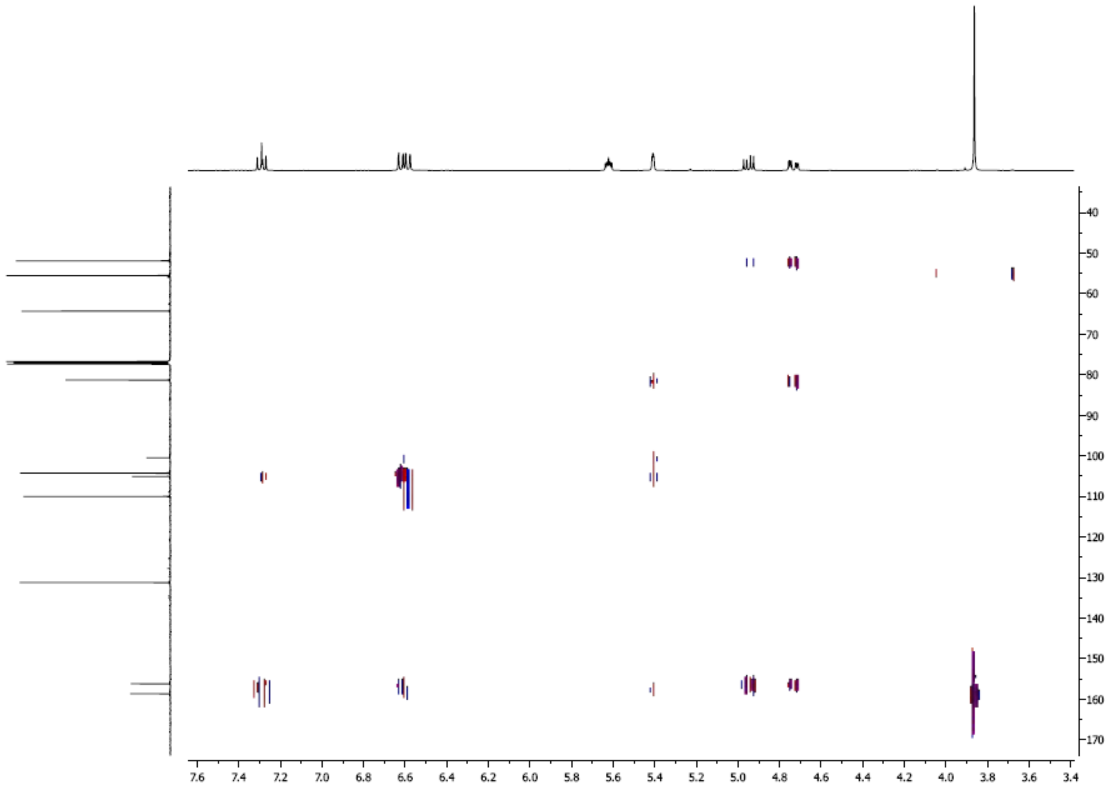

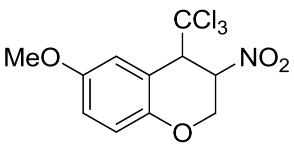
CCl3
MeO
NO2
O

2c

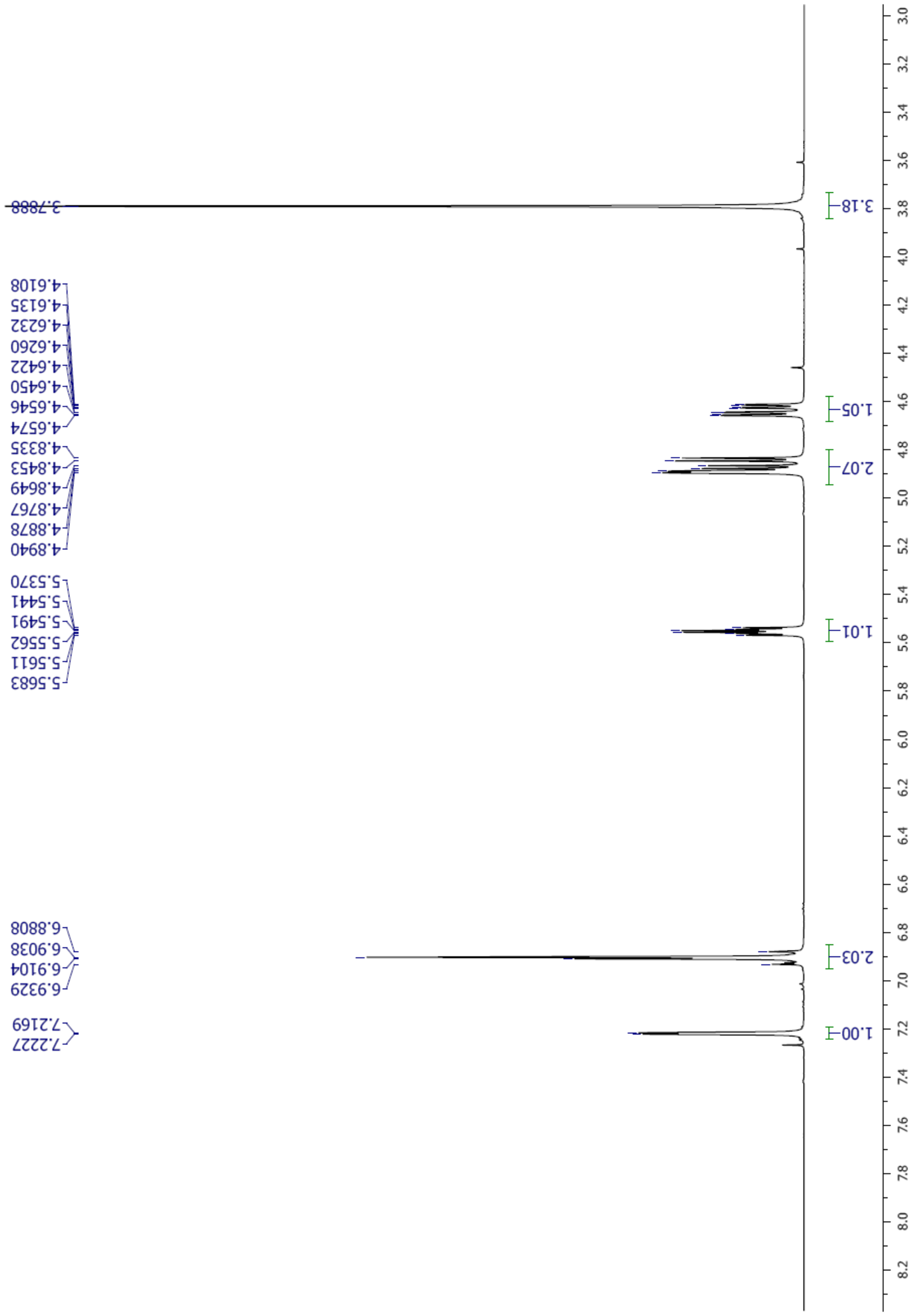
3.7888
4.6108
4.6135
4.6232
4.6260
4.6422
4.6450
4.6546
4.6574
4.8335
4.8453
4.8649
4.8767
4.8878
4.8940
5.5370
5.5441
5.5491
5.5562
5.5611
5.5683
6.8808
6.9038
6.9104
6.9329
7.2169
7.2227
3.18
1.05
2.07
1.01
2.03
1.00
3.0
3.2
3.4
3.6
3.8
4.0
4.2
4.4
4.6
4.8
5.0
5.2
5.4
5.6
5.8
6.0
6.2
6.4
6.6
6.8
7.0
7.2
7.4
7.6
7.8
8.0
8.2

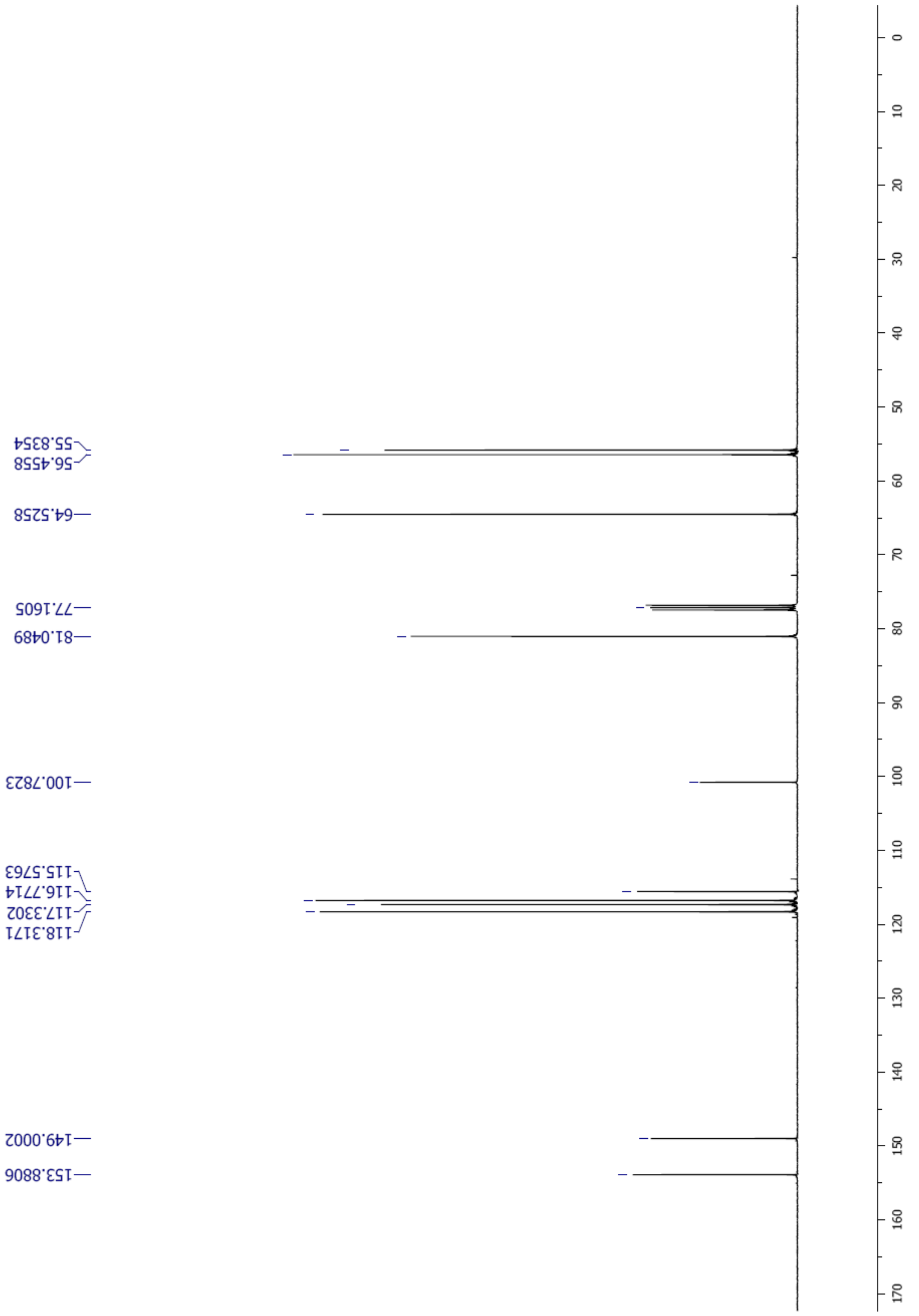
55.8354
56.4558
64.5258
77.1605
81.0489
100.7823
115.5763
116.7714
117.3302
118.3171
149.0002
153.8806
0
10
20
30
40
50
60
70
80
90
100
110
120
130
140
150
160
170

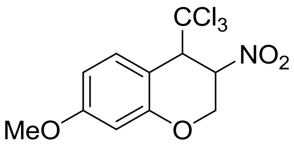
CCl3
NO2
MeO
O

2d

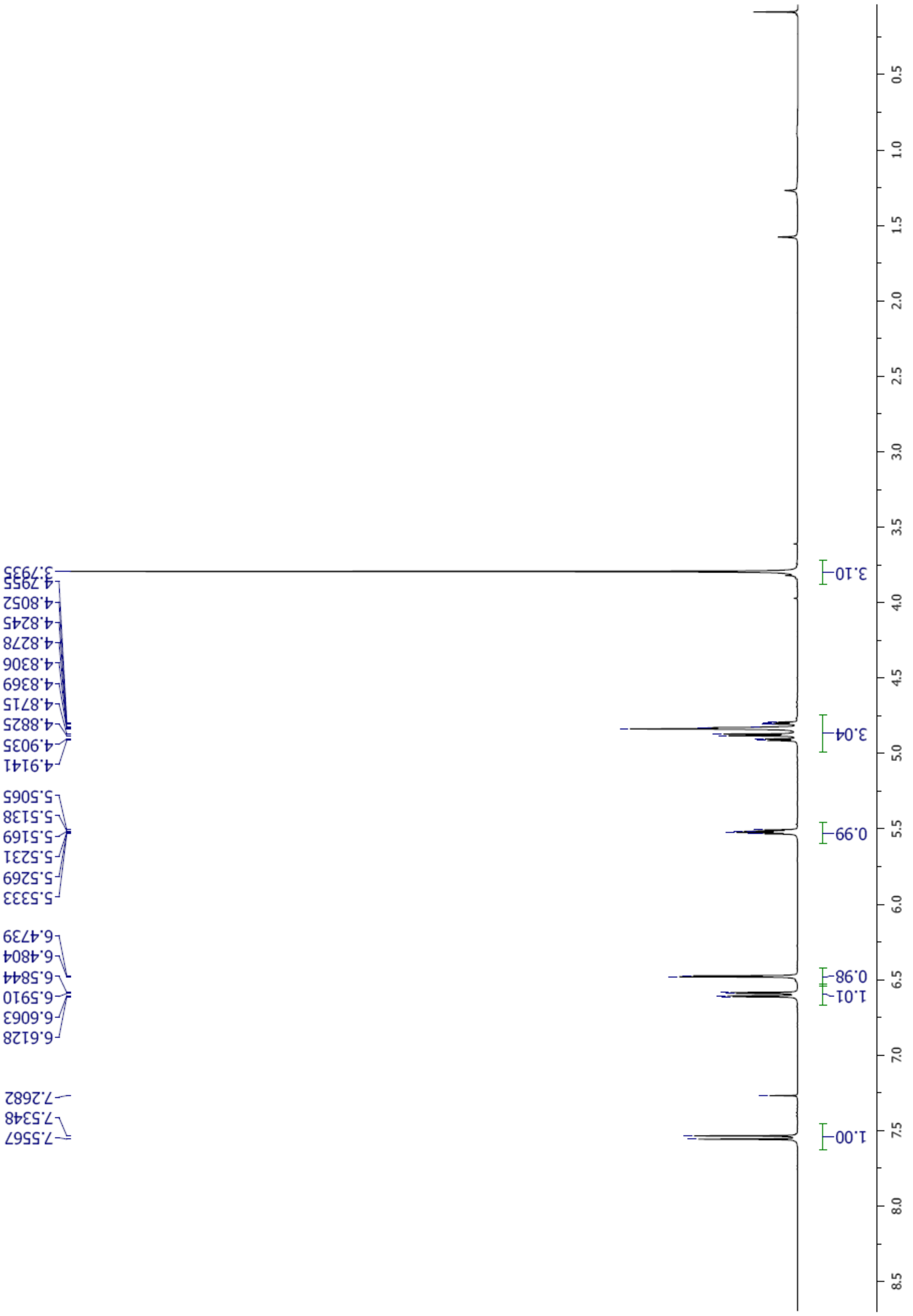
3.7935
4.7955
4.8052
4.8245
4.8278
4.8306
4.8369
4.8715
4.8825
4.9035
4.9141
5.5065
5.5138
5.5169
5.5231
5.5269
5.5333
6.4739
6.4804
6.5844
6.5910
6.6063
6.6128
7.2682
7.5348
7.5567
3.10
3.04
0.99
0.98
1.01
1.00
0.5
1.0
1.5
2.0
2.5
3.0
3.5
4.0
4.5
5.0
5.5
6.0
6.5
7.0
7.5
8.0
8.5

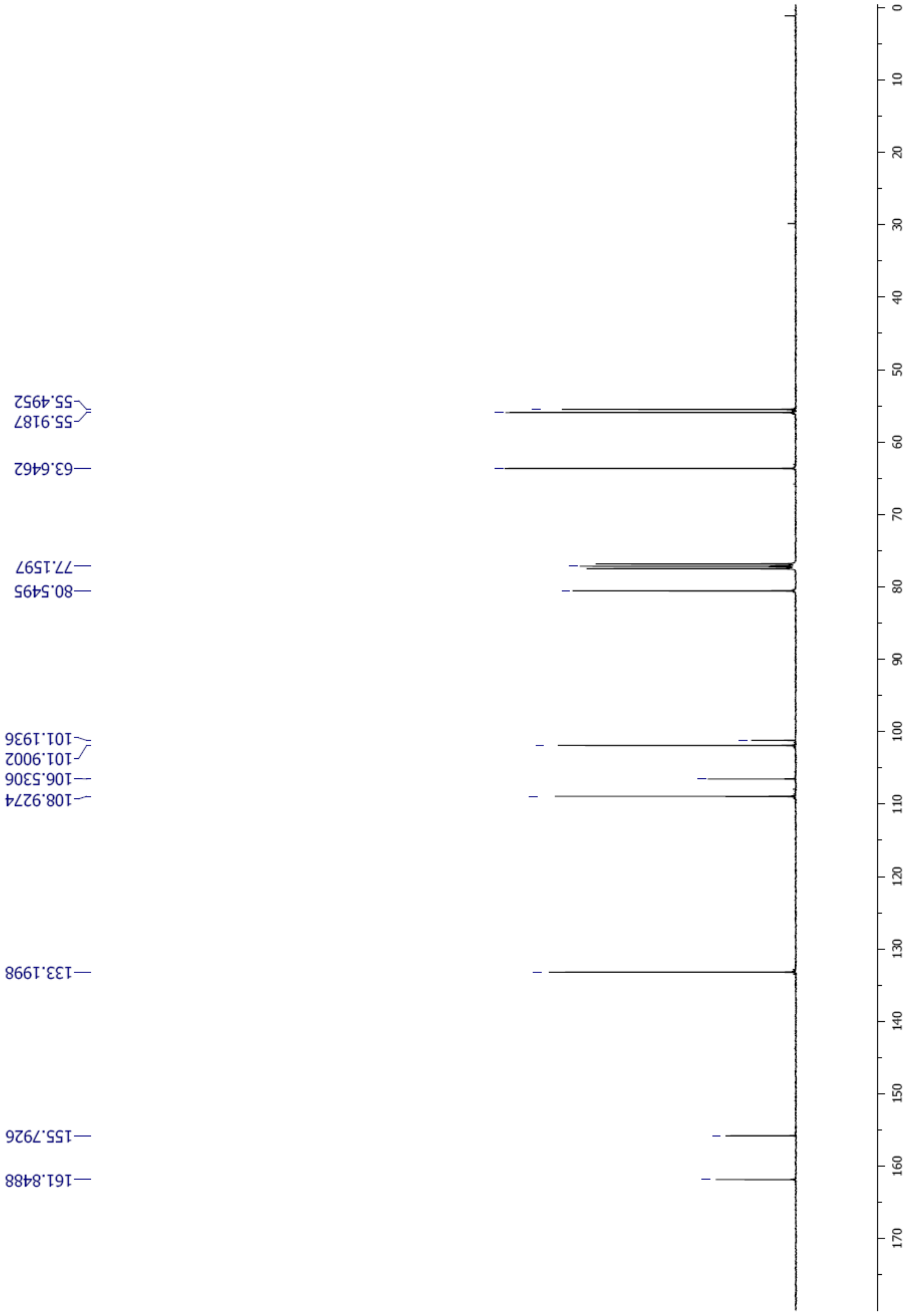
55.4952
55.9187
63.6462
77.1597
80.5495
101.1936
101.9002
106.5306
108.9274
133.1998
155.7926
161.8488

^{1}H-^{1}H COSY (400 MHz)

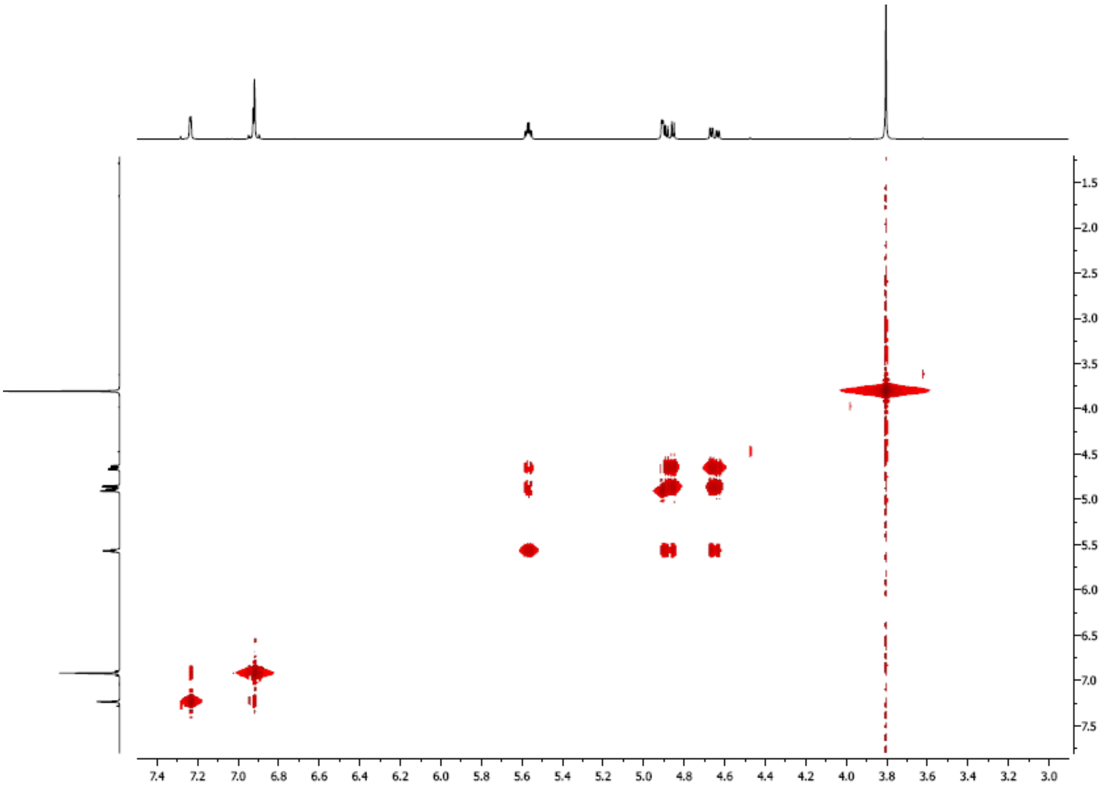

^{13}C DEPT 135 (100 MHz)

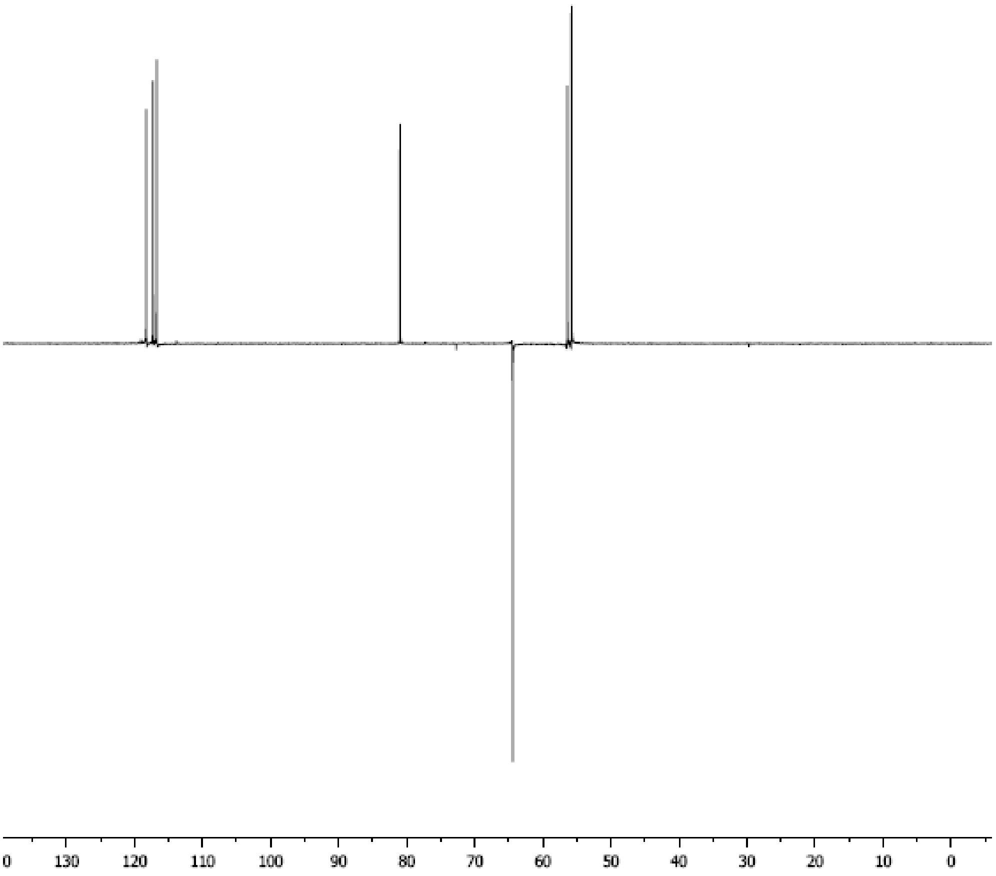

^{1}H-^{13}C HSQC (400 MHz)

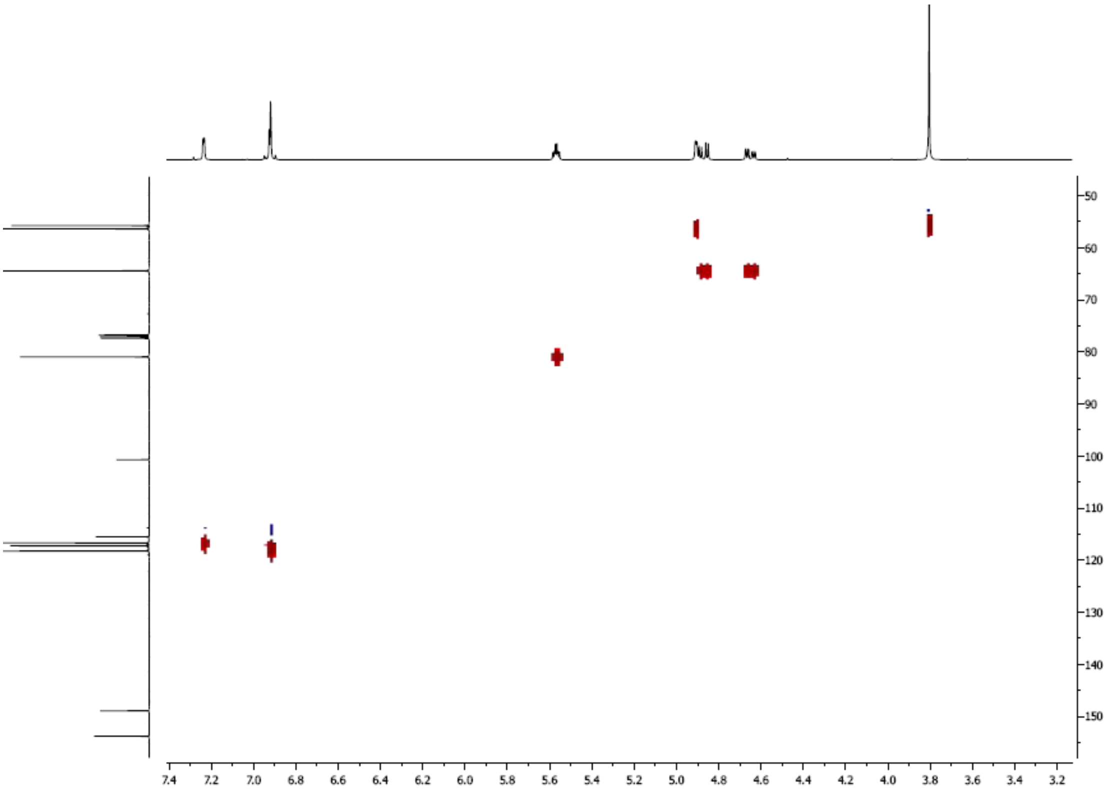

^{1}H-^{13}C HMBC (400 MHz)

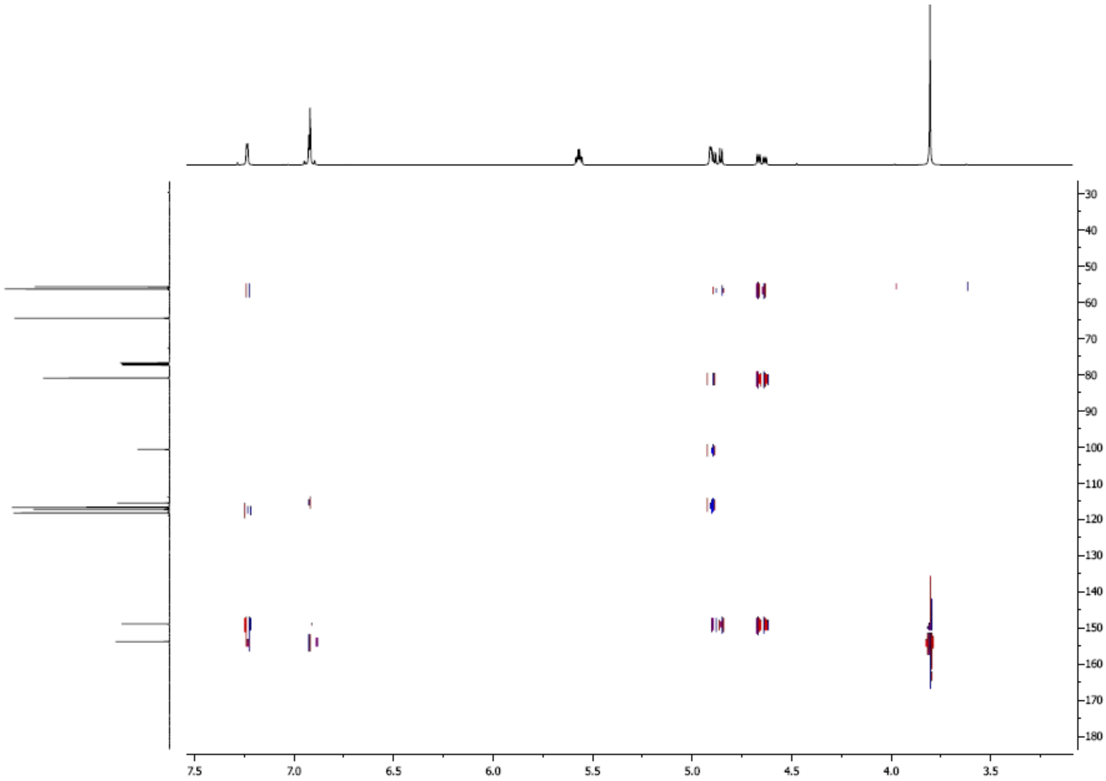

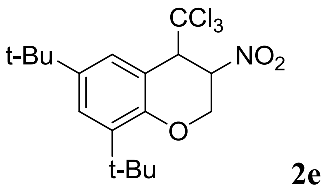
CCl3
t-Bu
NO2
O
t-Bu

2e

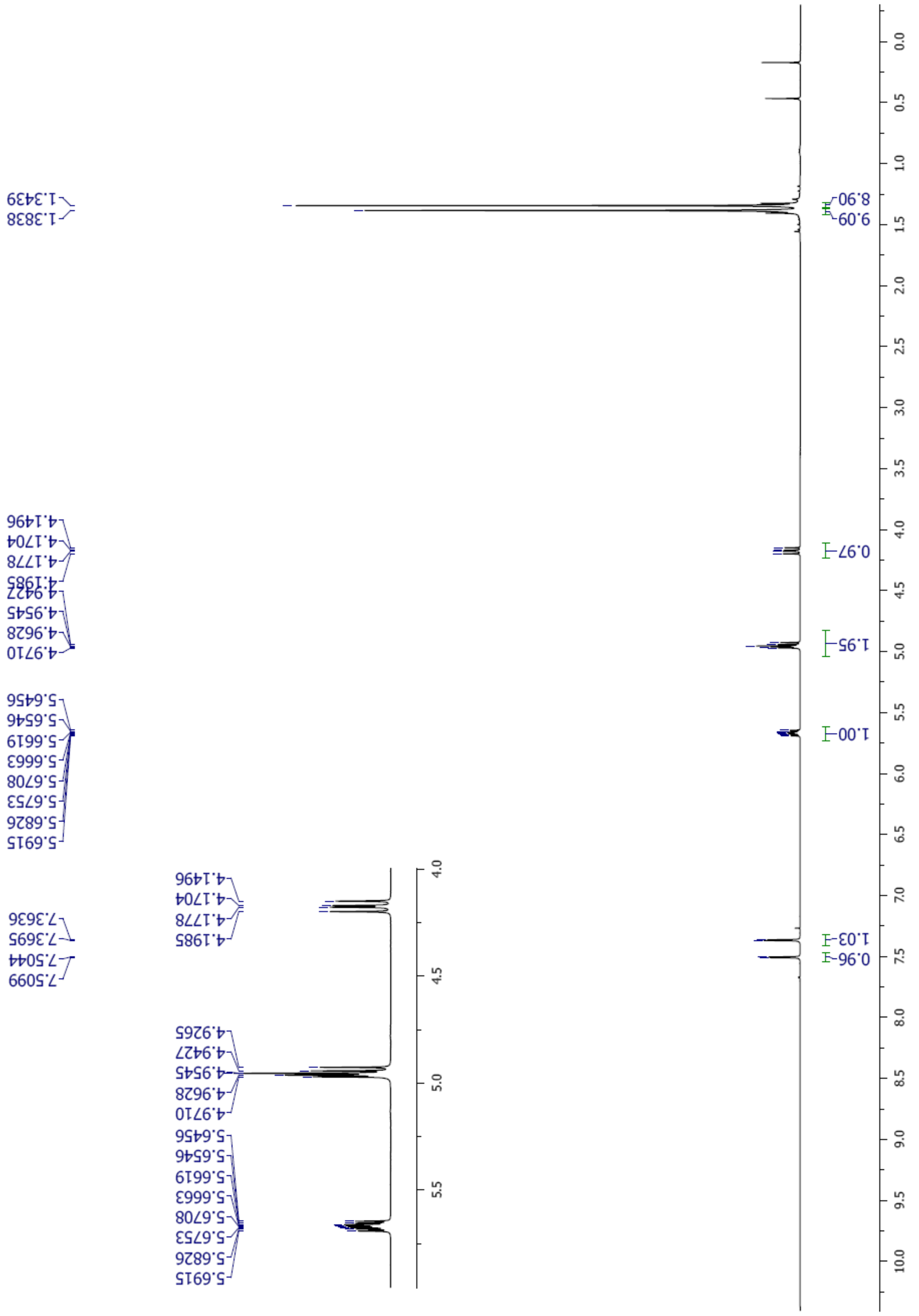
1.3439
1.3838
4.1496
4.1704
4.1778
4.1985
4.9427
4.9545
4.9628
4.9710
5.6456
5.6546
5.6619
5.6663
5.6708
5.6753
5.6826
5.6915
7.3636
7.3695
7.5044
7.5099
8.90
9.09
0.97
1.95
1.00
1.03
0.96
4.9265

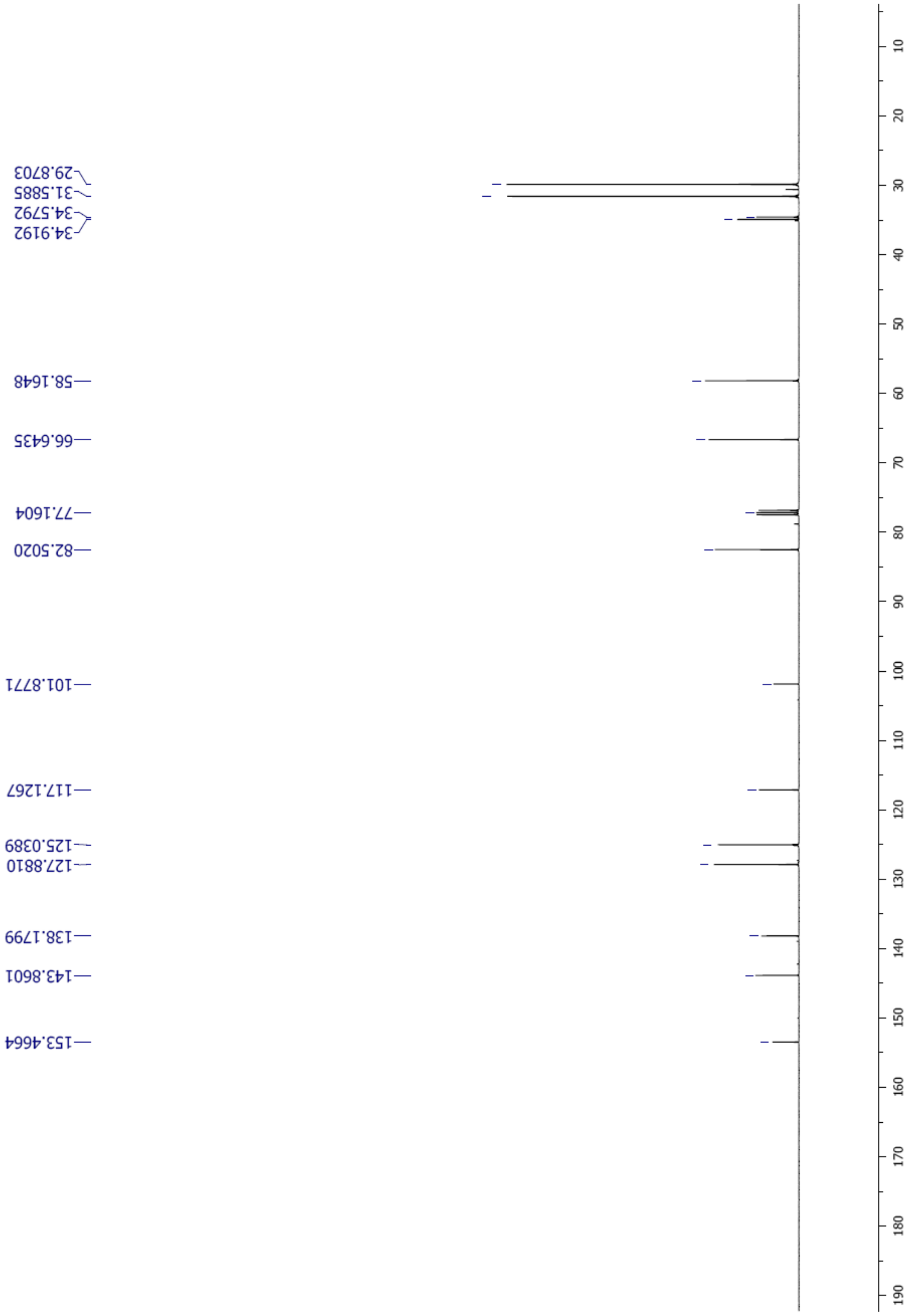
29.8703
31.5885
34.5792
34.9192
58.1648
66.6435
77.1604
82.5020
101.8771
117.1267
125.0389
127.8810
138.1799
143.8601
153.4664
10
20
30
40
50
60
70
80
90
100
110
120
130
140
150
160
170
180
190

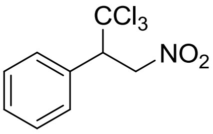
CCl3
NO2

2f

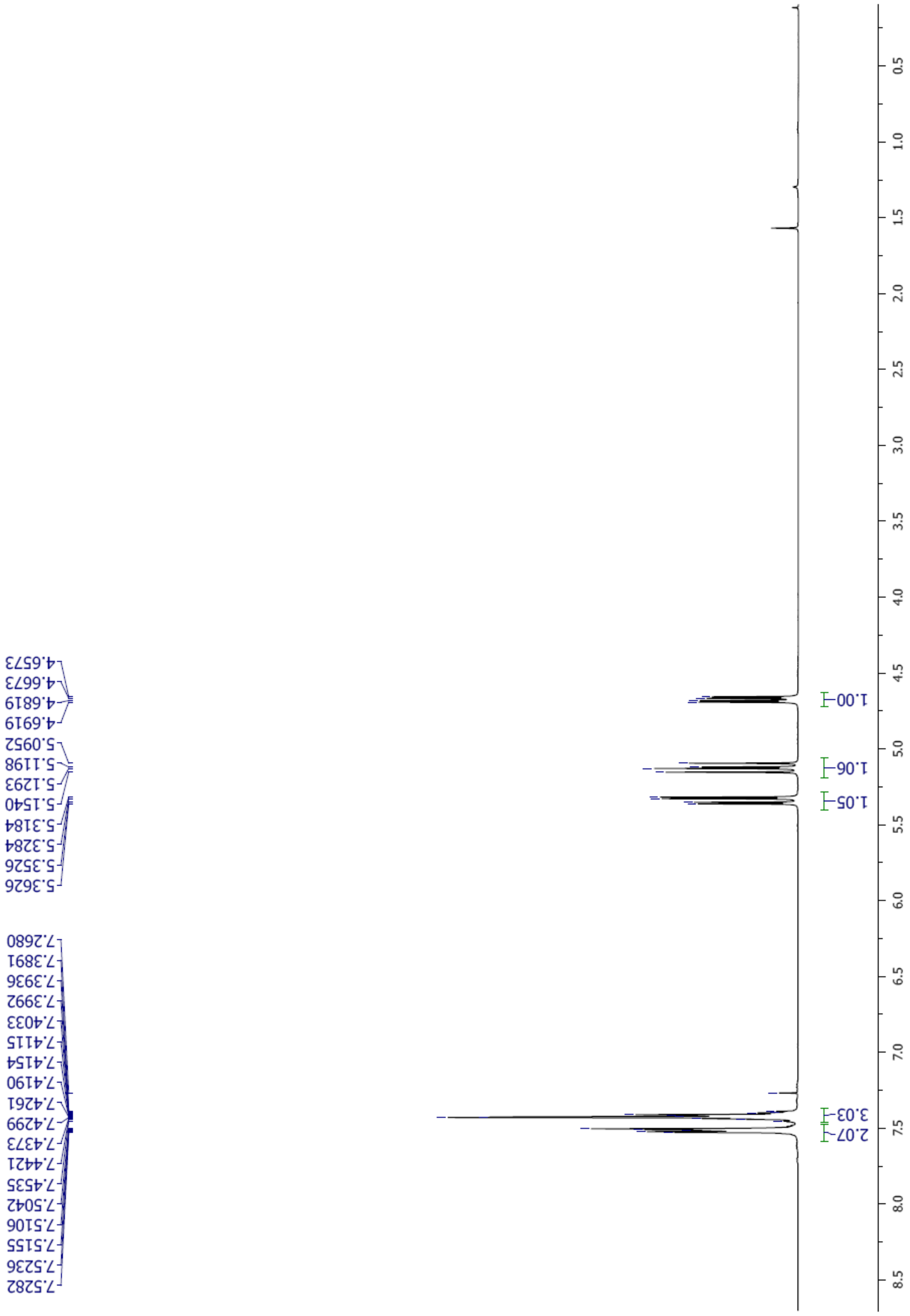
4.6573
4.6673
4.6819
4.6919
5.0952
5.1198
5.1293
5.1540
5.3184
5.3284
5.3526
5.3626
7.2680
7.3891
7.3936
7.3992
7.4033
7.4115
7.4154
7.4190
7.4261
7.4299
7.4373
7.4421
7.4535
7.5042
7.5106
7.5155
7.5236
7.5282
1.00
1.06
1.05
3.03
2.07
0.5
1.0
1.5
2.0
2.5
3.0
3.5
4.0
4.5
5.0
5.5
6.0
6.5
7.0
7.5
8.0
8.5

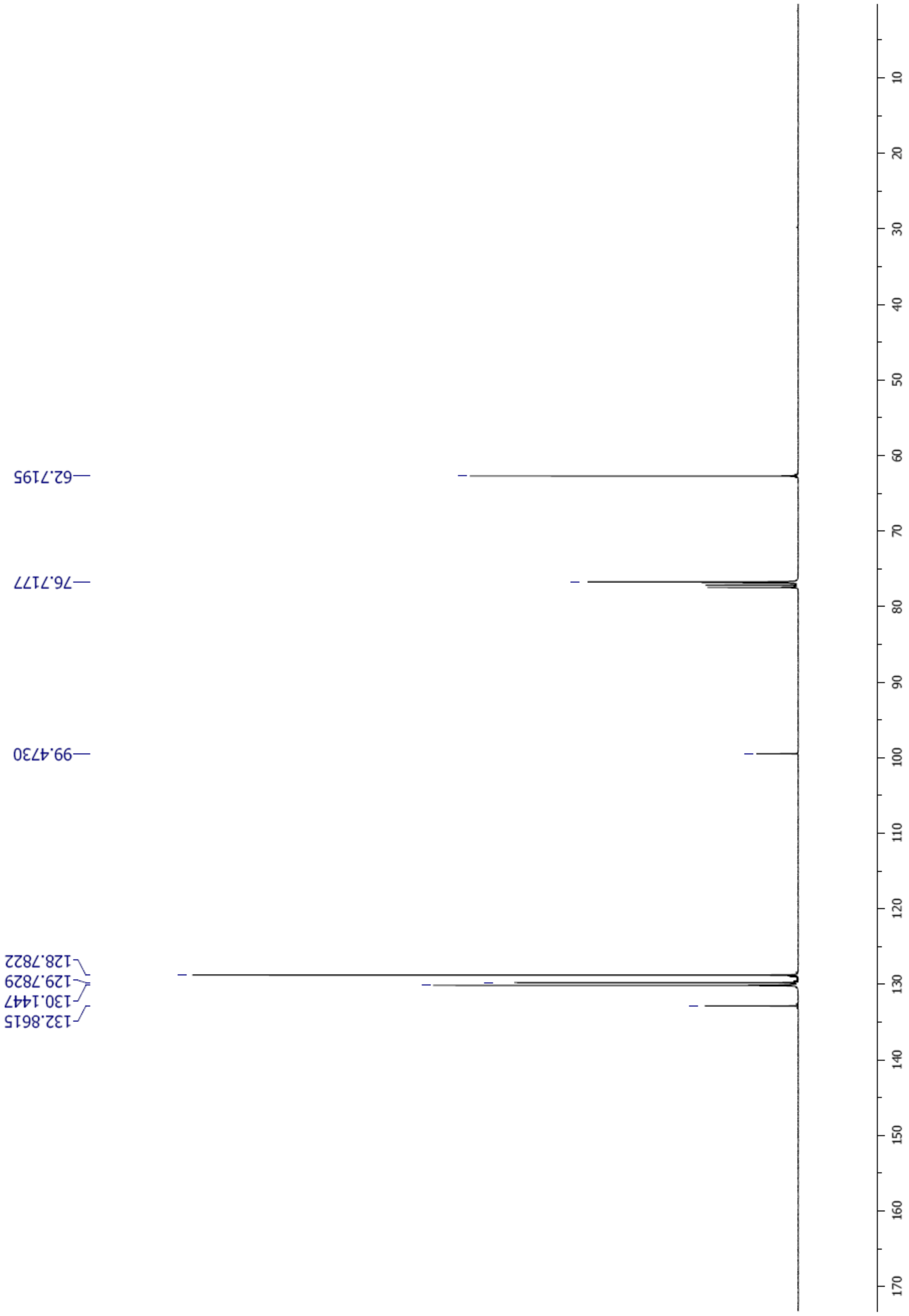
62.7195
76.7177
99.4730
128.7822
129.7829
130.1447
132.8615
10
20
30
40
50
60
70
80
90
100
110
120
130
140
150
160
170

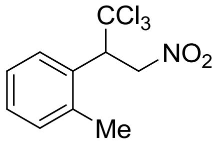
CCl3
NO2
Me

2g

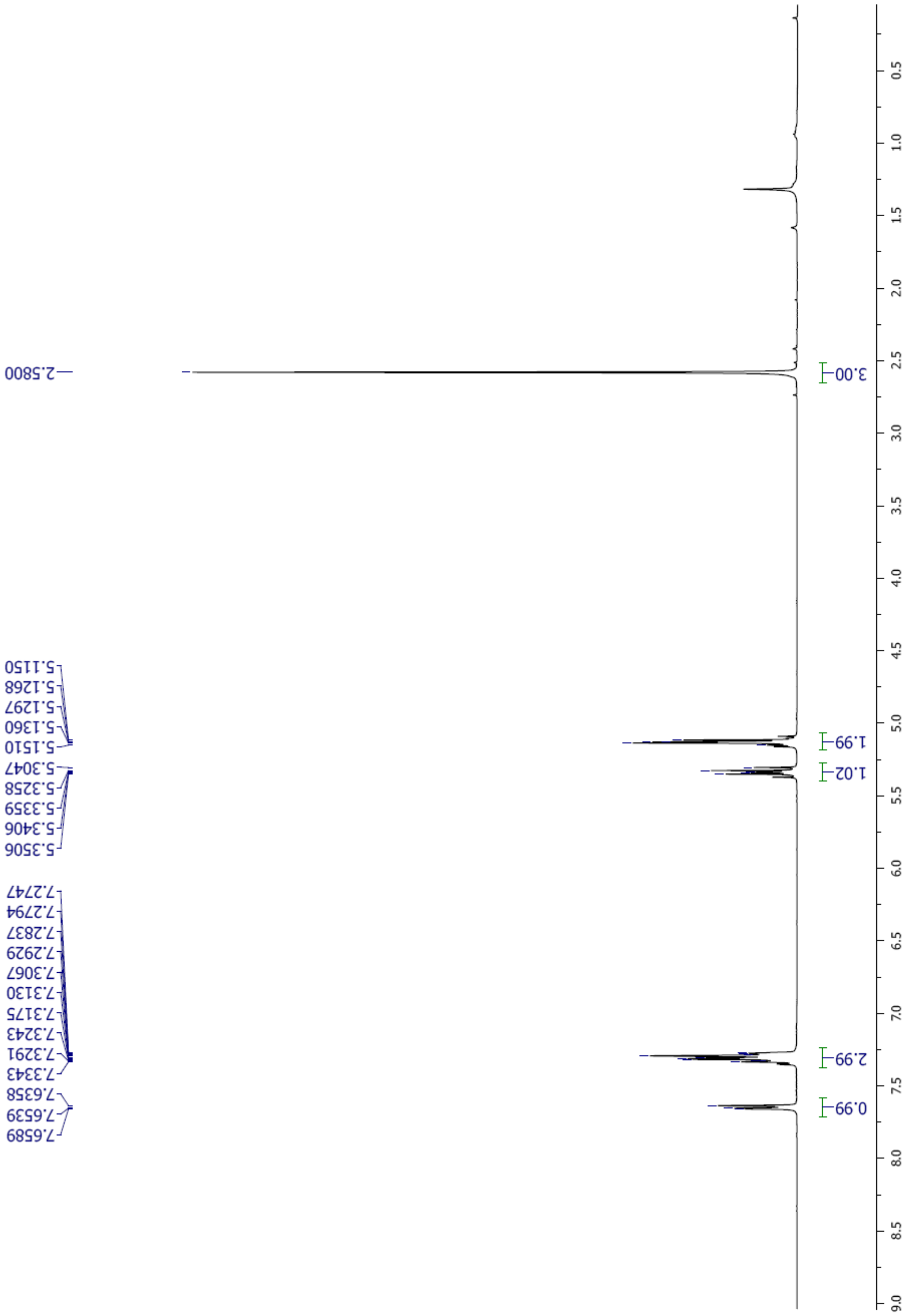
2.5800
5.1150
5.1268
5.1297
5.1360
5.1510
5.3047
5.3258
5.3359
5.3406
5.3506
7.2747
7.2794
7.2837
7.2929
7.3067
7.3130
7.3175
7.3243
7.3291
7.3343
7.6358
7.6539
7.6589
3.00
1.99
1.02
2.99
0.99
0.5
1.0
1.5
2.0
2.5
3.0
3.5
4.0
4.5
5.0
5.5
6.0
6.5
7.0
7.5
8.0
8.5
9.0

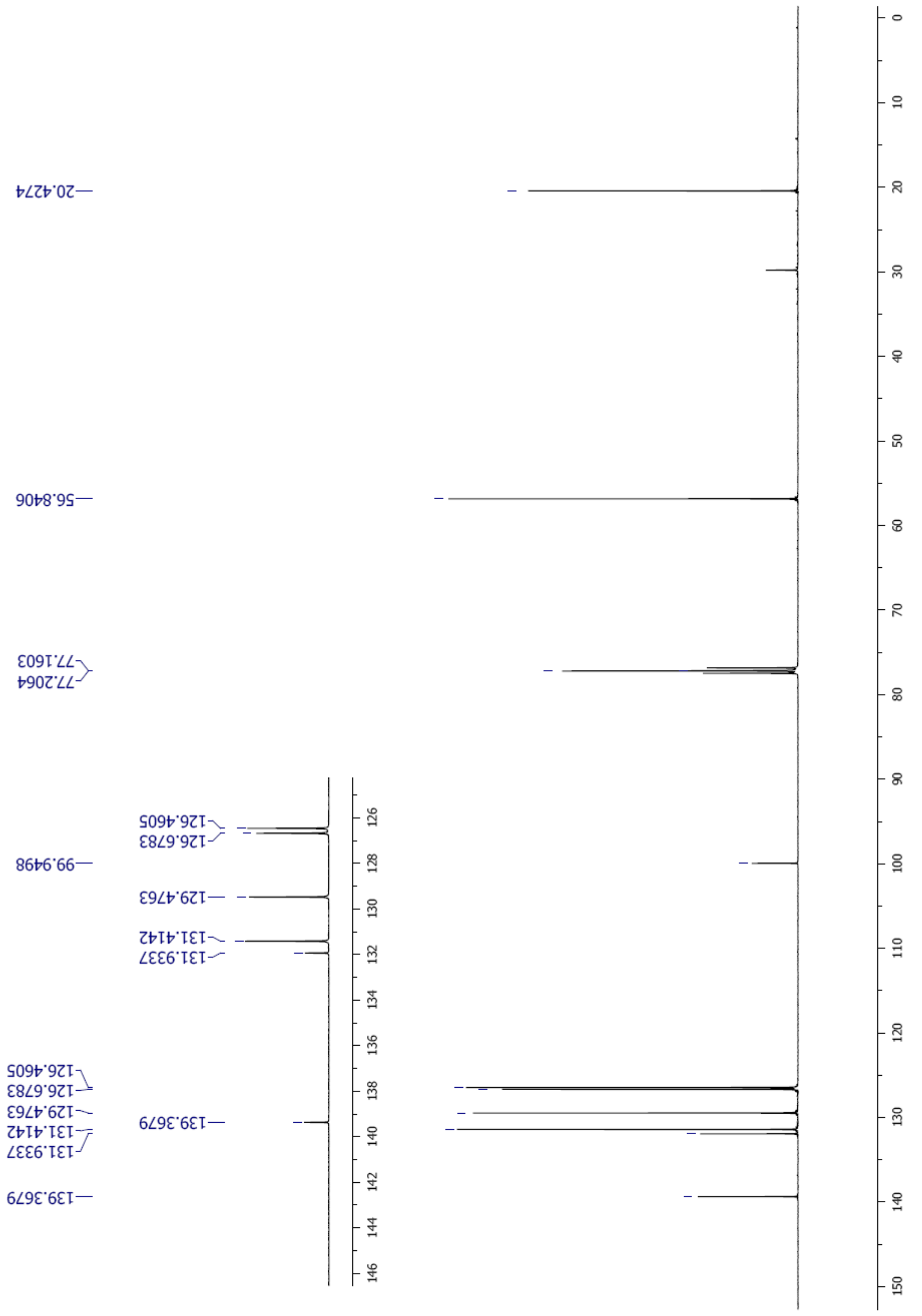
20.4274
56.8406
77.1603
77.2064
99.9498
126.4605
126.6783
129.4763
131.4142
131.9337
139.3679

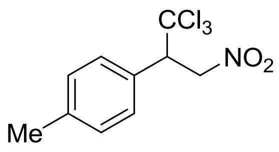
CCl3
NO2
Me

2h

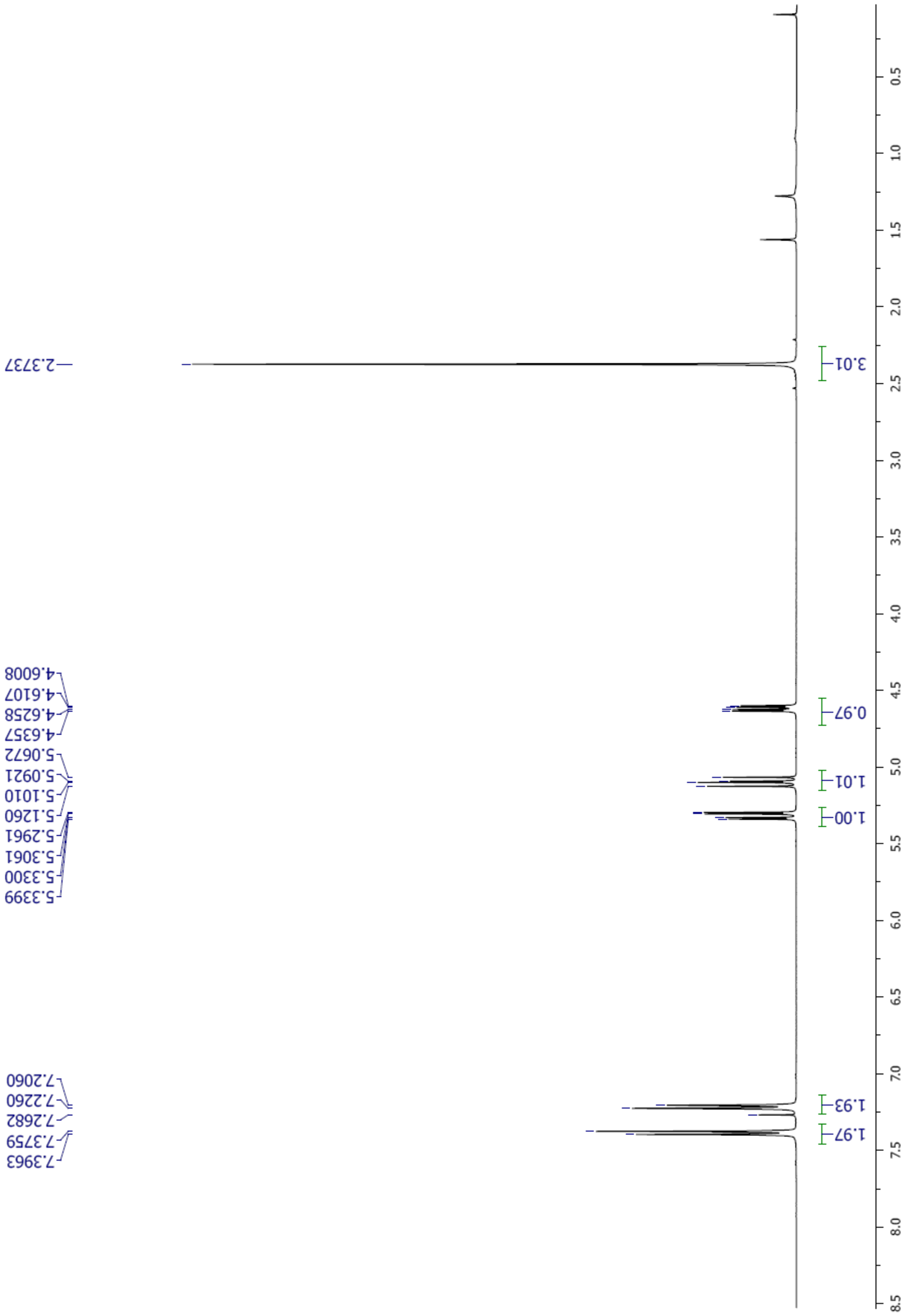
2.3737
4.6008
4.6107
4.6258
4.6357
5.0672
5.0921
5.1010
5.1260
5.2961
5.3061
5.3300
5.3399
7.2060
7.2260
7.2682
7.3759
7.3963
3.01
0.97
1.01
1.00
1.93
1.97

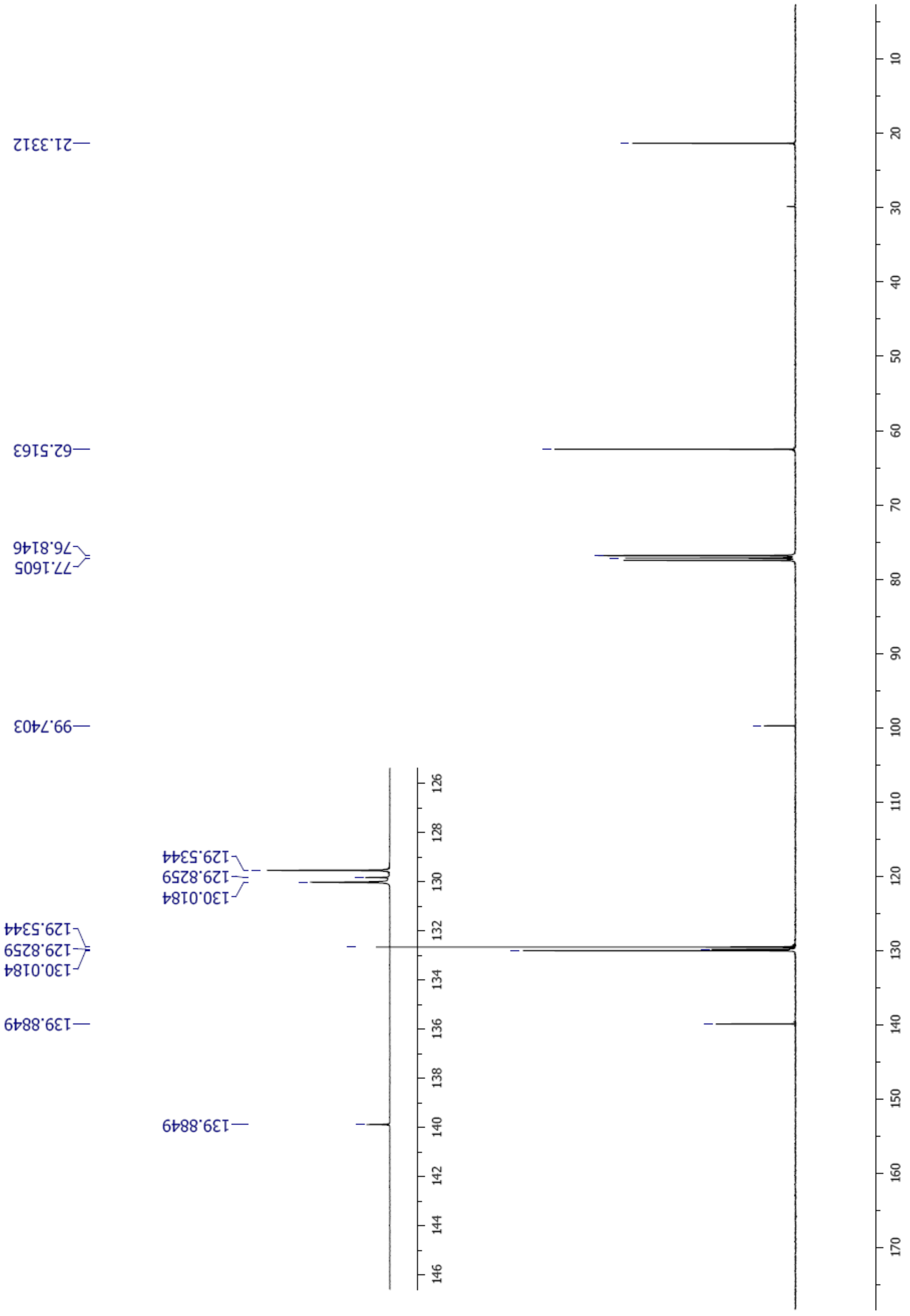
21.3312
62.5163
76.8146
77.1605
99.7403
129.5344
129.8259
130.0184
139.8849

^{1}H-^{1}H COSY (400 MHz)

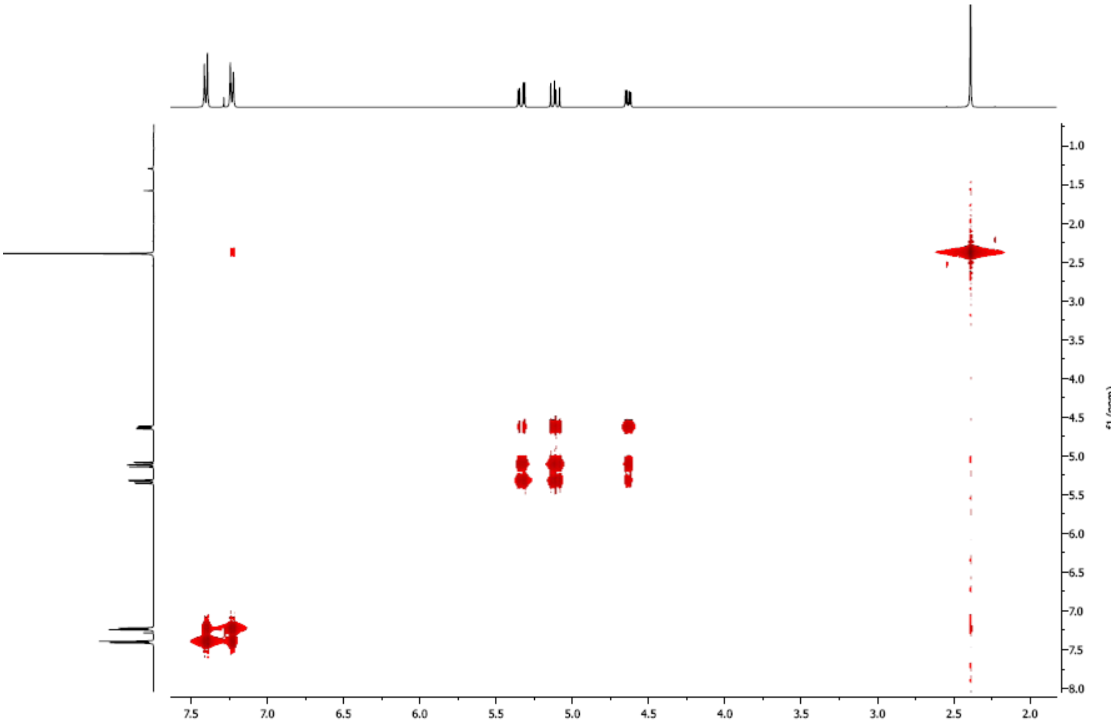

^{13}C DEPT 135 (100 MHz)

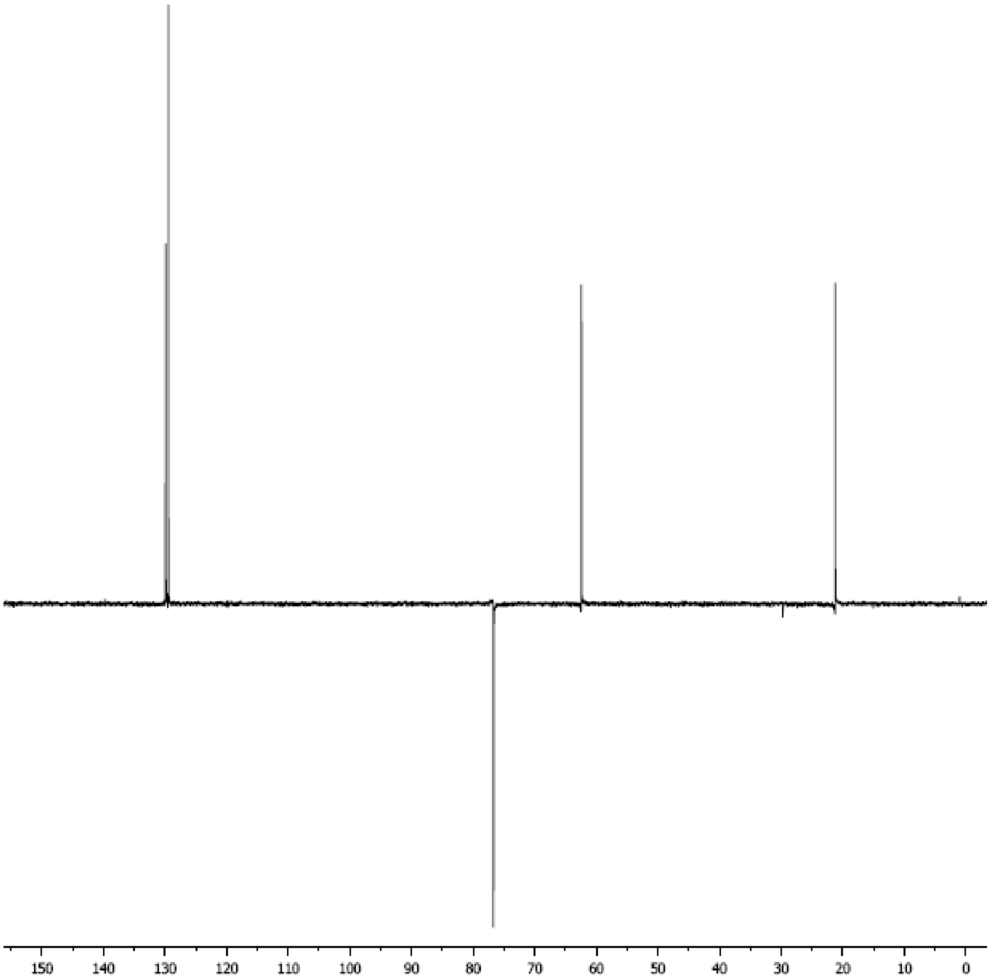

^{1}H-^{13}C HSQC (400 MHz)

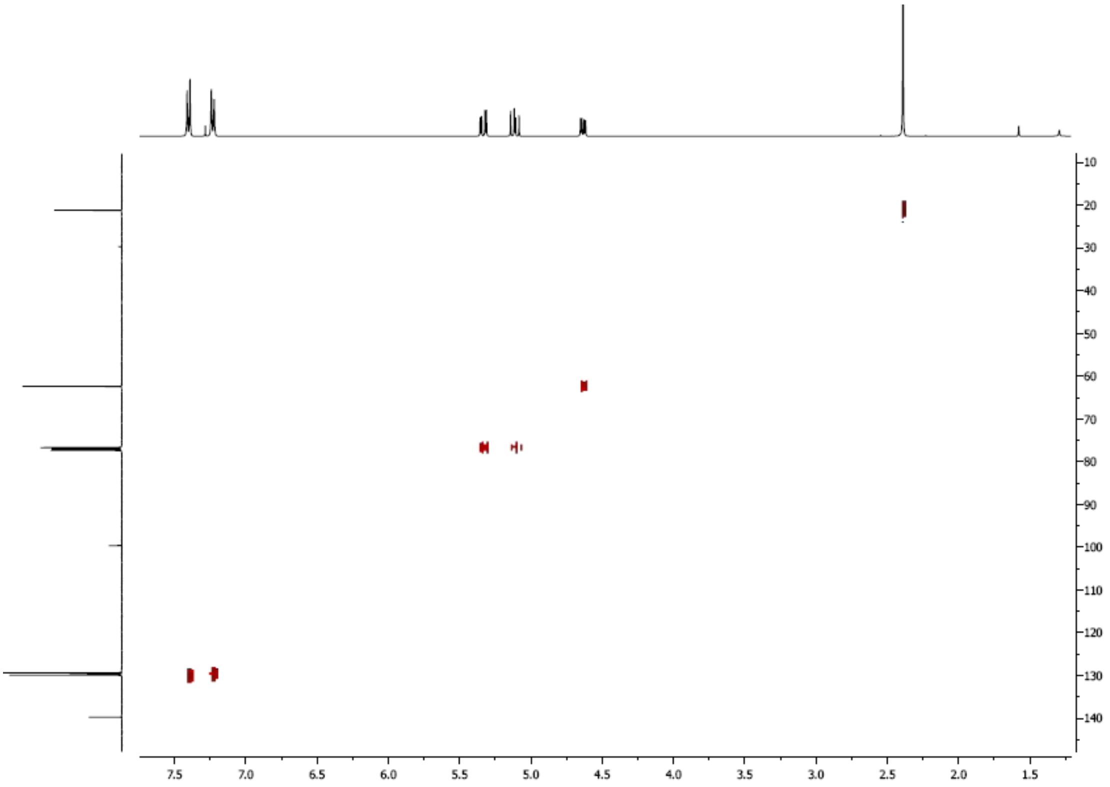

^{1}H-^{13}C HMBC (400 MHz)

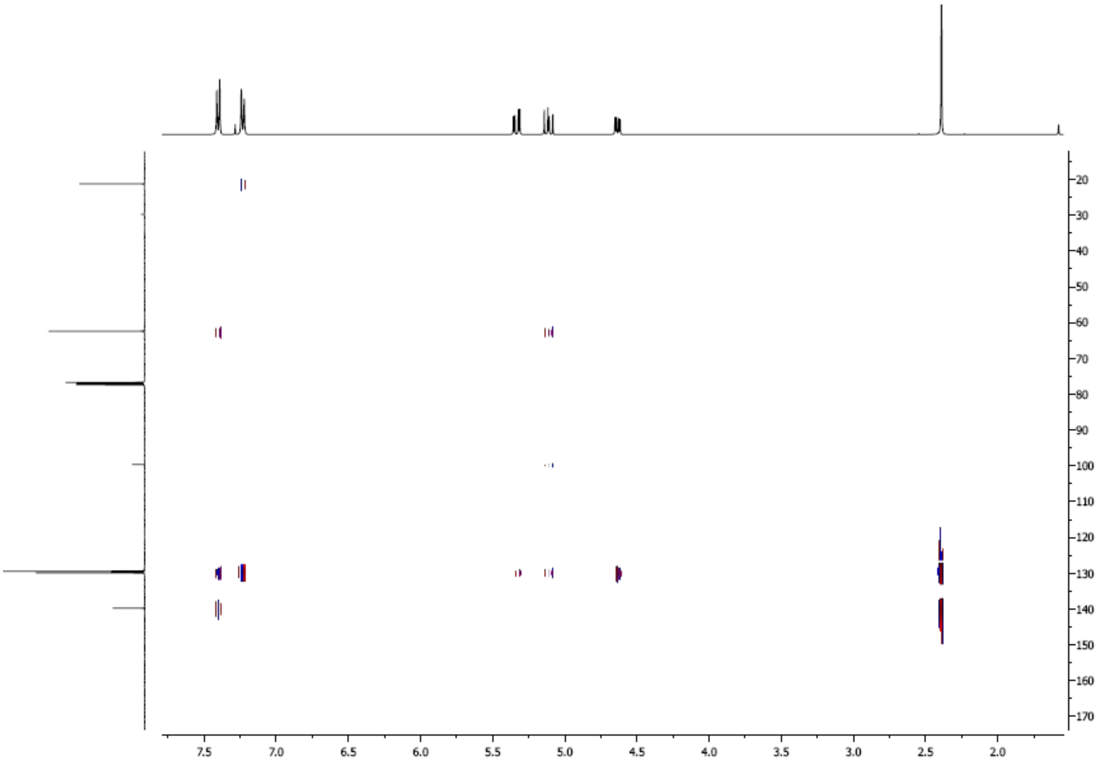

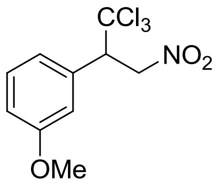
CCl3
NO2
OMe

2i

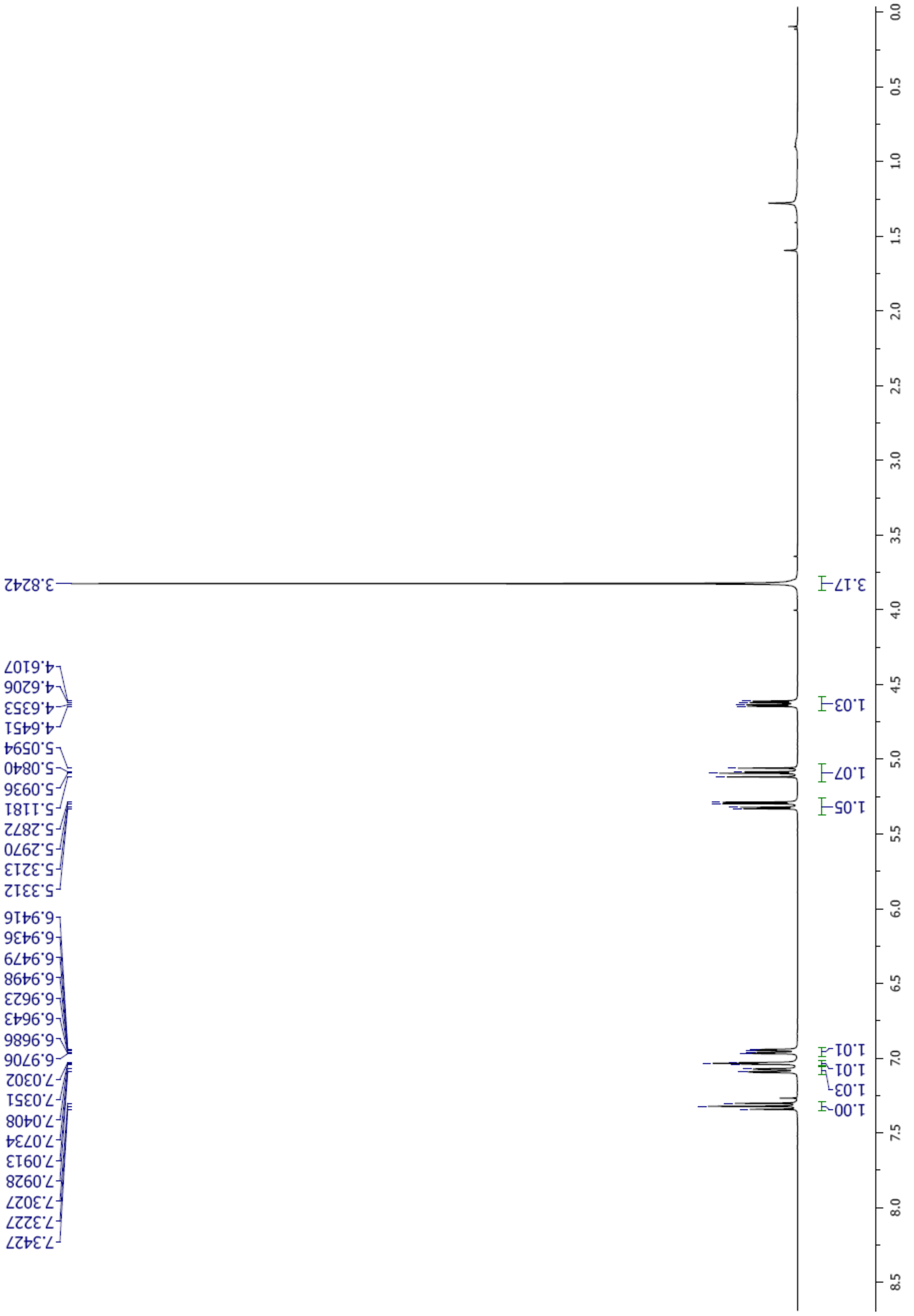
3.8242
4.6107
4.6206
4.6353
4.6451
5.0594
5.0840
5.0936
5.1181
5.2872
5.2970
5.3213
5.3312
6.9416
6.9436
6.9479
6.9498
6.9623
6.9643
6.9686
6.9706
7.0302
7.0351
7.0408
7.0734
7.0913
7.0928
7.3027
7.3227
7.3427
3.17
1.03
1.07
1.05
1.01
1.01
1.03
1.00
0.0
0.5
1.0
1.5
2.0
2.5
3.0
3.5
4.0
4.5
5.0
5.5
6.0
6.5
7.0
7.5
8.0
8.5

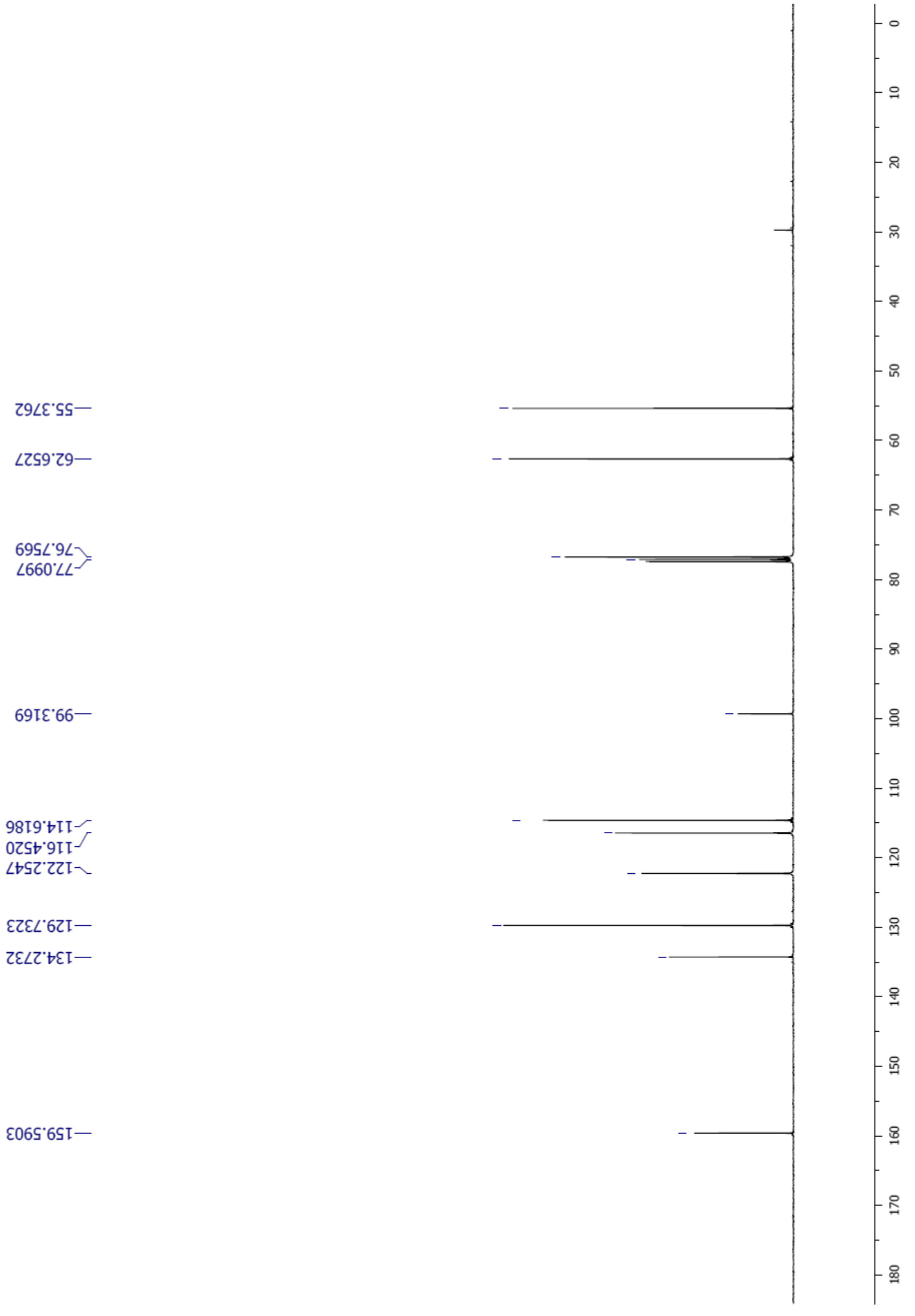
55.3762
62.6527
76.7569
77.0997
99.3169
114.6186
116.4520
122.2547
129.7323
134.2732
159.5903
0
10
20
30
40
50
60
70
80
90
100
110
120
130
140
150
160
170
180

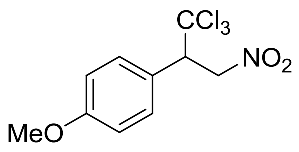
CCl3
NO2
MeO

2j

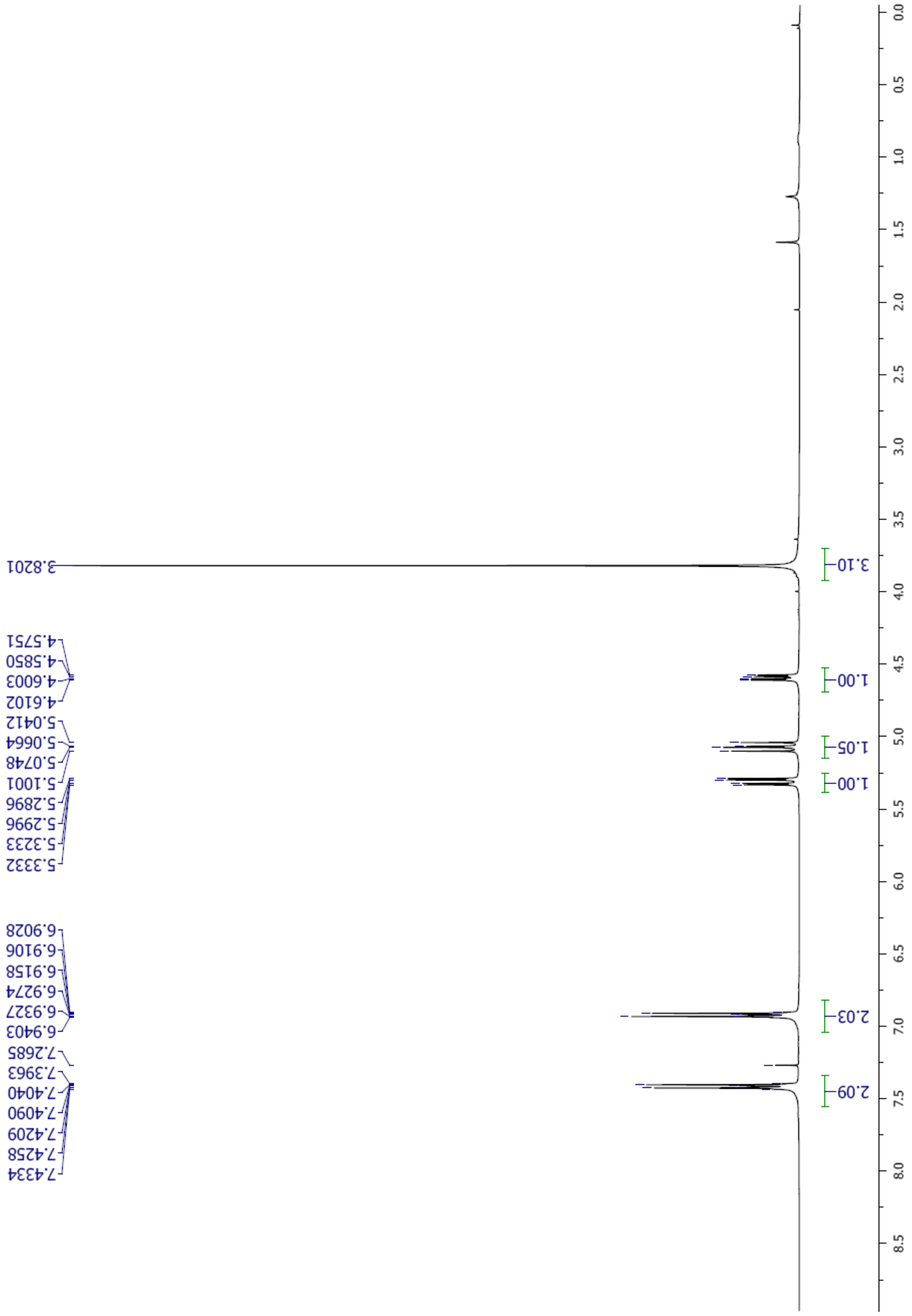
3.8201
4.5751
4.5850
4.6003
4.6102
5.0412
5.0664
5.0748
5.1001
5.2896
5.2996
5.3233
5.3332
6.9028
6.9106
6.9158
6.9274
6.9327
6.9403
7.2685
7.3963
7.4040
7.4090
7.4209
7.4258
7.4334
3.10
1.00
1.05
1.00
2.03
2.09
0.0
0.5
1.0
1.5
2.0
2.5
3.0
3.5
4.0
4.5
5.0
5.5
6.0
6.5
7.0
7.5
8.0
8.5

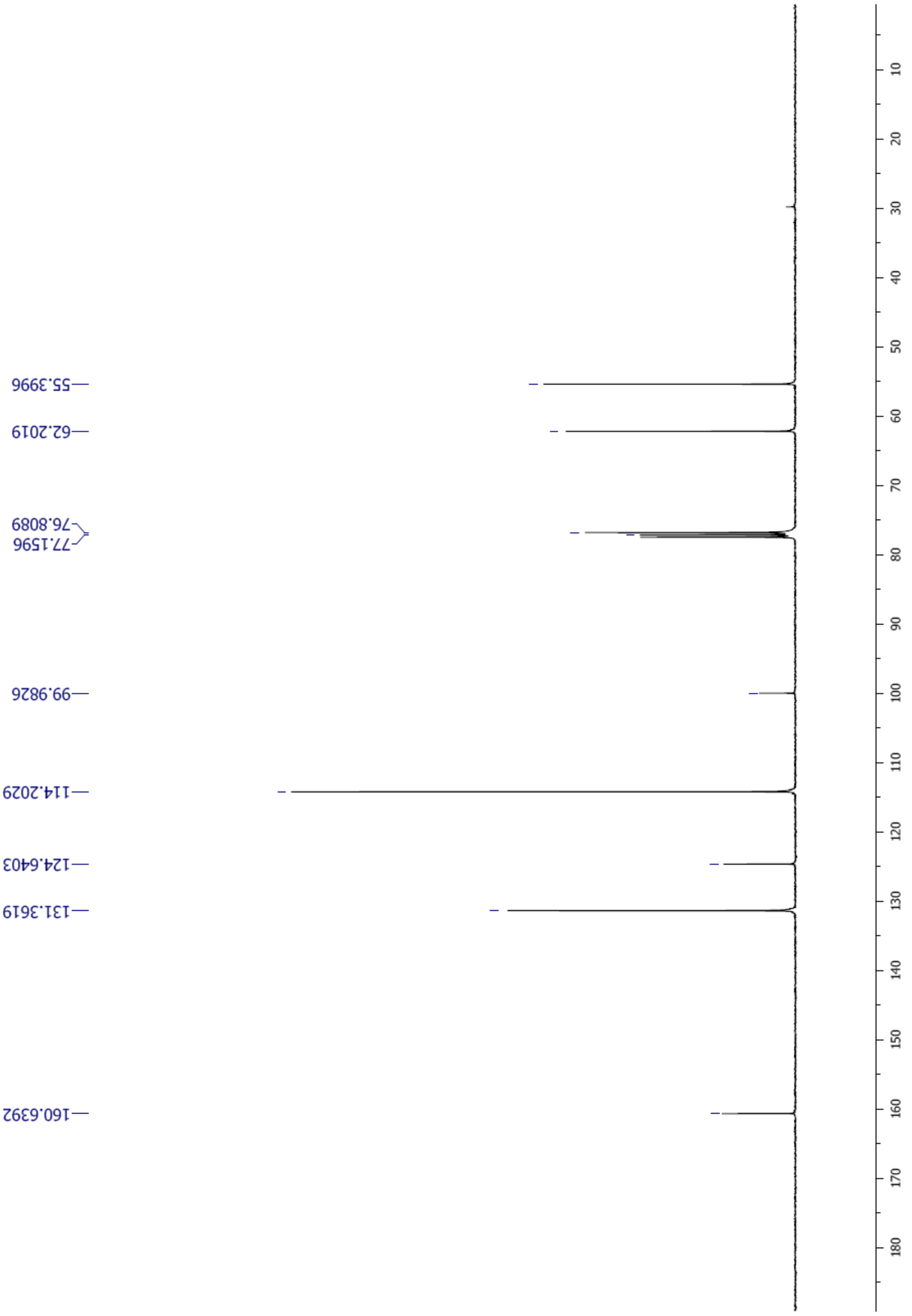
55.3996
62.2019
76.8089
77.1596
99.9826
114.2029
124.6403
131.3619
160.6392
10
20
30
40
50
60
70
80
90
100
110
120
130
140
150
160
170
180

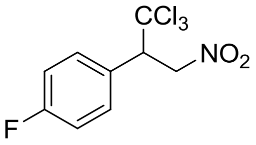
CCl3
NO2
F

2k

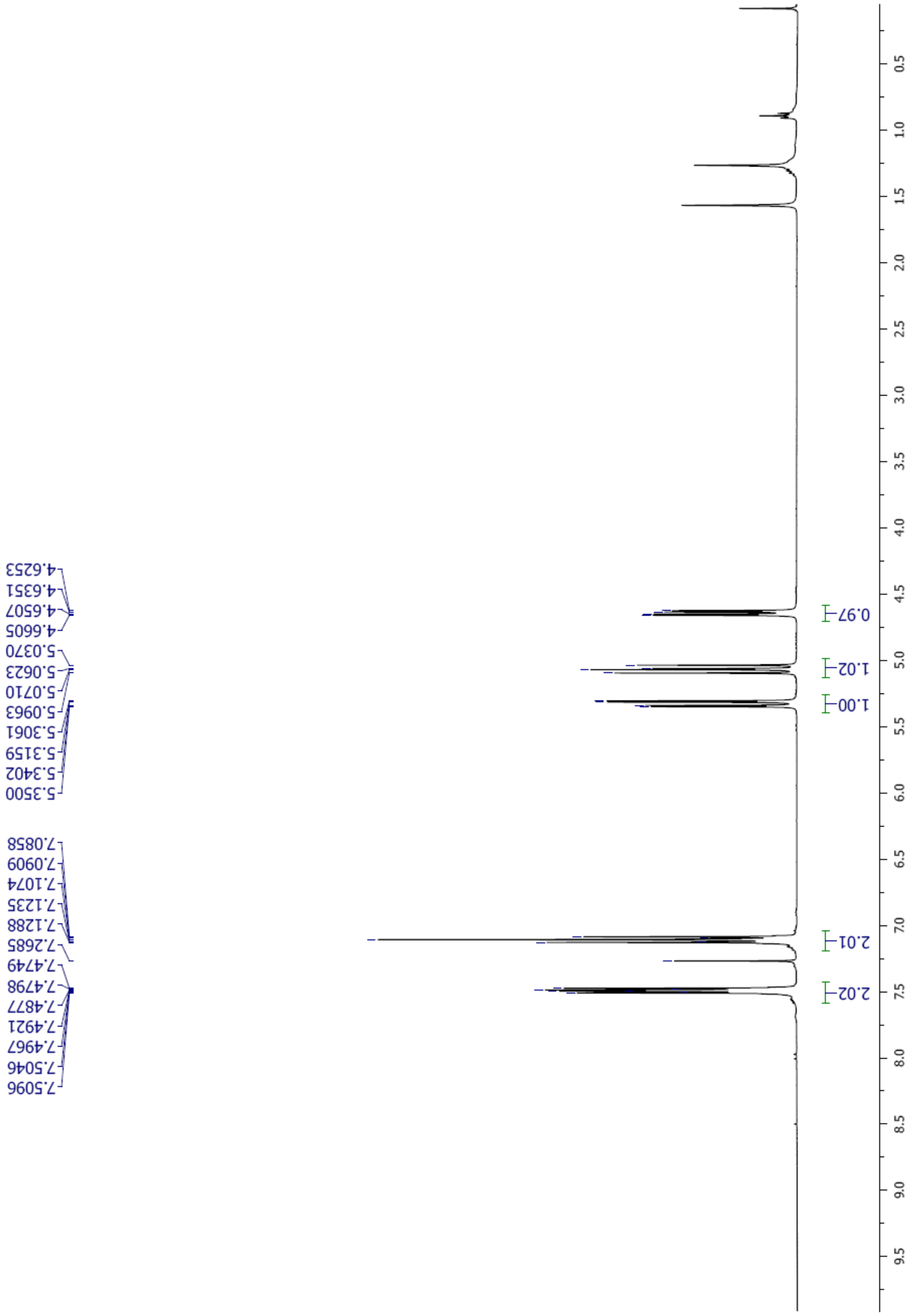
4.6253
4.6351
4.6507
4.6605
5.0370
5.0623
5.0710
5.0963
5.3061
5.3159
5.3402
5.3500
7.0858
7.0909
7.1074
7.1235
7.1288
7.2685
7.4749
7.4798
7.4877
7.4921
7.4967
7.5046
7.5096
0.97
1.02
1.00
2.01
2.02
0.5
1.0
1.5
2.0
2.5
3.0
3.5
4.0
4.5
5.0
5.5
6.0
6.5
7.0
7.5
8.0
8.5
9.0
9.5

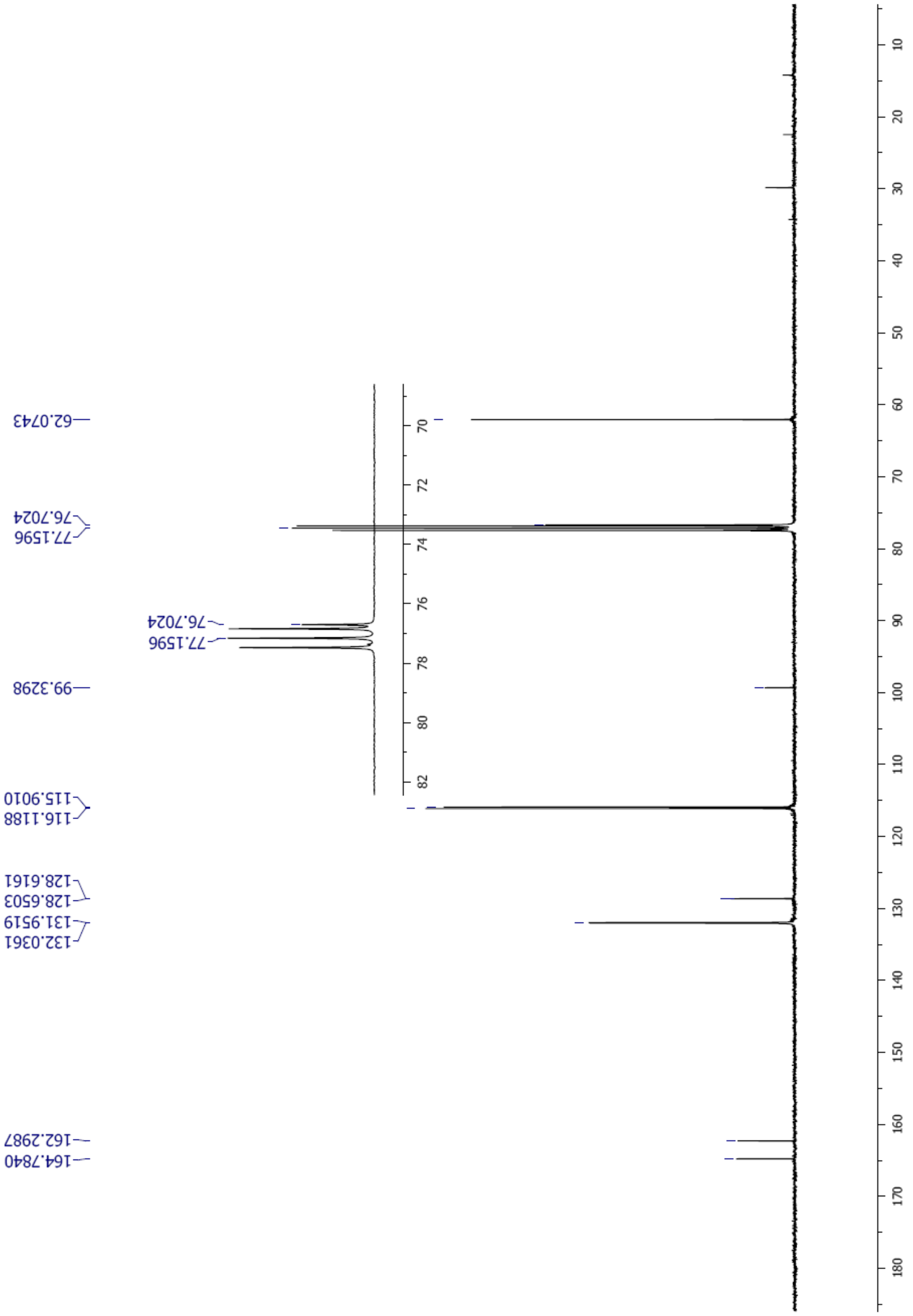
62.0743
76.7024
77.1596
99.3298
115.9010
116.1188
128.6161
128.6503
131.9519
132.0361
162.2987
164.7840

^{19}FNMR (376 MHz)

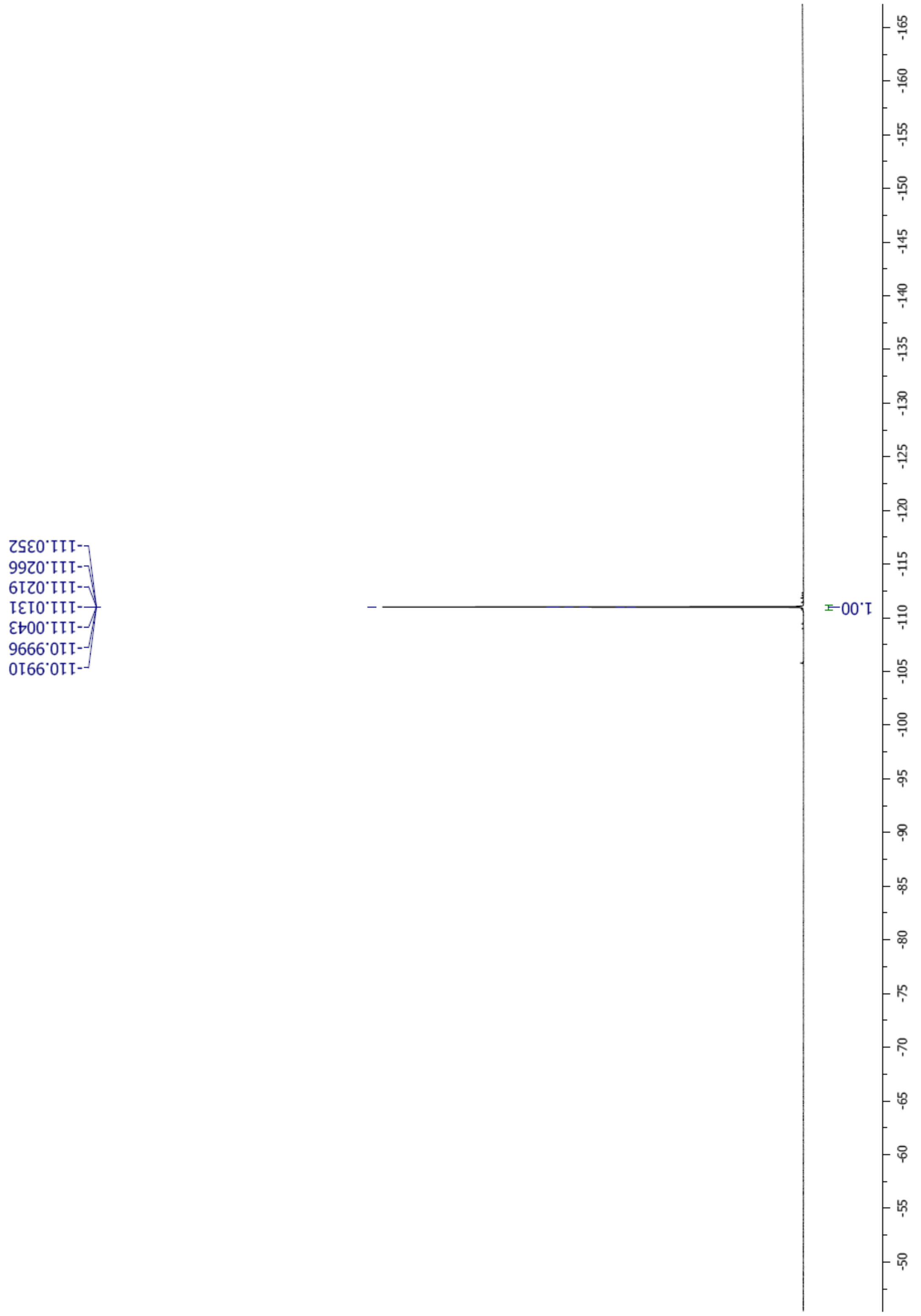

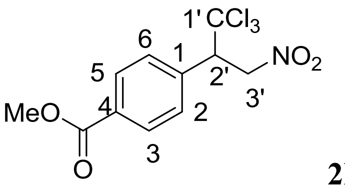

2l

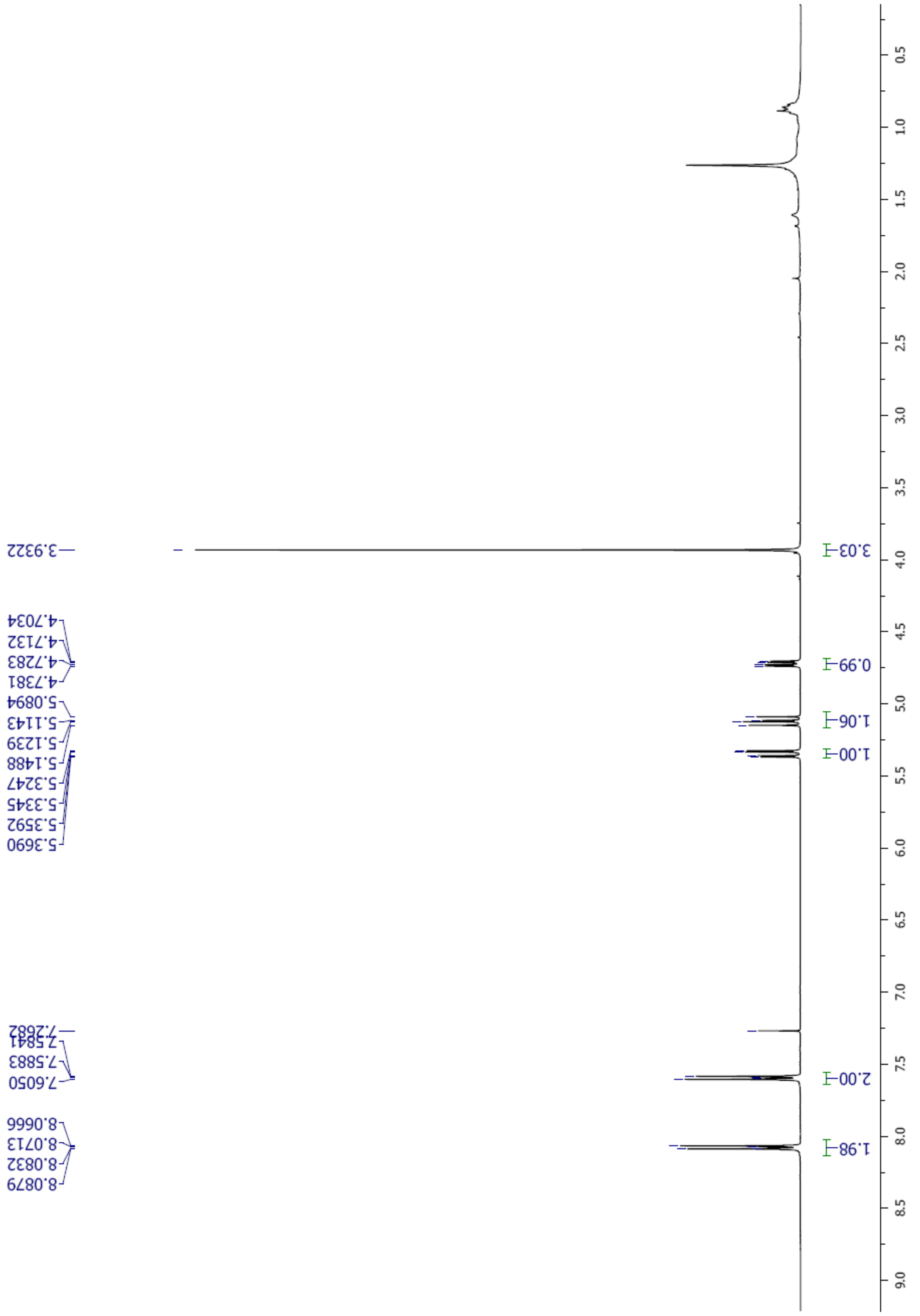

3.9322
4.7034
4.7132
4.7283
4.7381
5.0894
5.1143
5.1239
5.1488
5.3247
5.3345
5.3592
5.3690
7.2682
7.5841
7.5883
7.6050
8.0666
8.0713
8.0832
8.0879
3.03
0.99
1.06
1.00
2.00
1.98

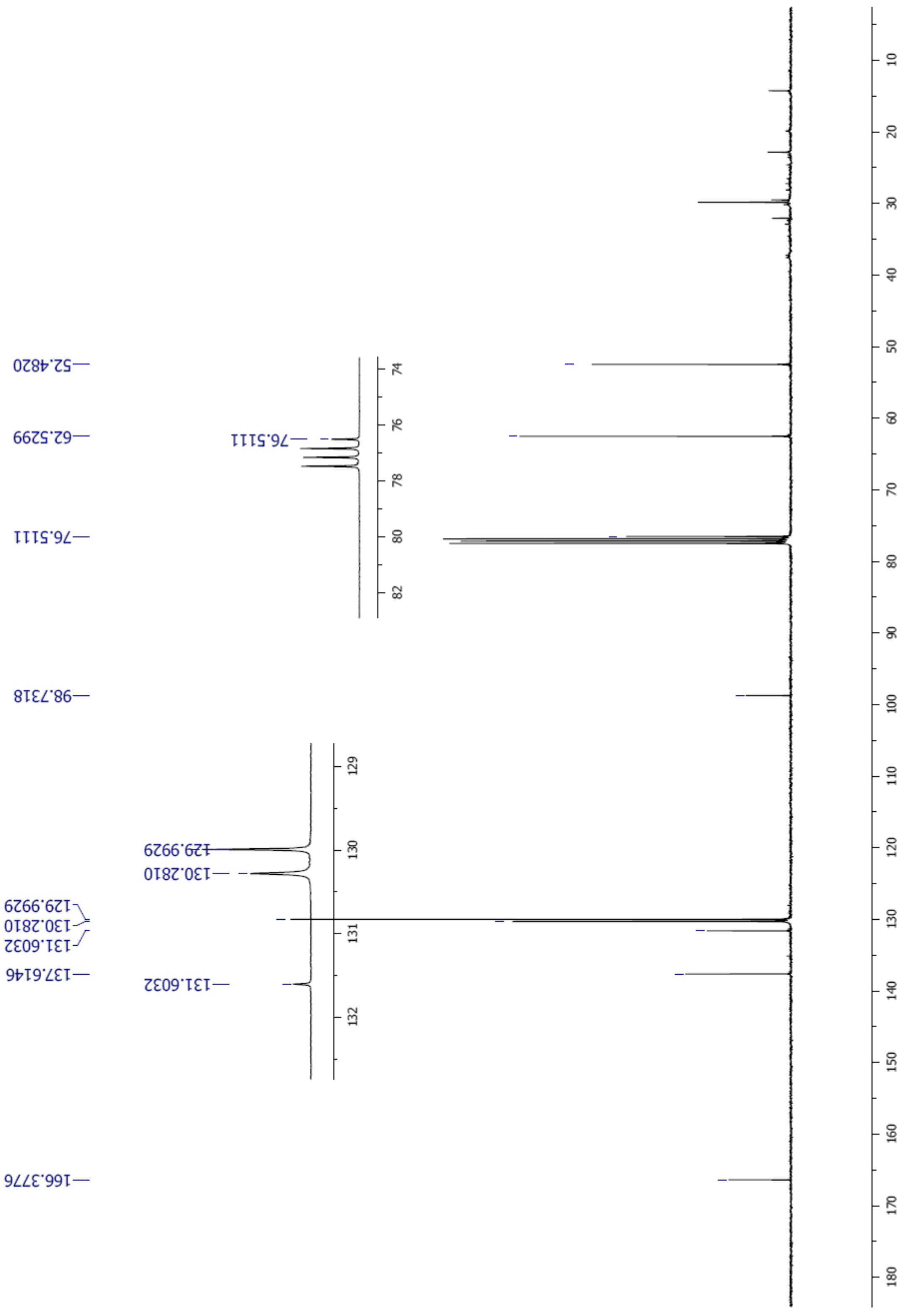
52.4820
62.5299
76.5111
98.7318
129.9929
130.2810
131.6032
137.6146
166.3776

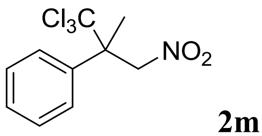
Cl3C
NO2

2m

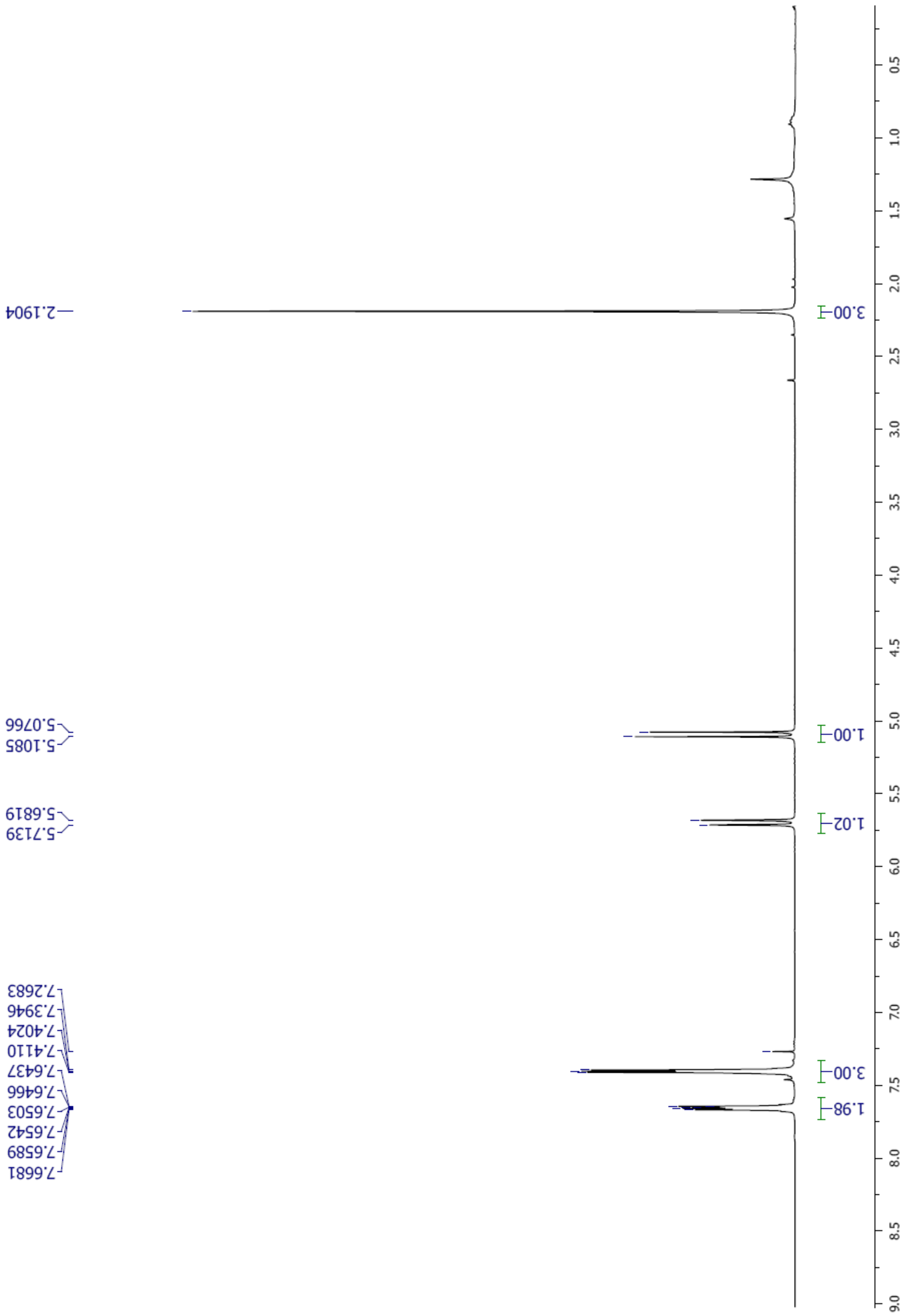
2.1904
5.0766
5.1085
5.6819
5.7139
7.2683
7.3946
7.4024
7.4110
7.6437
7.6466
7.6503
7.6542
7.6589
7.6681
3.00
1.00
1.02
3.00
1.98
0.5
1.0
1.5
2.0
2.5
3.0
3.5
4.0
4.5
5.0
5.5
6.0
6.5
7.0
7.5
8.0
8.5
9.0

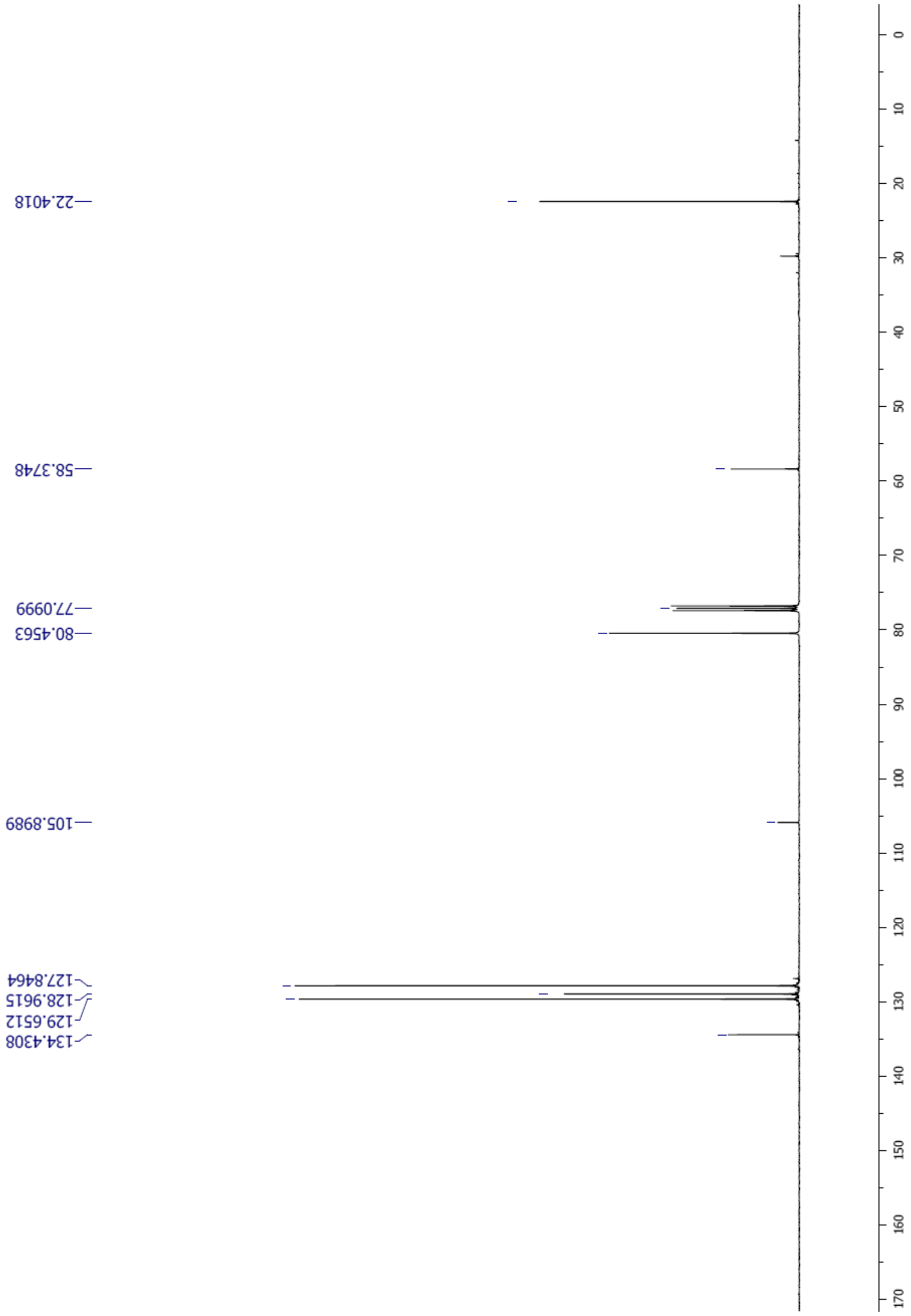
22.4018
58.3748
77.0999
80.4563
105.8989
127.8464
128.9615
129.6512
134.4308

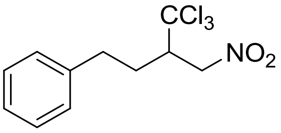
CCl3
NO2

2n

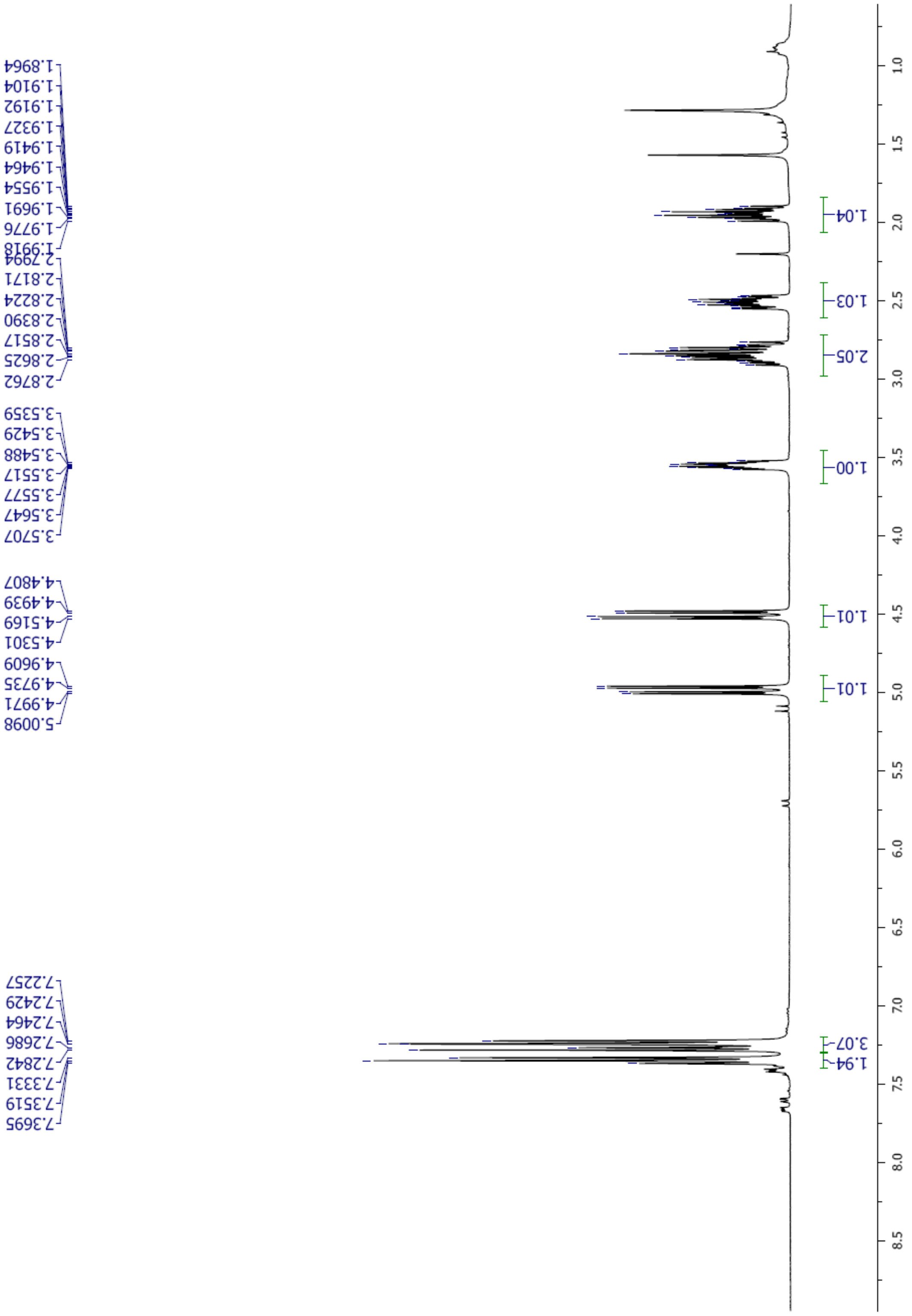
1.8964
1.9104
1.9192
1.9327
1.9419
1.9464
1.9554
1.9691
1.9776
1.9918
2.7994
2.8171
2.8224
2.8390
2.8517
2.8625
2.8762
3.5359
3.5429
3.5488
3.5517
3.5577
3.5647
3.5707
4.4807
4.4939
4.5169
4.5301
4.9609
4.9735
4.9971
5.0098
7.2257
7.2429
7.2464
7.2686
7.2842
7.3331
7.3519
7.3695
1.04
1.03
2.05
1.00
1.01
1.01
3.07
1.94
1.0
1.5
2.0
2.5
3.0
3.5
4.0
4.5
5.0
5.5
6.0
6.5
7.0
7.5
8.0
8.5

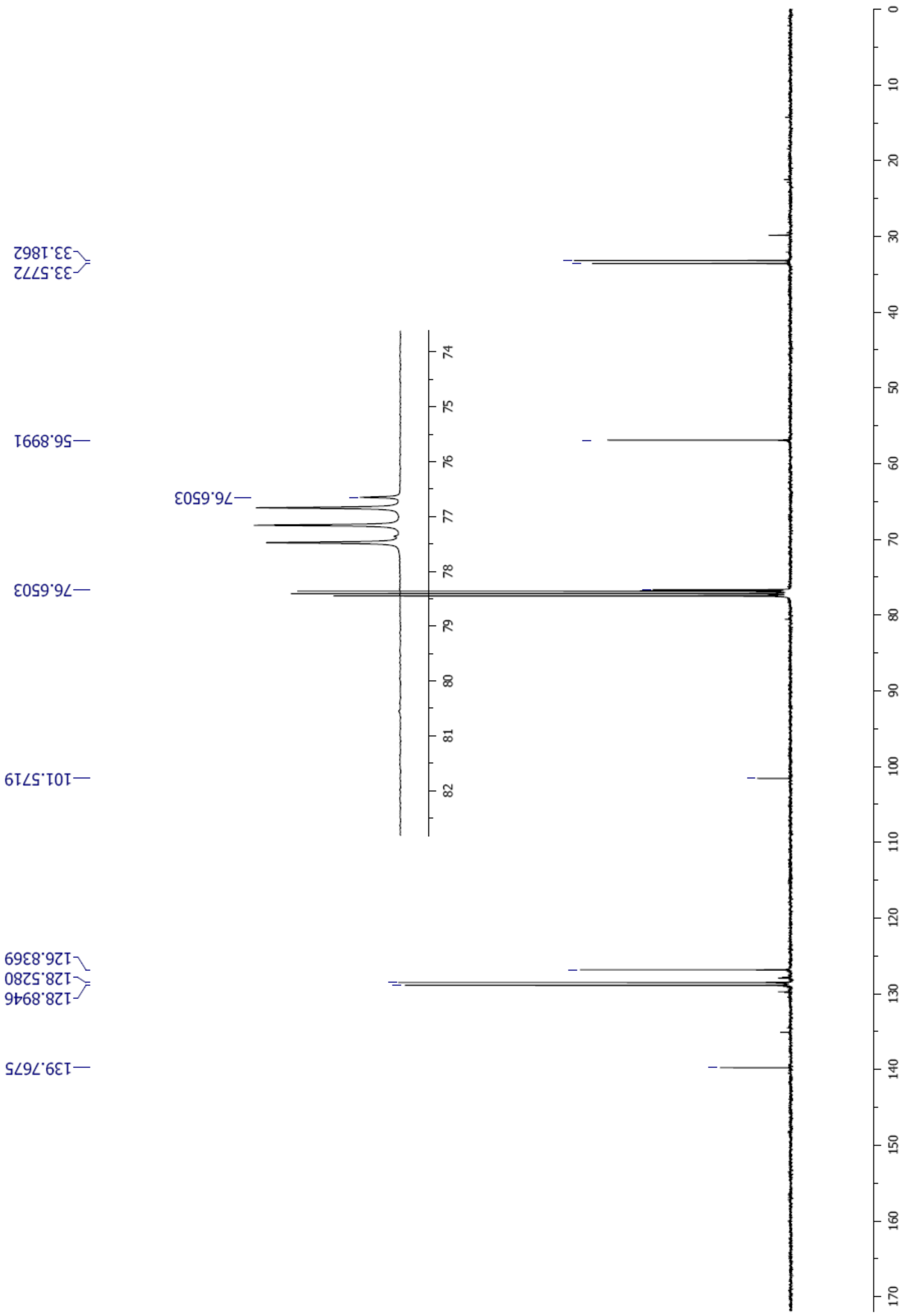
33.1862
33.5772
56.8991
76.6503
76.6503
101.5719
126.8369
128.5280
128.8946
139.7675

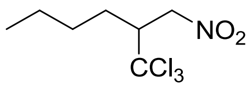
NO2
CCl3

2o

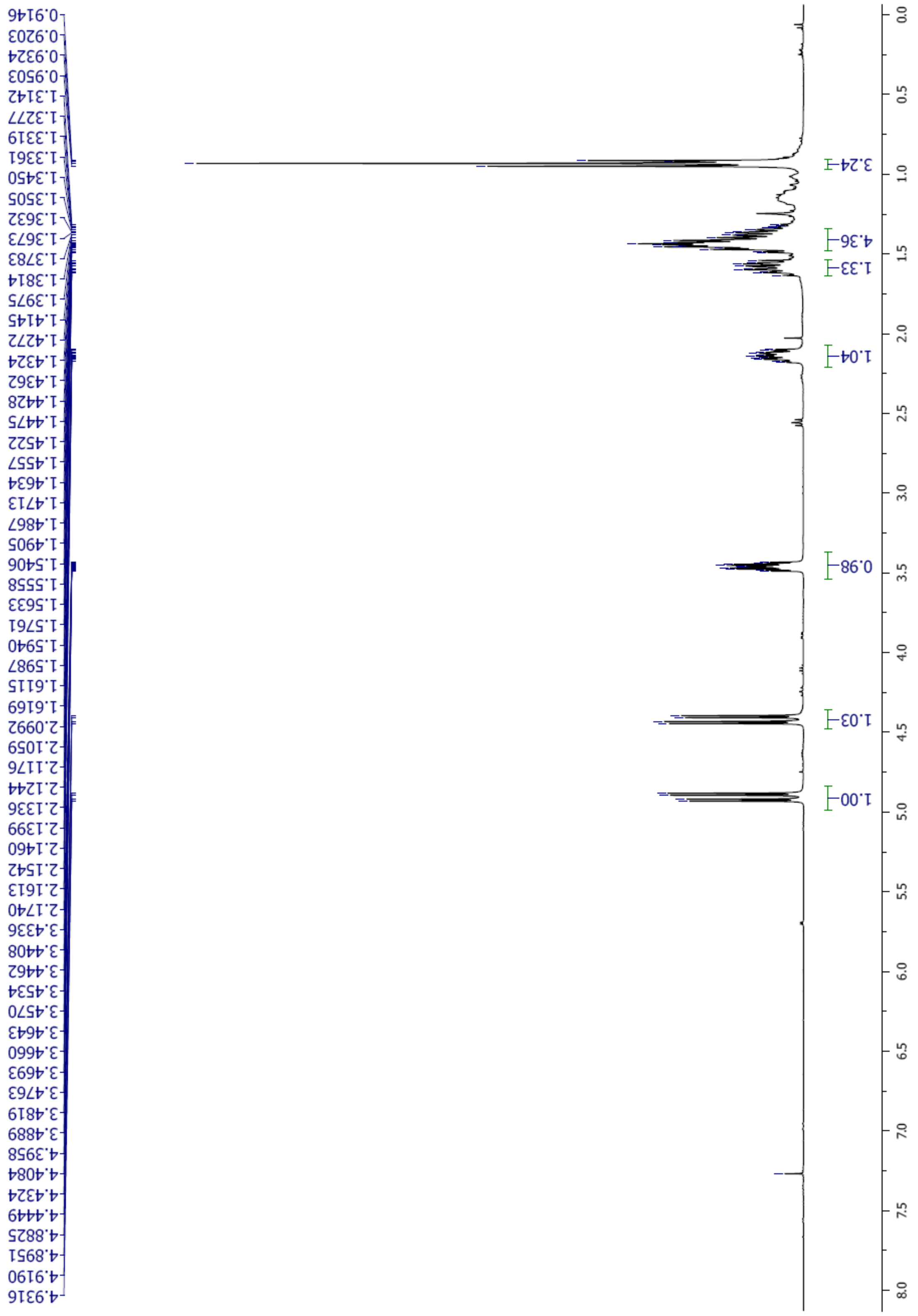
0.9146
0.9203
0.9324
0.9503
1.3142
1.3277
1.3319
1.3361
1.3450
1.3505
1.3632
1.3673
1.3783
1.3814
1.3975
1.4145
1.4272
1.4324
1.4362
1.4428
1.4475
1.4522
1.4557
1.4634
1.4713
1.4867
1.4905
1.5406
1.5558
1.5633
1.5761
1.5940
1.5987
1.6115
1.6169
2.0992
2.1059
2.1176
2.1244
2.1336
2.1399
2.1460
2.1542
2.1613
2.1740
3.4336
3.4408
3.4462
3.4534
3.4570
3.4643
3.4660
3.4693
3.4763
3.4819
3.4889
4.3958
4.4084
4.4324
4.4449
4.8825
4.8951
4.9190
4.9316
3.24
4.36
1.33
1.04
0.98
1.03
1.00
0.0
0.5
1.0
1.5
2.0
2.5
3.0
3.5
4.0
4.5
5.0
5.5
6.0
6.5
7.0
7.5
8.0

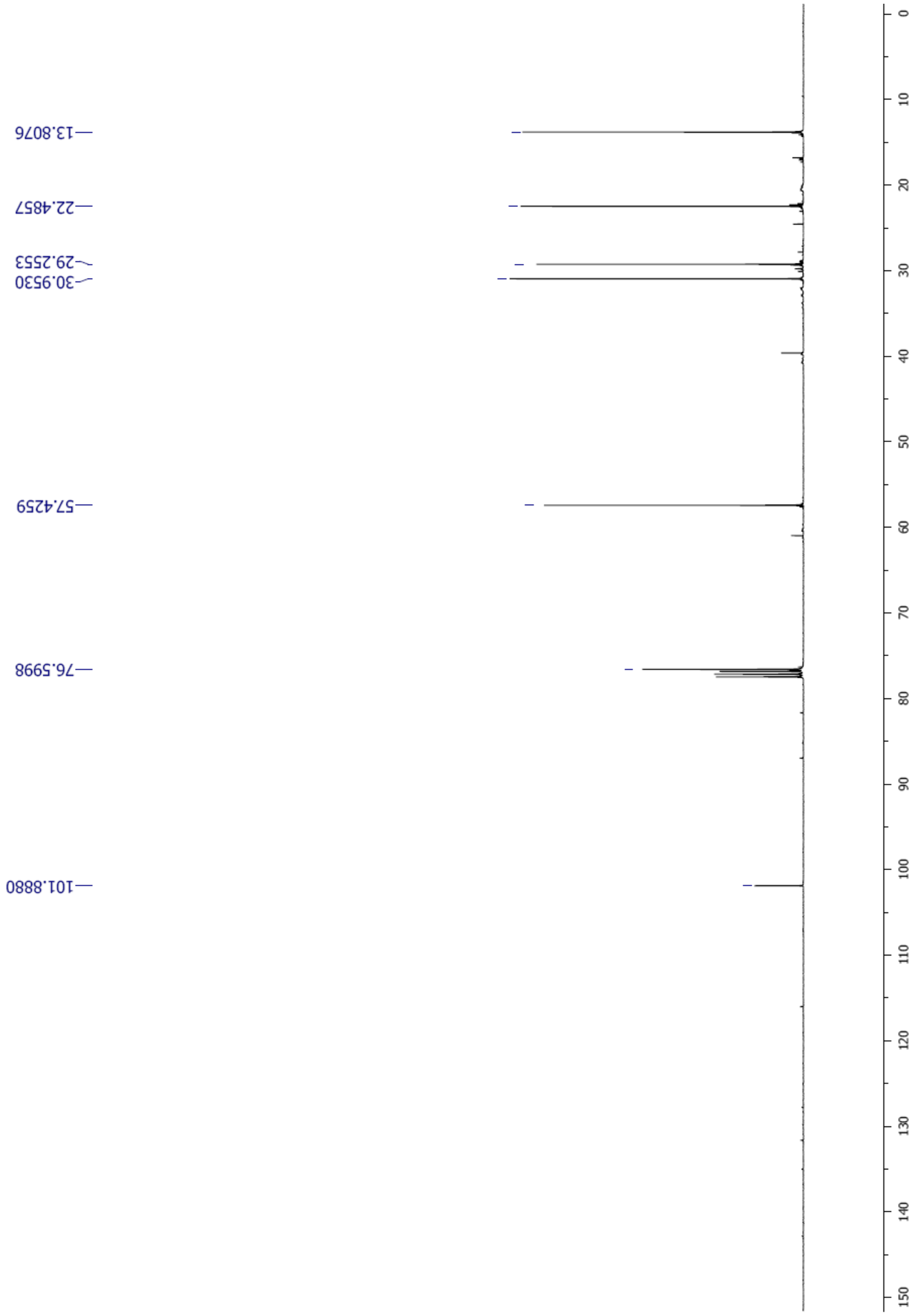
13.8076
22.4857
29.2553
30.9530
57.4259
76.5998
101.8880
0
10
20
30
40
50
60
70
80
90
100
110
120
130
140
150

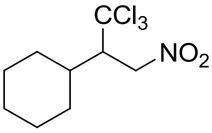
CCl_3
NO_2

2p

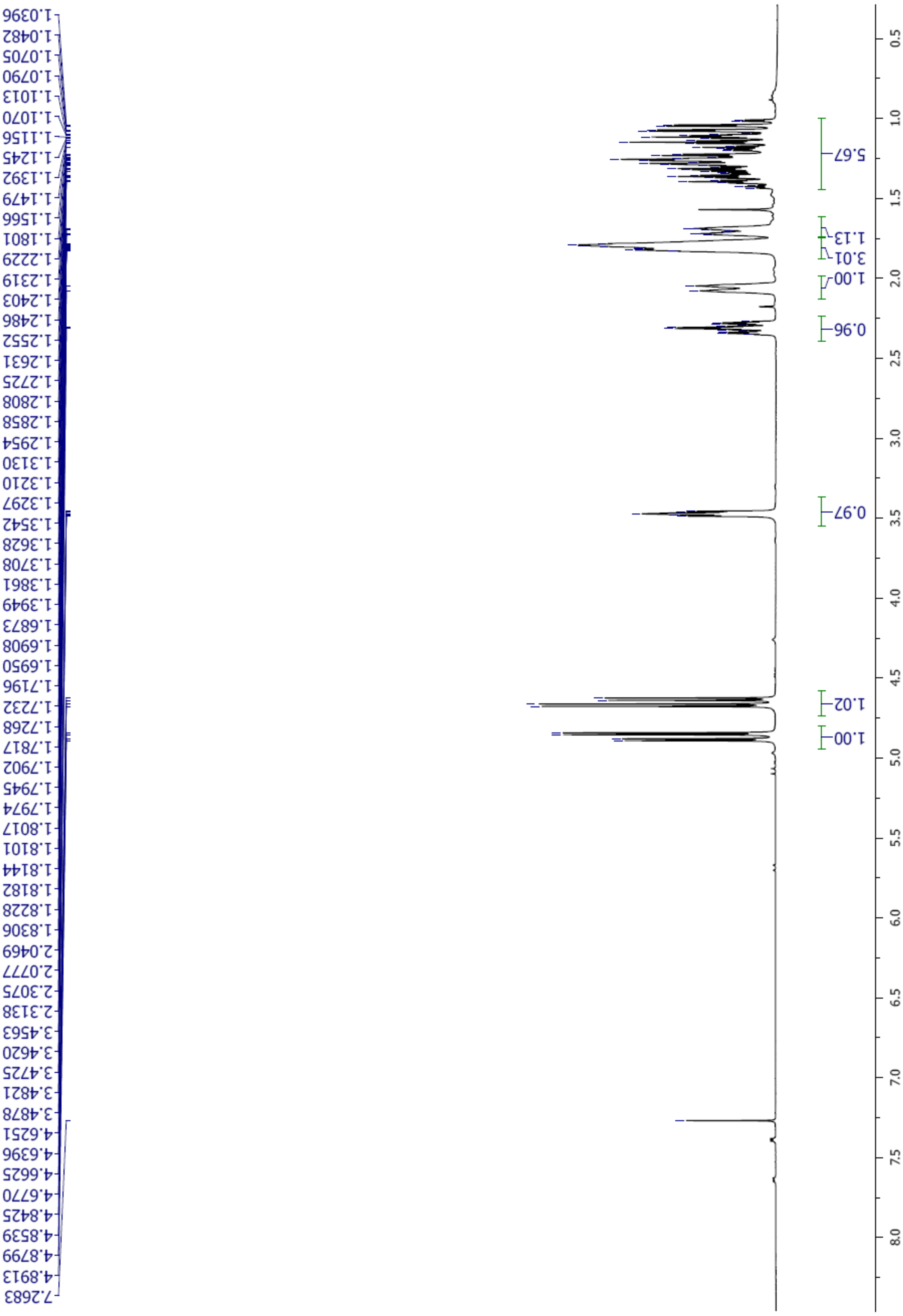
1.0396
1.0482
1.0705
1.0790
1.1013
1.1070
1.1156
1.1245
1.1392
1.1479
1.1566
1.1801
1.2229
1.2319
1.2403
1.2486
1.2552
1.2631
1.2725
1.2808
1.2858
1.2954
1.3130
1.3210
1.3297
1.3542
1.3628
1.3708
1.3861
1.3949
1.6873
1.6908
1.6950
1.7196
1.7232
1.7268
1.7817
1.7902
1.7945
1.7974
1.8017
1.8101
1.8144
1.8182
1.8228
1.8306
2.0469
2.0777
2.3075
2.3138
3.4563
3.4620
3.4725
3.4821
3.4878
4.6251
4.6396
4.6625
4.6770
4.8425
4.8539
4.8799
4.8913
7.2683
5.67
1.13
3.01
1.00
0.96
0.97
1.02
1.00
0.5
1.0
1.5
2.0
2.5
3.0
3.5
4.0
4.5
5.0
5.5
6.0
6.5
7.0
7.5
8.0

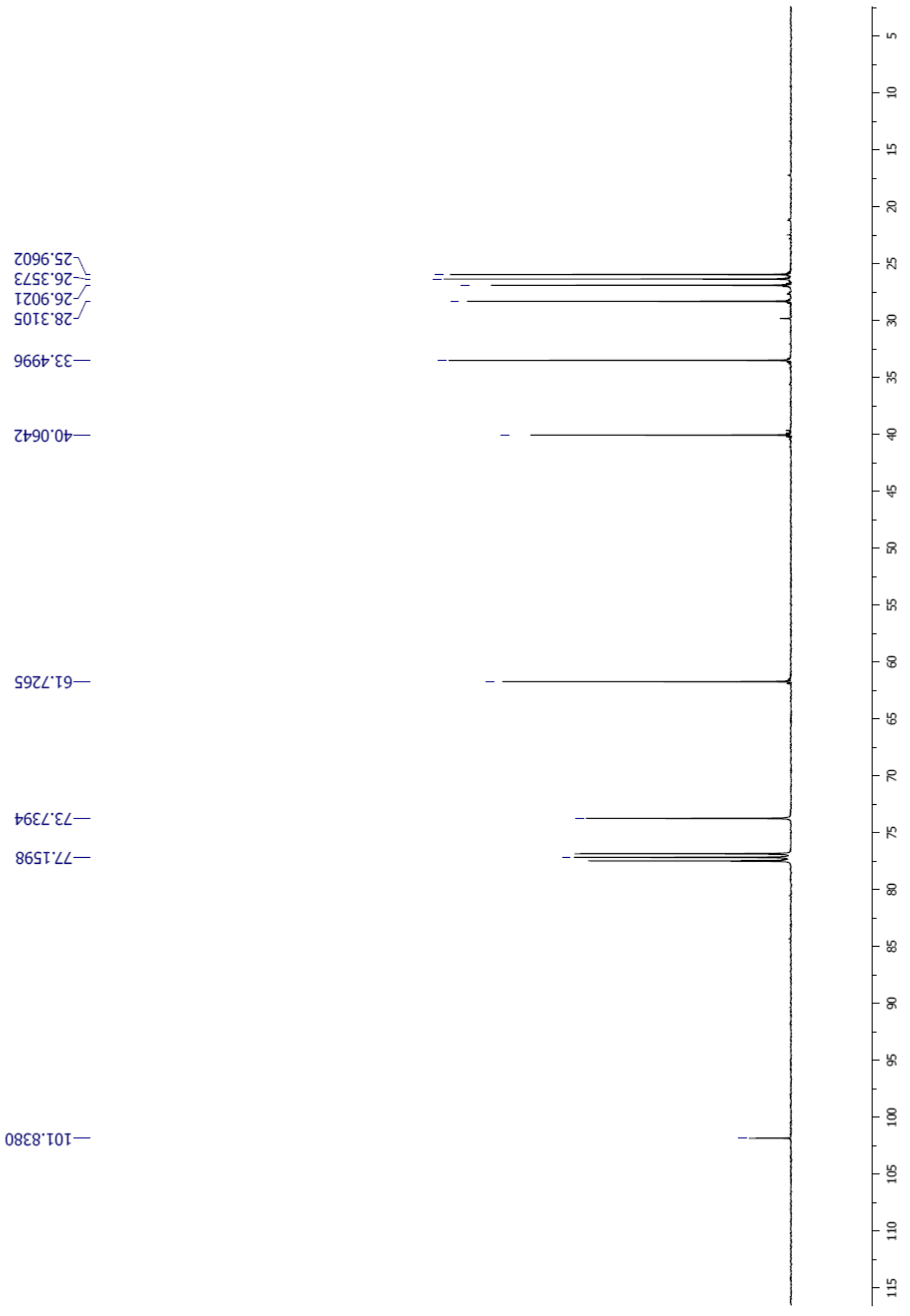
25.9602
26.3573
26.9021
28.3105
33.4996
40.0642
61.7265
73.7394
77.1598
101.8380

2q

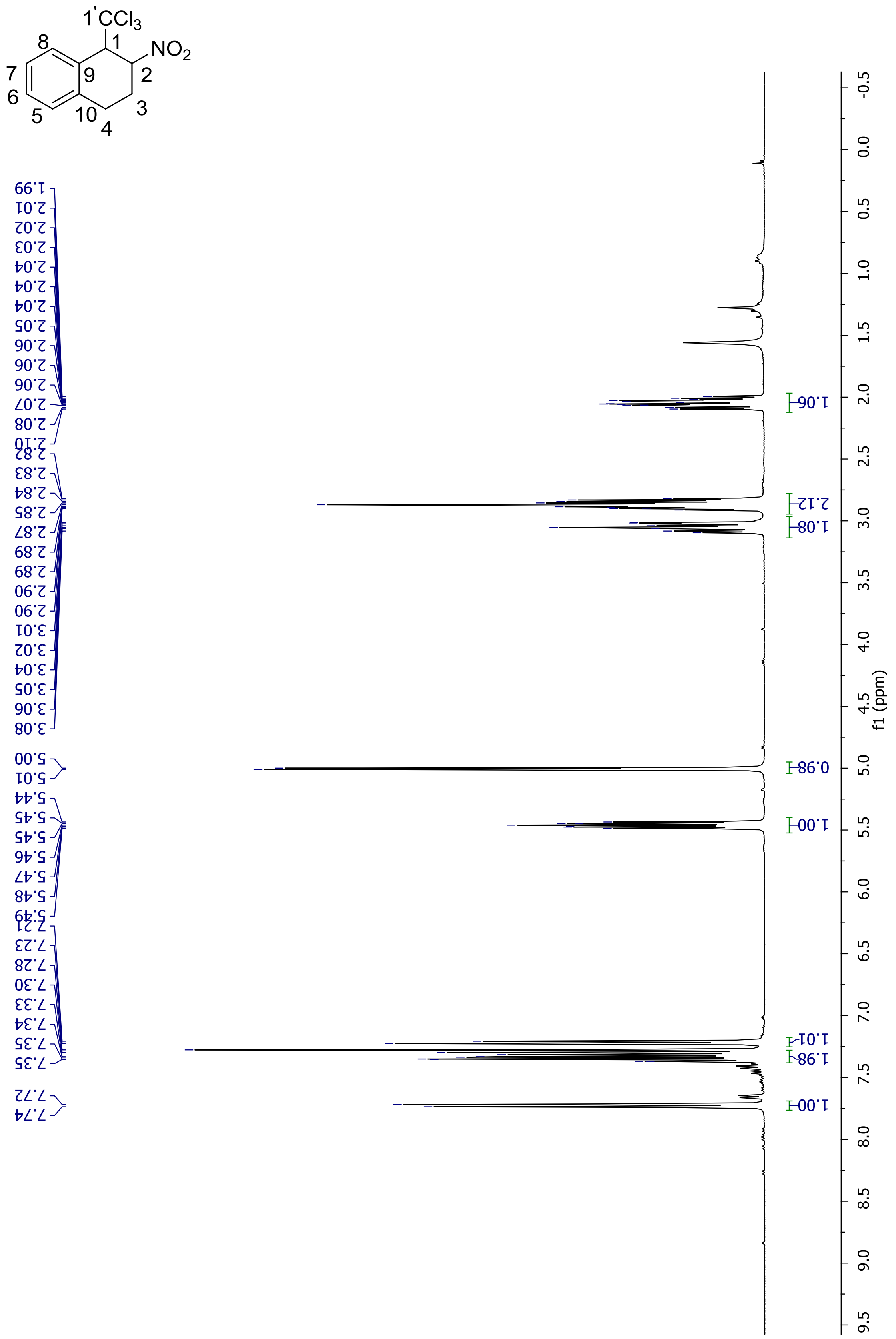

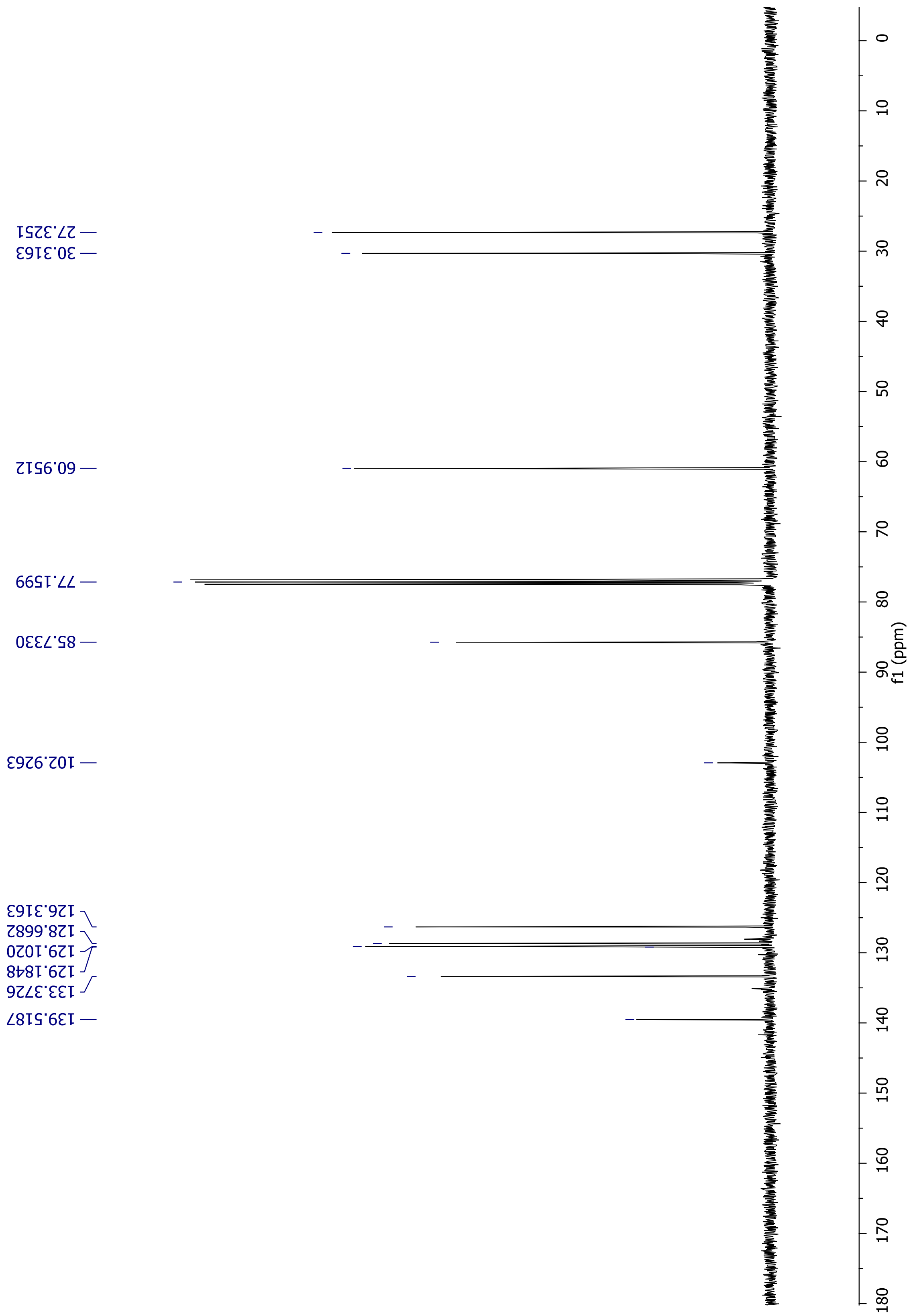

27.3251
30.3163
60.9512
77.1599
85.7330
102.9263
126.3163
128.6682
129.1020
129.1848
133.3726
139.5187
0
10
20
30
40
50
60
70
80
90
100
110
120
130
140
150
160
170
180
f1 (ppm)

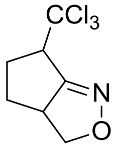
CCl3
N
O

2r

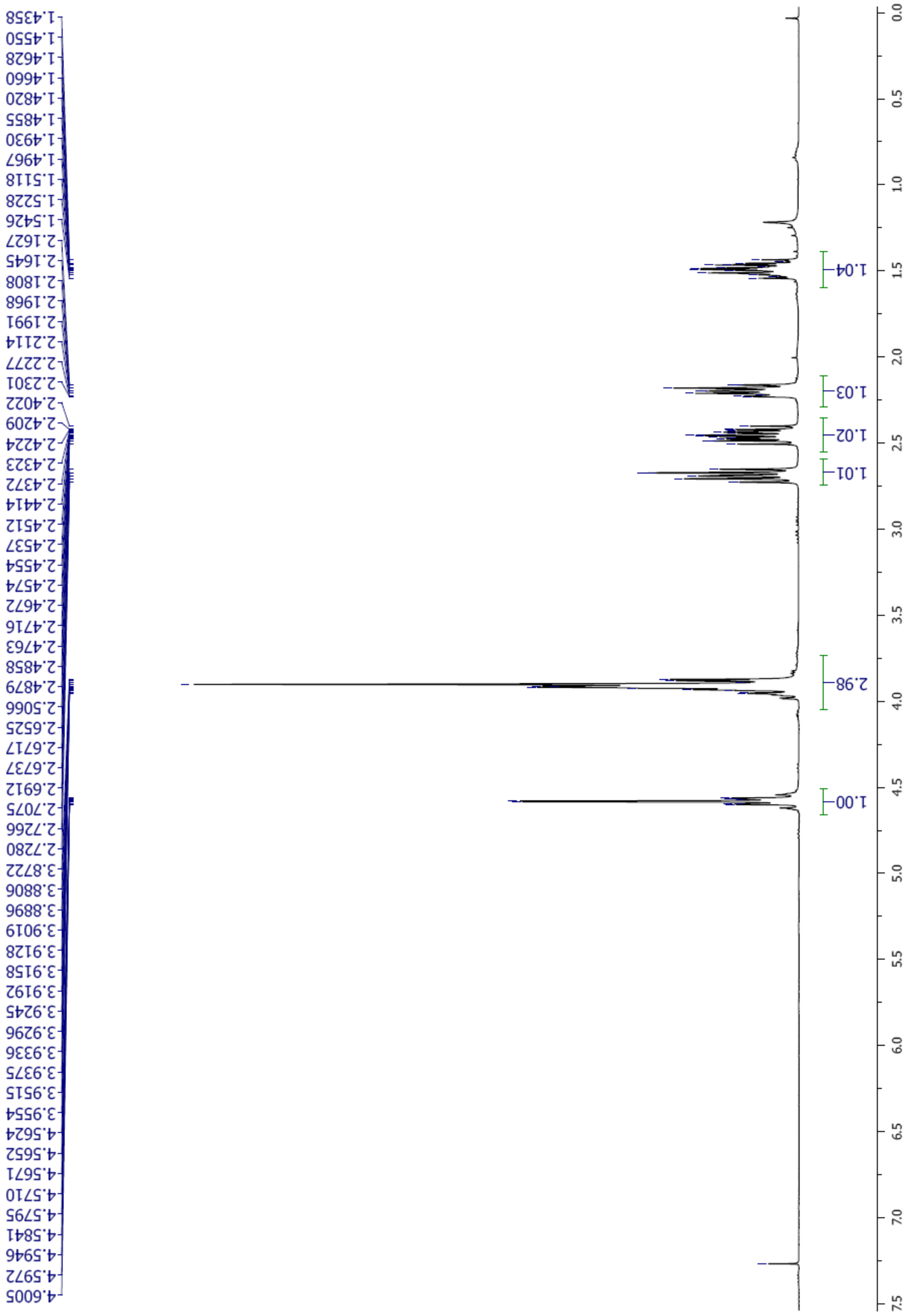
1.4358
1.4550
1.4628
1.4660
1.4820
1.4855
1.4930
1.4967
1.5118
1.5228
1.5426
2.1627
2.1645
2.1808
2.1968
2.1991
2.2114
2.2277
2.2301
2.4022
2.4209
2.4224
2.4323
2.4372
2.4414
2.4512
2.4537
2.4554
2.4574
2.4672
2.4716
2.4763
2.4858
2.4879
2.5066
2.6525
2.6717
2.6737
2.6912
2.7075
2.7266
2.7280
3.8722
3.8806
3.8896
3.9019
3.9128
3.9158
3.9192
3.9245
3.9296
3.9336
3.9375
3.9515
3.9554
4.5624
4.5652
4.5671
4.5710
4.5795
4.5841
4.5946
4.5972
4.6005
1.04
1.03
1.02
1.01
2.98
1.00
0.0
0.5
1.0
1.5
2.0
2.5
3.0
3.5
4.0
4.5
5.0
5.5
6.0
6.5
7.0
7.5

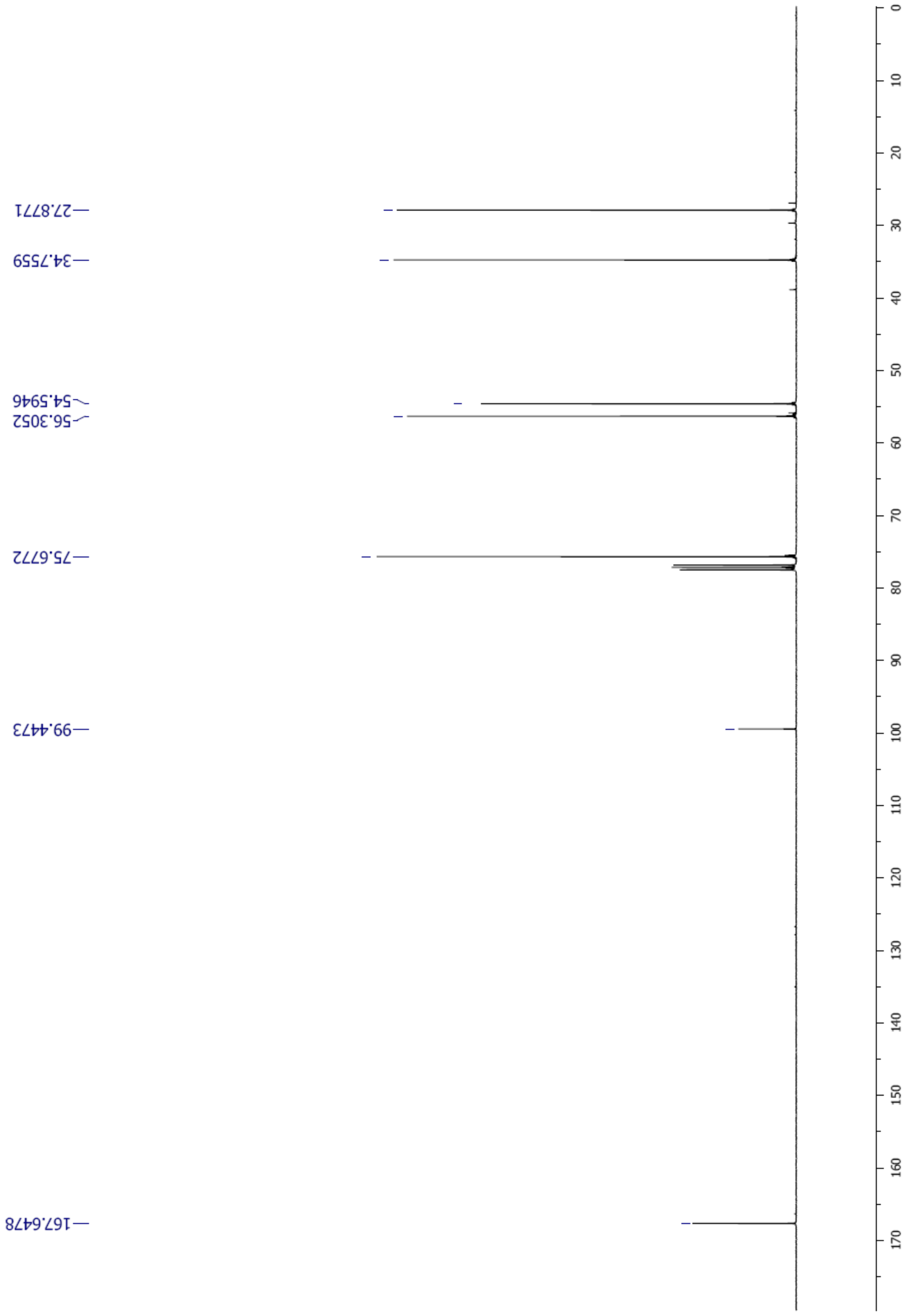
27.8771
34.7559
54.5946
56.3052
75.6772
99.4473
167.6478
0
10
20
30
40
50
60
70
80
90
100
110
120
130
140
150
160
170

^1H-^1H COSY (400 MHz)

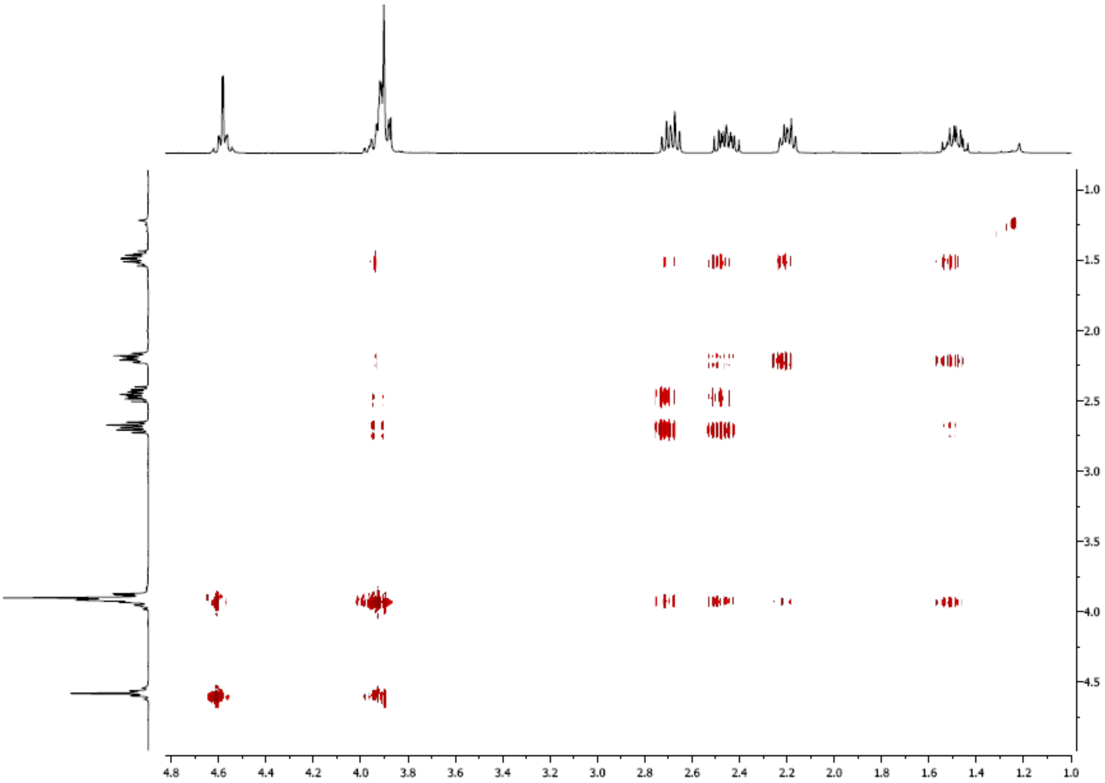

DEPT 135 C (100 MHz)

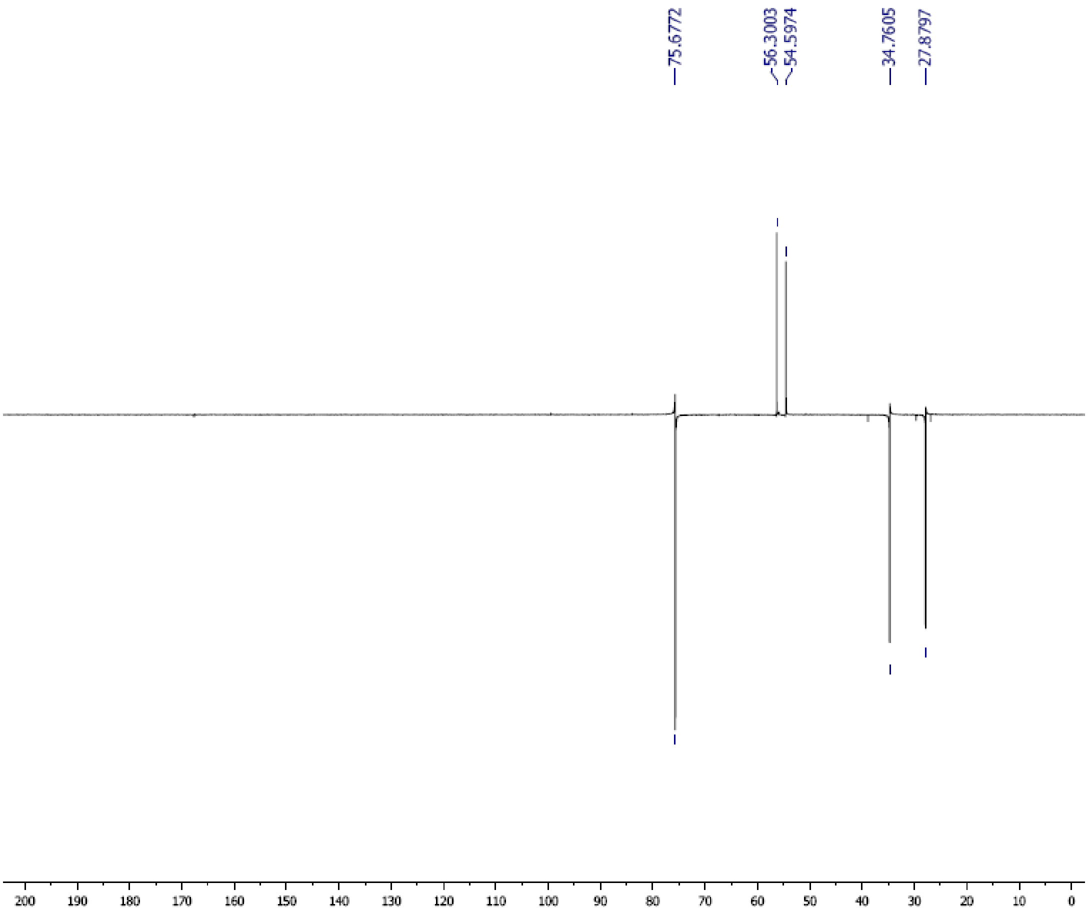

^{1}H-^{13}C HSQC (400MHz)

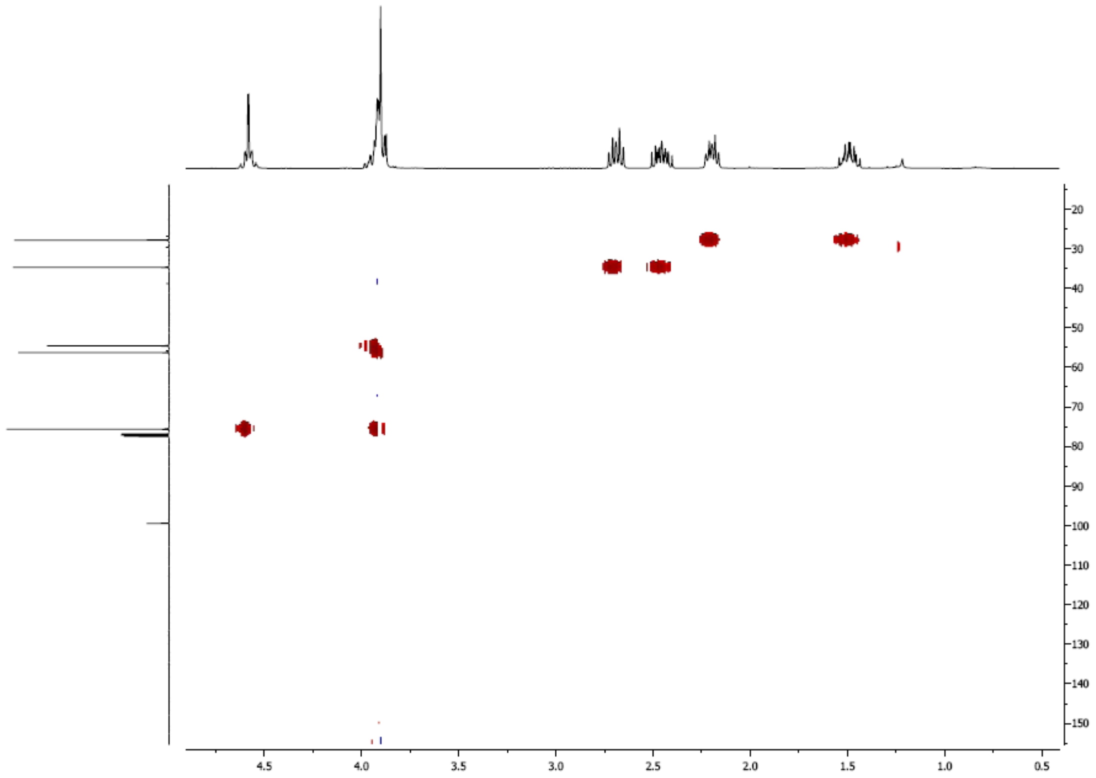

^{1}H-^{13}C HMBC (400MHz)

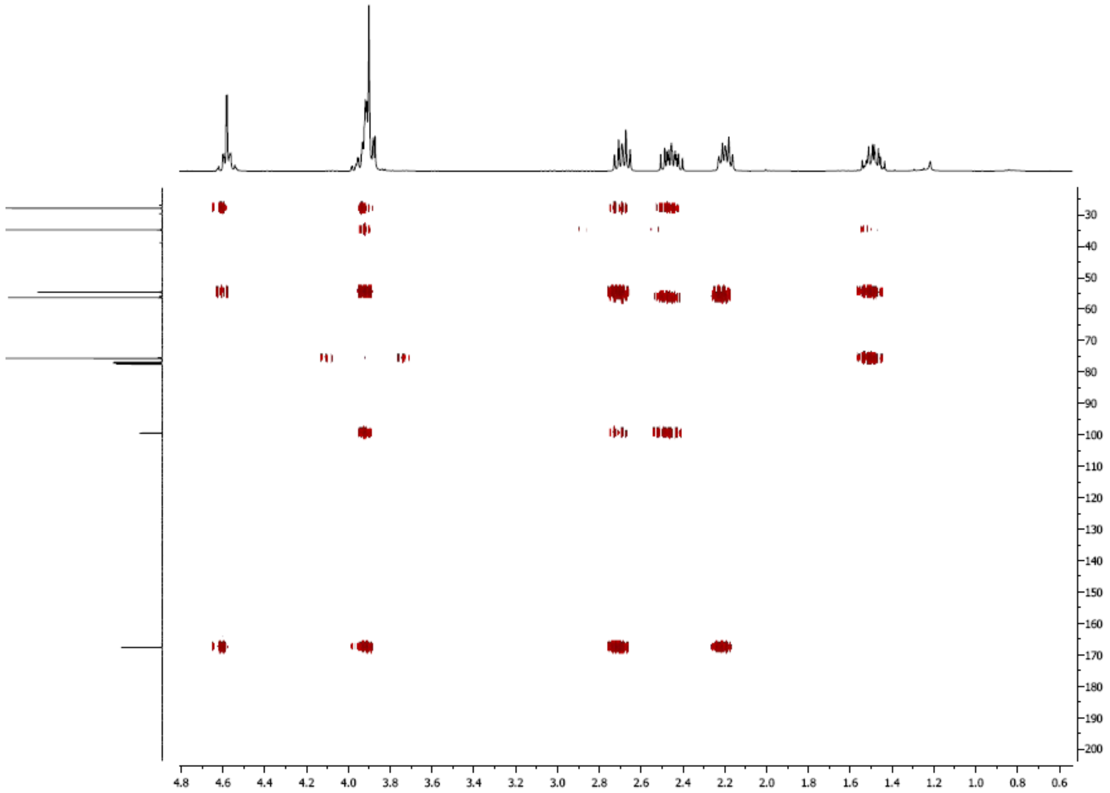

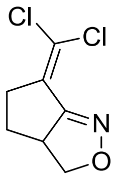
Cl
Cl
N
O

2r'

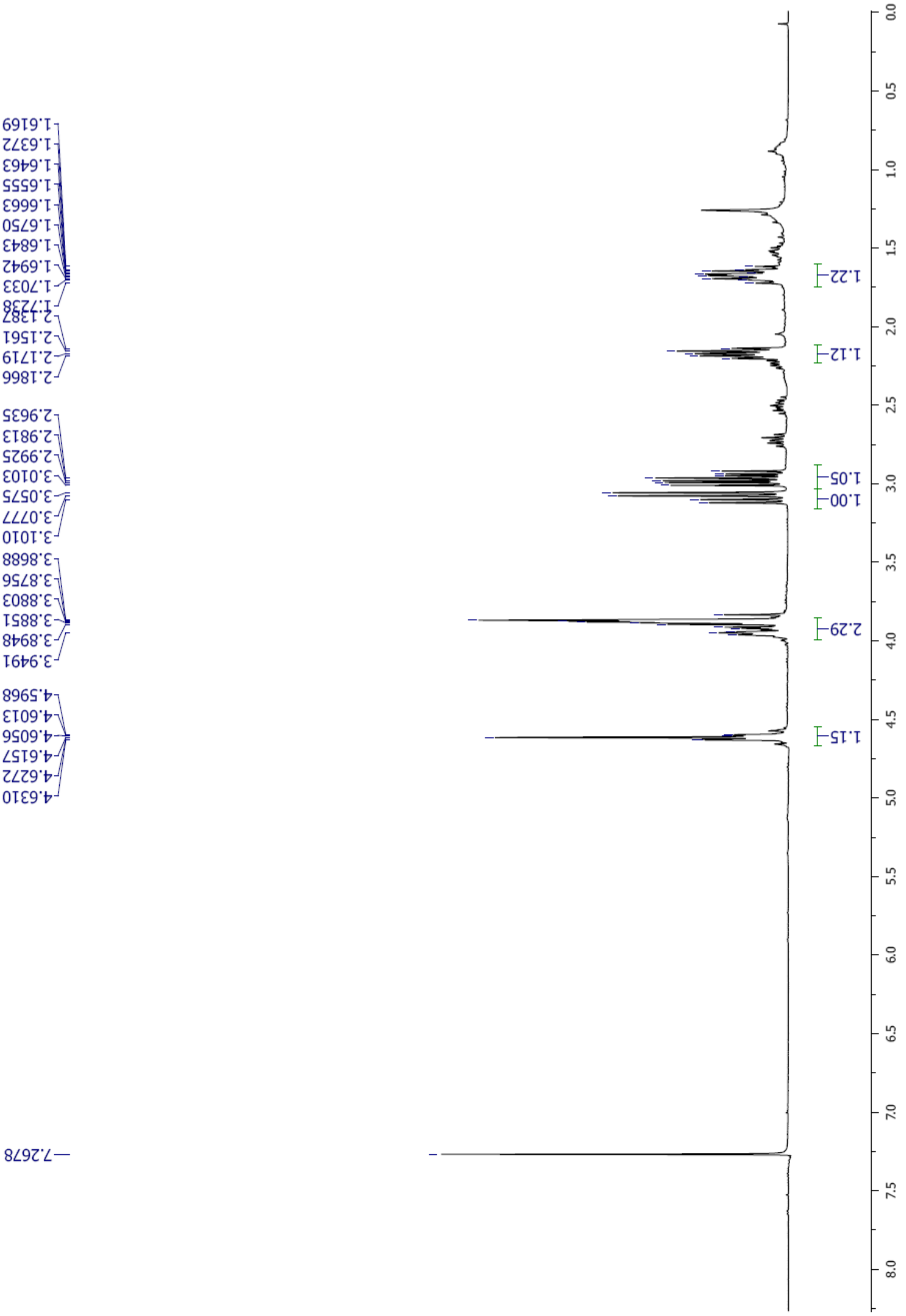
1.6169
1.6372
1.6463
1.6555
1.6663
1.6750
1.6843
1.6942
1.7033
1.7238
2.1387
2.1561
2.1719
2.1866
2.9635
2.9813
2.9925
3.0103
3.0575
3.0777
3.1010
3.8688
3.8756
3.8803
3.8851
3.8948
3.9491
4.5968
4.6013
4.6056
4.6157
4.6272
4.6310
7.2678
1.22
1.12
1.05
1.00
2.29
1.15
0.0
0.5
1.0
1.5
2.0
2.5
3.0
3.5
4.0
4.5
5.0
5.5
6.0
6.5
7.0
7.5
8.0

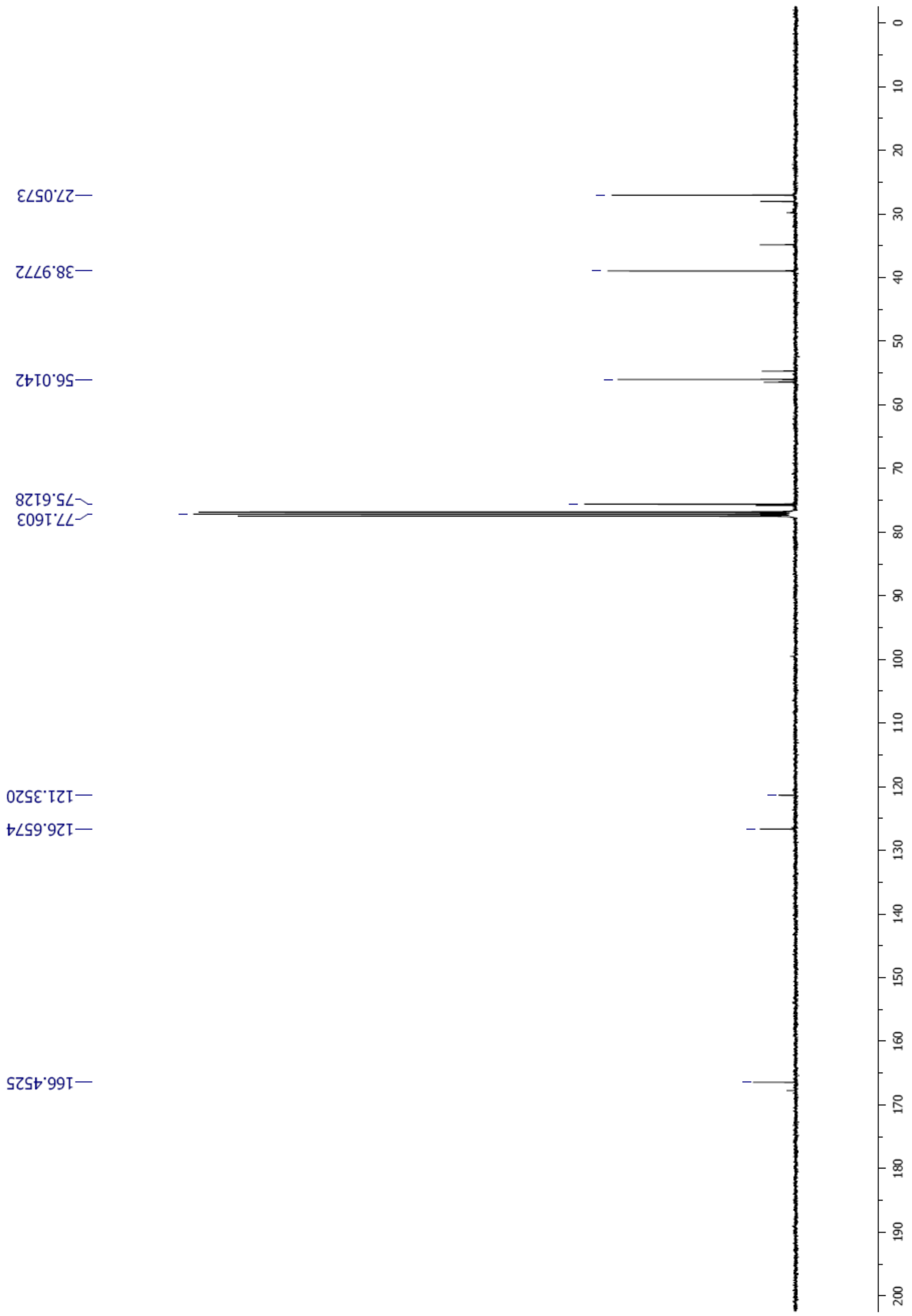
27.0573
38.9772
56.0142
75.6128
77.1603
121.3520
126.6574
166.4525
0
10
20
30
40
50
60
70
80
90
100
110
120
130
140
150
160
170
180
190
200

^{1}H-^{1}H COSY (400 MHz)

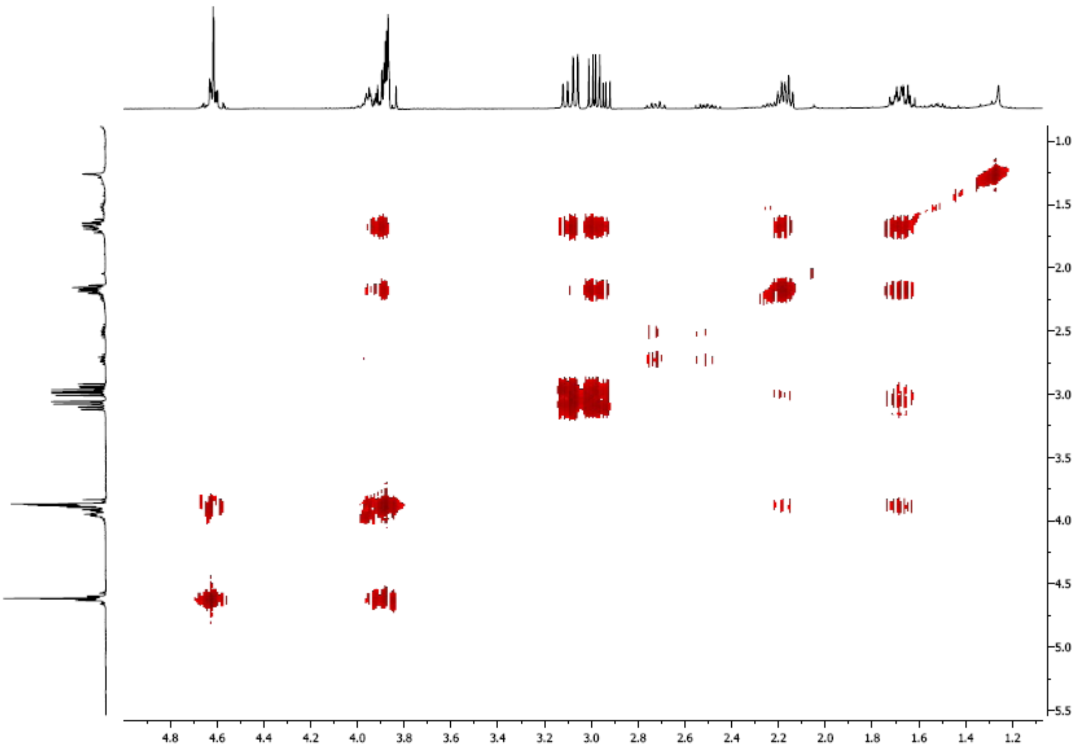

DEPT 135 C (100 MHz)

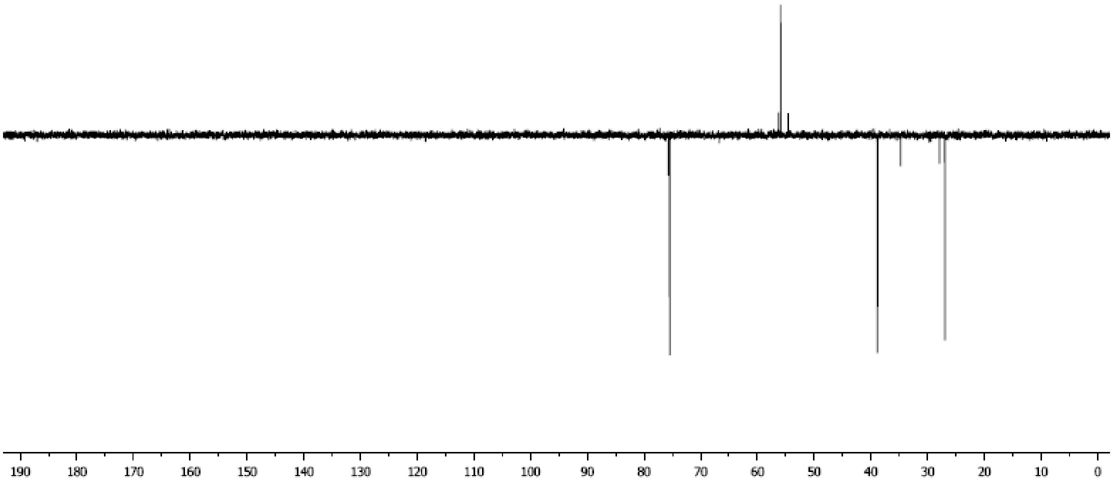

^{1}H-^{13}C HSQC (400MHz)

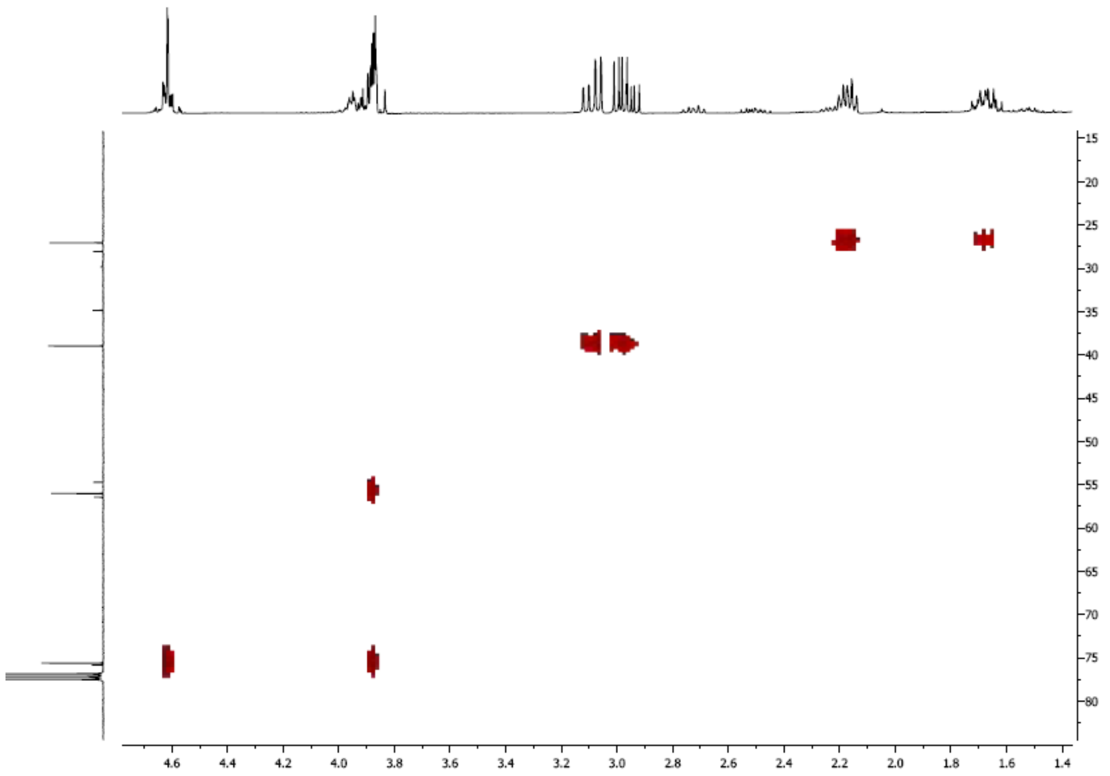

^{1}H-^{13}C HMBC (400 MHz)

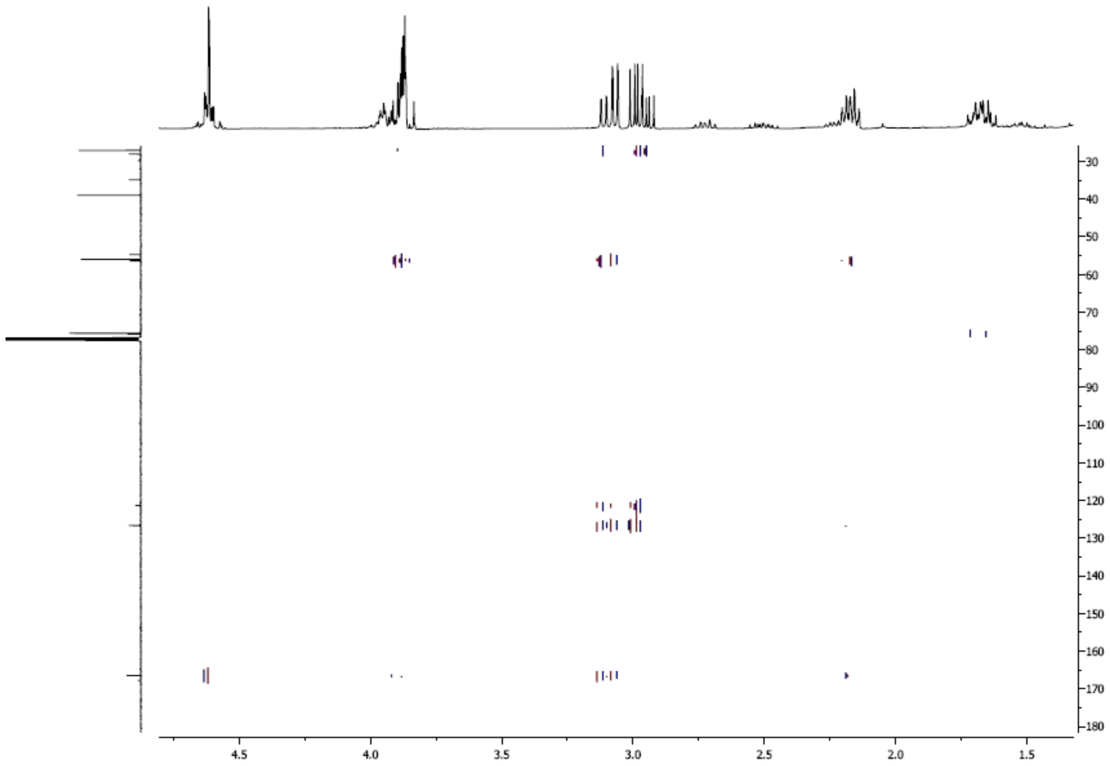

9

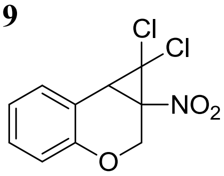
Cl
Cl
NO2
O

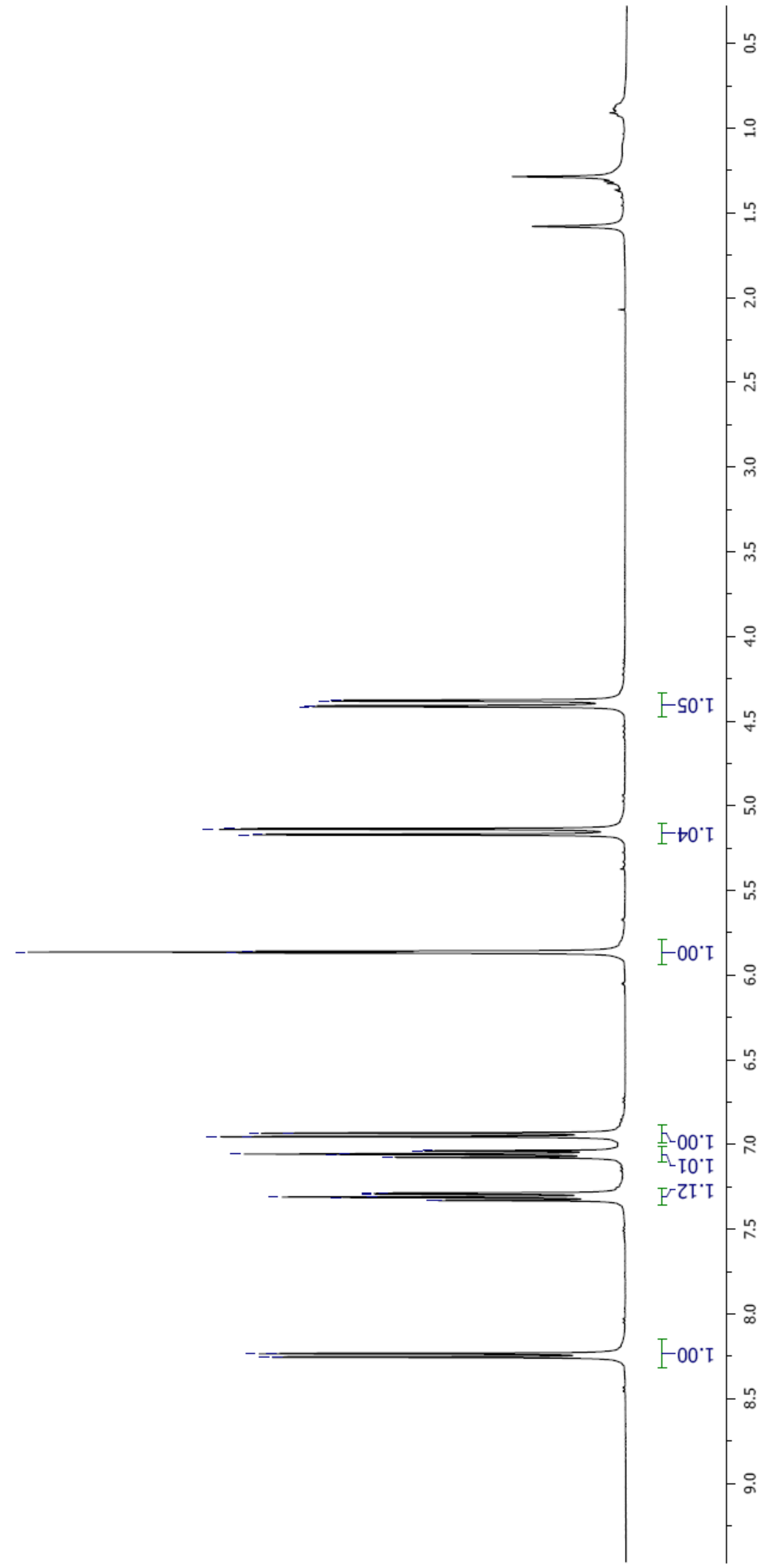
4.3757
4.3824
4.4083
4.4150
5.1340
5.1394
5.1666
5.1720
5.8573
5.8636
5.8695
6.9302
6.9335
6.9514
6.9542
7.0535
7.0561
7.0584
7.2844
7.2883
7.2921
7.3066
7.3100
7.3127
8.2320
8.2356
8.2525
8.2561
1.05
1.04
1.00
1.00
1.01
1.12
1.00
0.5
1.0
1.5
2.0
2.5
3.0
3.5
4.0
4.5
5.0
5.5
6.0
6.5
7.0
7.5
8.0
8.5
9.0

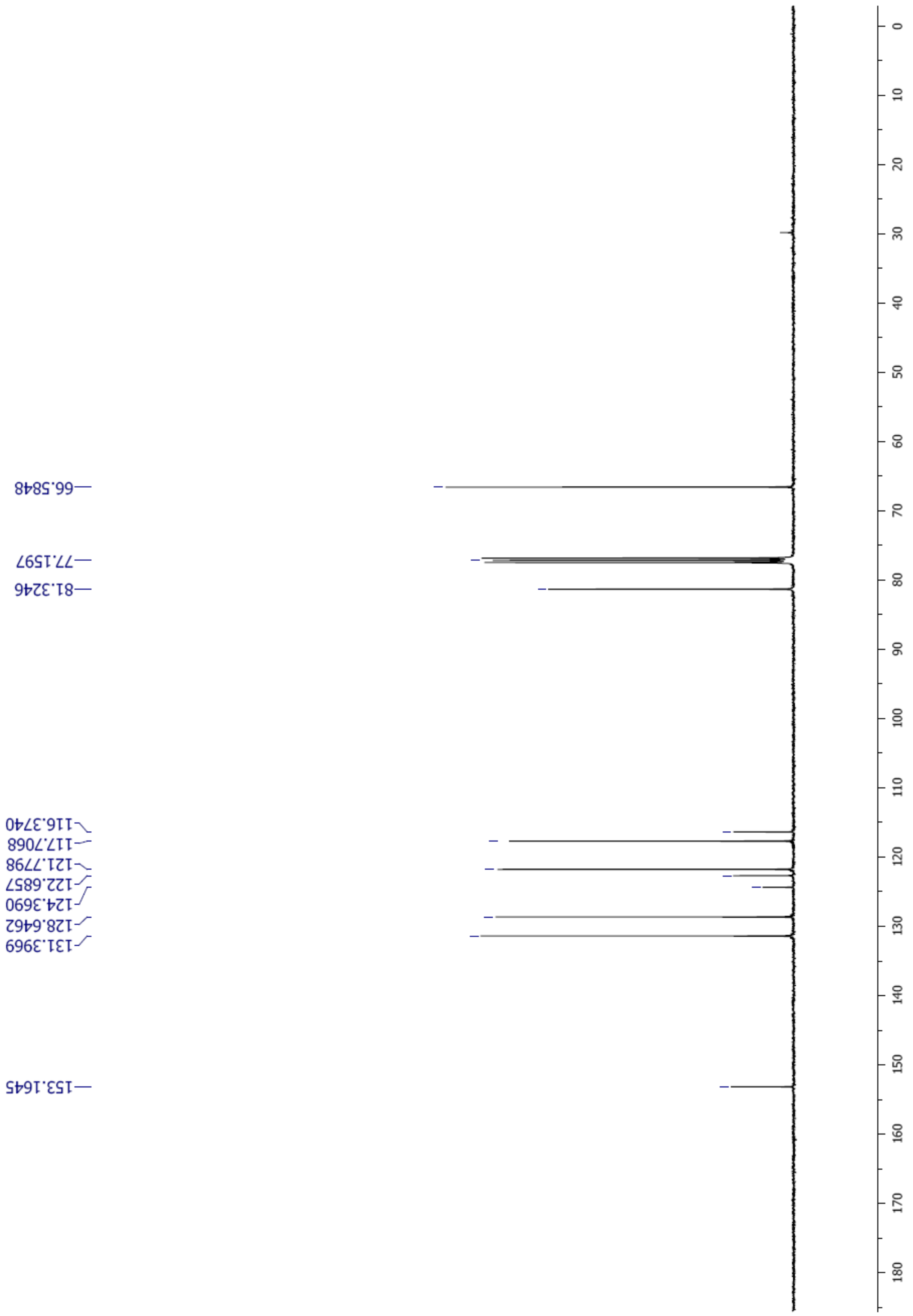
66.5848
77.1597
81.3246
116.3740
117.7068
121.7798
122.6857
124.3690
128.6462
131.3969
153.1645
0
10
20
30
40
50
60
70
80
90
100
110
120
130
140
150
160
170
180

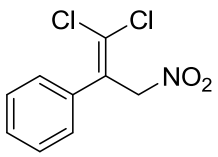

10

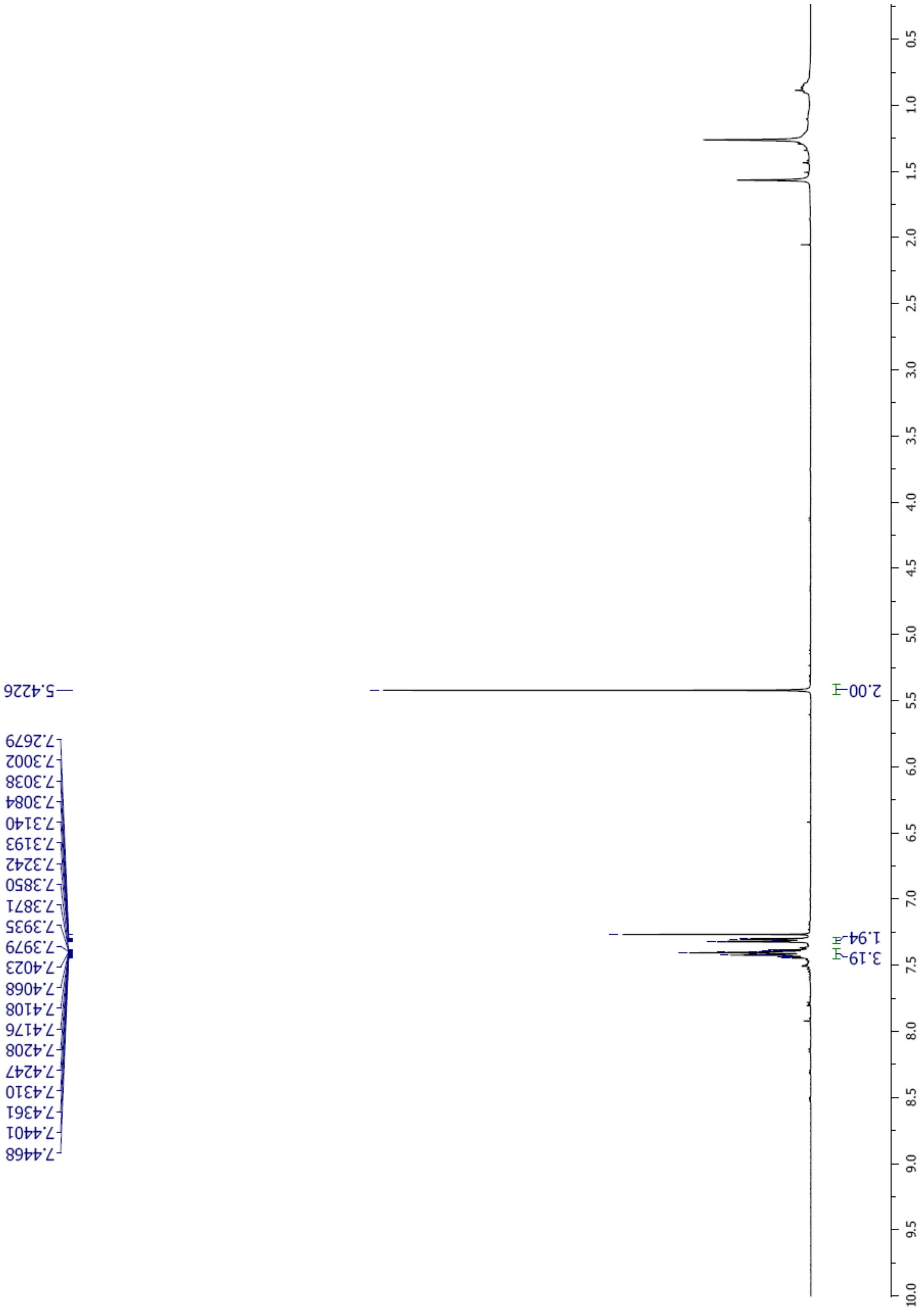
5.4226
7.2679
7.3002
7.3038
7.3084
7.3140
7.3193
7.3242
7.3850
7.3871
7.3935
7.3979
7.4023
7.4068
7.4108
7.4176
7.4208
7.4247
7.4310
7.4361
7.4401
7.4468
2.00
1.94
3.19
0.5
1.0
1.5
2.0
2.5
3.0
3.5
4.0
4.5
5.0
5.5
6.0
6.5
7.0
7.5
8.0
8.5
9.0
9.5
10.0

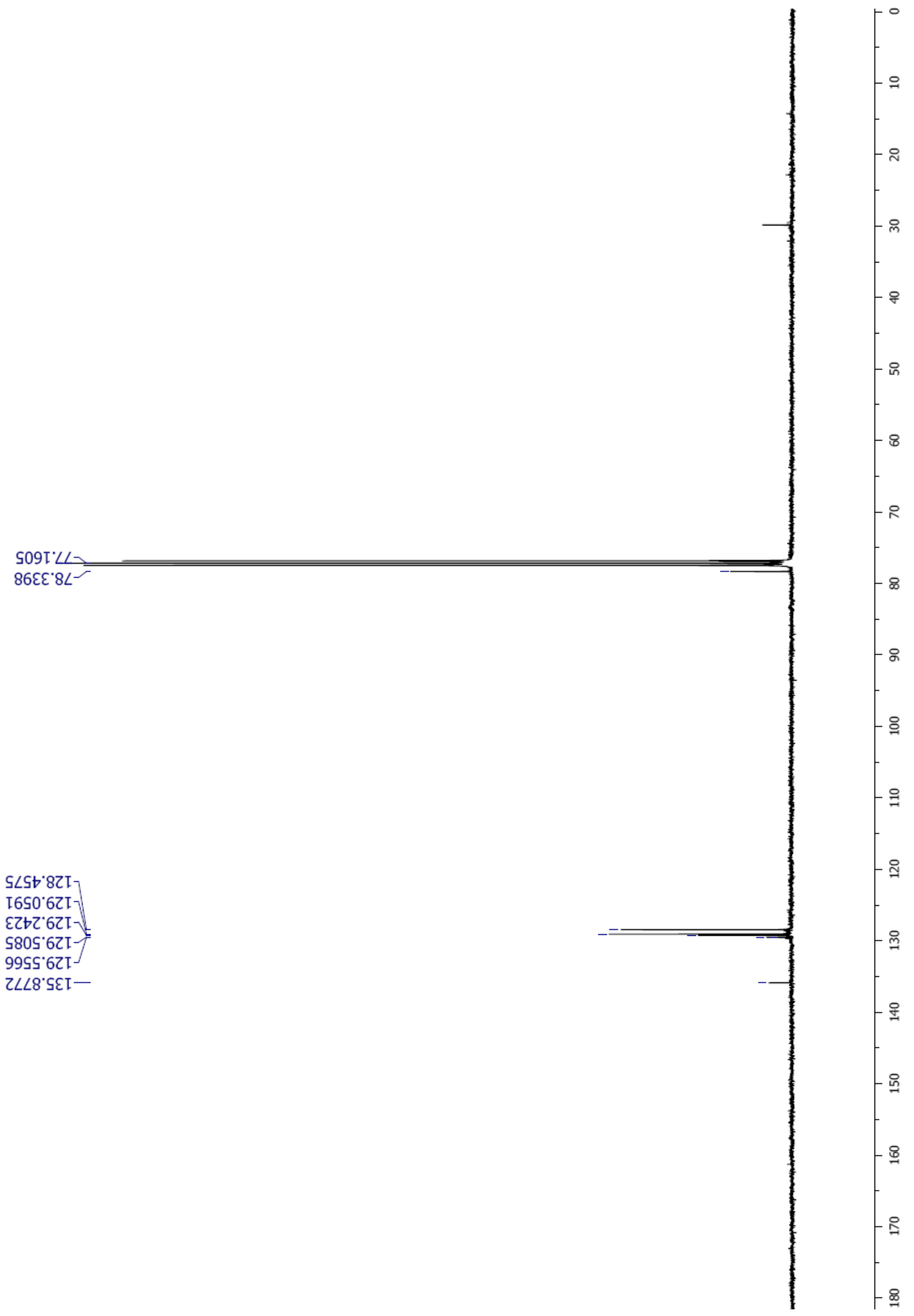
77.1605
78.3398
128.4575
129.0591
129.2423
129.5085
129.5566
135.8772

6. X-ray crystallography

Summary experimental for data collection

Data were collected by the School of Chemistry, University of Nottingham Crystallography Service using the standard methods described in the compound CIF files. The structures were solved and refined by standard methods. CIF files of the two compounds analyzed (**2d** and **2r'**) have been uploaded to the Cambridge Crystallographic Database with codes CCDC 988540 and CCDC 988541. respectively.
